# Design, Synthesis, and Cytotoxic Analysis of Novel Hederagenin–Pyrazine Derivatives Based on Partial Least Squares Discriminant Analysis

**DOI:** 10.3390/ijms19102994

**Published:** 2018-09-30

**Authors:** Kang Fang, Xiao-Hua Zhang, Yao-Tian Han, Gao-Rong Wu, De-Sheng Cai, Nan-Nan Xue, Wen-Bo Guo, Yu-Qin Yang, Meng Chen, Xin-Yu Zhang, Hui Wang, Tao Ma, Peng-Long Wang, Hai-Min Lei

**Affiliations:** School of Chinese Pharmacy, Beijing University of Chinese Medicine, Beijing 100102, China; 18364166994@163.com (K.F.); xhua_zhang@sina.com (X.-H.Z.); hanyaotiangz@163.com (Y.-T.H.); gaorongwu09@163.com (G.-R.W.); cai1225366978@163.com (D.-S.C.); 18810612758@163.com (N.-N.X.); wb_guo@126.com (W.-B.G.); yangyq5@163.com (Y.-Q.Y.); chenmeng_0809@163.com (M.C.); xinyums@126.com (X.-Y.Z.); 15652387323@163.com (H.W.); mosesmatao@163.com (T.M.)

**Keywords:** hederagenin, PLS-DA, antitumor activity, pyrazine, apoptosis

## Abstract

Hederagenin (**He**) is a novel triterpene template for the development of new antitumor compounds. In this study, 26 new **He**–pyrazine derivatives were synthetized in an attempt to develop potent antitumor agents; they were screened for in vitro cytotoxicity against tumor and non-tumor cell lines. The majority of these derivatives showed much stronger cytotoxic activity than **He**. Remarkably, the most potent was compound **9** (half maximal inhibitory concentration (IC_50_) was 3.45 ± 0.59 μM), which exhibited similar antitumor activities against A549 (human non-small-cell lung cancer) as the positive drug cisplatin (**DDP**; IC_50_ was 3.85 ± 0.63 μM), while it showed lower cytotoxicity on H9c2 (murine heart myoblast; IC_50_ was 16.69 ± 0.12 μM) cell lines. Compound **9** could induce the early apoptosis and evoke cell-cycle arrest at the synthesis (S) phase of A549 cells. Impressively, we innovatively introduced the method of cluster analysis modeled as partial least squares discriminant analysis (PLS-DA) into the structure–activity relationship (SAR) evaluation, and SAR confirmed that pyrazine had a profound effect on the antitumor activity of **He**. The present studies highlight the importance of pyrazine derivatives of **He** in the discovery and development of novel antitumor agents.

## 1. Introduction

**Figure 1:**
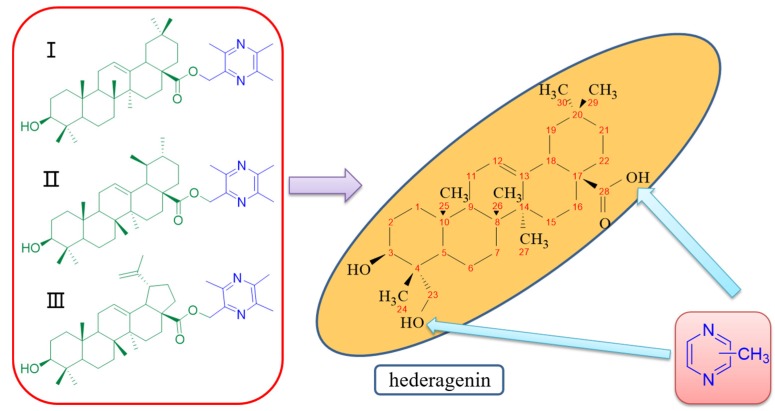
Structural modification of hederagenin (**He**).

Natural molecules are an indispensable source of lead compounds for drug discovery [1,2,3]. Pentacyclic triterpenes are widely present in medicinal plants, and they show a unique range of pharmacological activities, including great antitumor, anti-inflammatory, and antiviral activities [4,5,6]. They attracted the attention of scholars due to their enormous chemopreventive and antineoplastic potential [7,8]. In view of this, the structural modification of pentacyclic triterpenoids is always a focus in drug research [9,10,11]. Hederagenin (**He**, 3b,23-dihydroxyurs-12-ene-28-oic acid; Figure 1) is a member of the pentacyclic triterpenoid family, which is extracted mainly from the medicinal plant ivy (*Hedera helix* L.) and *Dipsacus asper* [12,13]. A variety of pharmacological activities were ascribed to **He**, such as anticancer [14], anti-inflammatory [15], neuroprotective [16], and antiatherosclerosis effects [17]. Notably, **He** exhibits potential antitumor activity in several cancer cell lines. **He** could induce apoptotic cell death in LoVo and breast cancer cells through regulation of the mitochondrial apoptosis pathway [18,19], as well as evoke apoptosis in cisplatin-resistant head and neck cancer cells by inhibiting the nuclear factor erythroid-2-related factor 2/antioxidant responsive element (Nrf2–ARE) pathway [15].

Numerous derivatization studies performed on **He** exhibited excellent anticancer activities. A previous study demonstrated that **He** derivatives carrying a heterocyclic group at C-28 had strong cell-growth-inhibitory activity against several cancer cell lines (half maximal inhibitory concentration (IC_50_) ranging from 1.1 to 6.5 µM) [20]. Furthermore, **He** derivatives with aryl-1*H*-1,2,3-triazol-4-yl-methyl esters at C-28 have stronger cell-growth-inhibitory activity than the amides against several cancer cell lines [21]. The above literature studies suggested that **He** derivatives with heterocyclic group esters at C-28 might exhibit excellent antitumor activities. However, the number of **He** derivatives that was synthesized and investigated with respect to antitumor activity is still quite limited. Therefore, there is a great interest in the synthesis and evaluation of novel **He** derivatives in order to develop more potential antitumor drugs. Pyrazine is an *N*-heteroaromatic ring, and the incorporation of *N*-heteroaromatic rings into organic molecules could improve their antitumor activity and aqueous solubility [10,22,23]. Our research group proved that the introduction of ligustrazine to betulinic acid, ursolic acid, glycyrrhetinic acid, and oleanolic acid could significantly improve their antitumor effects in vitro [24,25]. Among them (Figure 1), compound 3β-hydroxyolea-12-en-28-oic acid-3,5,6-trimethylpyrazin-2-methyl ester (I), 3β-hydroxyurs-12-en-28-oic acid-3,5,6-trimethylpyrazin-2-methyl ester (II), and 3β-hydroxy-lup-20(29)-ene-28-oic acid-3,5,6-trimethylpyrazin-2-methyl ester (III) exhibited significant cytotoxicity against a variety of tumor cell lines. As a consequence of the desirable properties of *N*-heteroaromatic rings, *N*-heteroaromatic rings became very important in the design and development of new drugs. However, to the best of our knowledge, there is no systematic literature concerning pyrazines with different methyl numbers and positions in the aforementioned field. In this paper, this issue was discussed preliminarily. In addition, the length of the carbon chain also had a great influence on biological activity [26,27]. It could enrich compound diversity and provide more possibilities for the discovery of lead drugs by introducing carbon chains.

The structure–activity relationship (SAR) evaluation of novel compounds plays a vital role in discovering new drugs. Thus, finding and applying a rapid and trustworthy method is needed. Partial least squares discriminant analysis (PLS-DA) is a linear classification method that combines the discrimination power of a classification technique with the properties of partial least squares regression. PLS-DA models could classify samples on the basis of calculating class thresholds on the basis of the Bayes theorem (samples can be unclassified) or class probabilities (samples are assigned to a class), and it was widely used to judge how to classify research objects [28,29]. Compared with direct observation, the introduction of PLS-DA into the structure–activity relationship evaluation could reflect the structure–activity law more intuitively. In this paper, we provided a new reference for structure–activity relationship analysis by applying cluster analysis to structure–activity analysis.

In line with these fascinating features, we reported 26 **He**–pyrazine derivatives. Their chemical structures were ingeniously designed, and they exhibited certain regularity. Based on the SAR, we preliminarily clarified the law of structure–activity on the antitumor effect of **He** in vitro, including pyrazine with different numbers and positions of methyl groups, with a variable length of carbon chain as a linker, as well as a modification site. All new derivatives were screened for in vitro cytotoxicity against tumor and non-tumor cell lines including HepG2, A549, MCF-7, H9c2, and MDCK cells. Moreover, SAR was analyzed by PLS-DA. The result of SAR provided a reference for the modification of **He** for subsequent research. Furthermore, fluorescence staining observations and flow cytometric analyses were performed to investigate the potential mechanism of action of the most active compound **9** in A549 cells.

## 2. Result and Discussion

### 2.1. Chemistry

Based on the structure of **He** and some reports [30,31], we chose available sites for chemical modification to improve antitumor activity at C-28 and C-23, and we designed 26 compounds by combining a pyrazine ring (Figure 1). Firstly, hederagenin (**He**) was isolated from the himalayan teasel root as detailed in the experimental section.

**Scheme 1:**
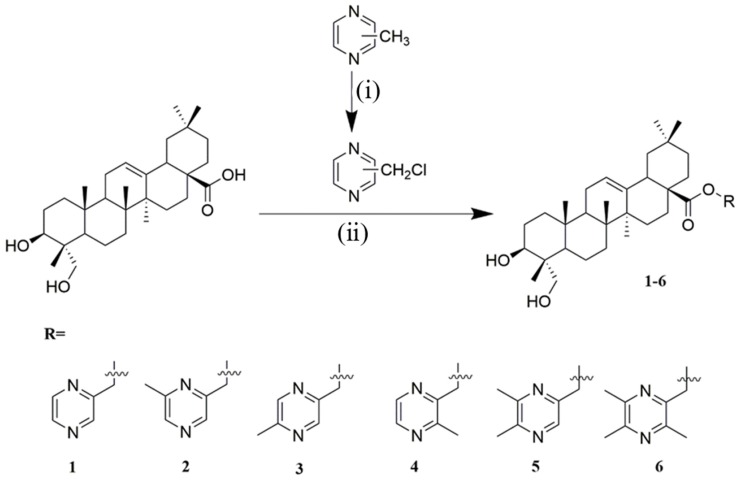
Synthesis of hederagenin (**He**) derivatives **1**–**6**. Reagents and conditions: (**i**) *N*-chlorosuccinimide (NCS), benzoyl peroxide (BPO), carbon tetrachloride (CCl_4_), N_2_, 60 °C, 8 h, with 60–80% yield; (**ii**) *N,N*-dimethylformamide (DMF), K_2_CO_3_, 85 °C, 4 h, with 35–90% yield.

**Scheme 2:**
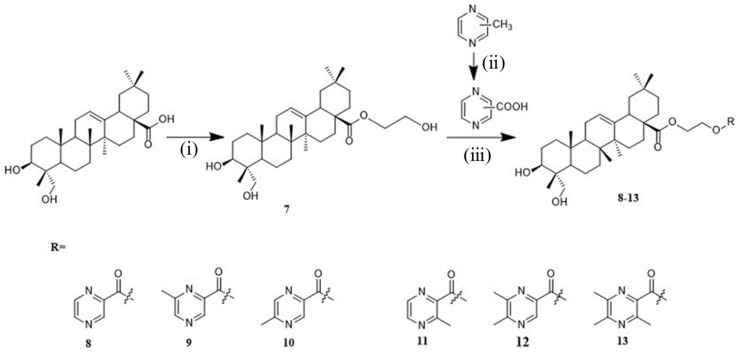
Synthesis of **He** derivatives **7**–**13**. Reagents and conditions: (**i**) 2-bromoethanol, DMF, K_2_CO_3_, 85 °C, 4 h, with 95% yield; (**ii**) KMnO_4_, H_2_O, 75 °C, 2 h, with 60–85% yield; (**iii**) CH_2_Cl_2_, 1-ethyl-3-(3-dimethylaminopropyl) carbodiimide hydrochloride (EDCI), 4-dimethylaminopyridine (DMAP), room temperature (rt), 12 h, with 20–90% yield.

**Scheme 3:**
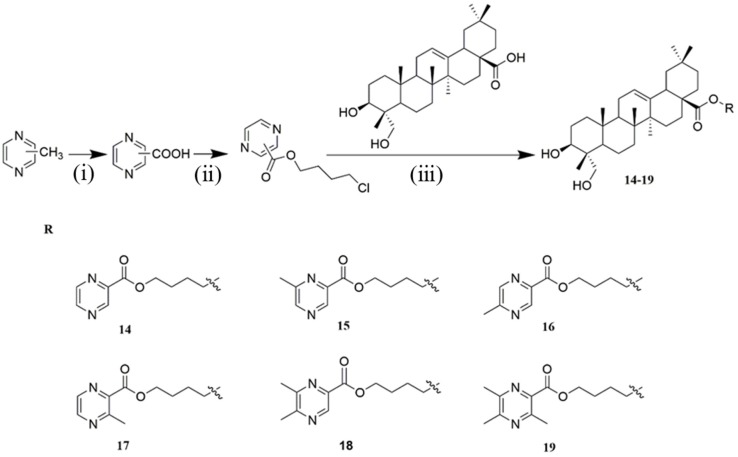
Synthesis of **He** derivatives **14**–**19**. Reagents and conditions: (**i**) KMnO_4_, H_2_O, 75 °C, 2 h, with 60–85% yield; (**ii**) CH_2_Cl_2_, 4-chlorobutanol, EDCI, DMAP, rt, 12 h, with 75–95% yield; (**iii**) DMF K_2_CO_3_, 75 °C, 4 h, with 30–90% yield.

**Scheme 4:**
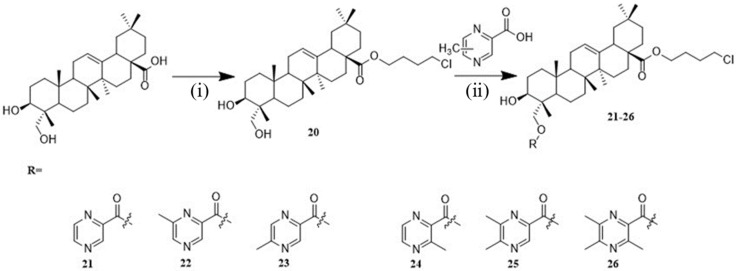
Synthesis of **He** derivatives **20**–**26**. Reagents and conditions: (**i**) DMF, K_2_CO_3_, 4-chlorobutanol, 75 °C, 4 h, with 54% yield; (**ii**) CH_2_Cl_2_, EDCI, DMAP, rt, 12 h, with 30–90% yield.

As shown in Scheme 1, Scheme 2, Scheme 3 and Scheme 4, all the designed derivatives were synthesized [20,24]. In Scheme 1, six kinds of chloromethyl pyrazines were produced by *N*-chlorosuccinimide (NCS) and benzoyl peroxide (BPO) in carbon tetrachloride (CCl_4_) under light conditions with nitrogen protection, and the yield ranged from 40–60%. Then, compounds **1**–**6** were obtained through a combination of **He** and chloromethyl pyrazines in *N,N*-dimethylformamide (DMF) containing K_2_CO_3_ at 85 °C, and the yield was 35–90%. As seen in Scheme 2, compound **7** was obtained from **He** with 2-bromoethanol in DMF containing K_2_CO_3_ at 85 °C with 95% yield. Then, compounds **8**–**13** were produced with 20–90% yield through a combination of **He** and pyrazinic acid with methyl groups using 1-ethyl-3-(3-dimethylaminopropyl) carbodiimide hydrochloride (EDCI) and 4-dimethylaminopyridine (DMAP) in dry dichloromethane (DCM). In addition, pyrazinic acid with methyl groups was obtained from pyrazine with a methyl group in H_2_O with KMnO_4_, and the yield was 70–90%. Interestingly, compounds **20**–**26** were obtained with 30–90% yield using a similar method to that shown in Scheme 2. In this reaction, the hydroxyl group of 4-chlorobutanol reacted with the carboxyl group of **He** instead of methyl chloride. As described in Scheme 3, the intermediate was obtained by combining pyrazinic acid with methyl groups with 4-chlorobutanol in EDCI and DMAP in DCM, and the yield was 80–95%; then, compounds **14**–**19** were produced with 30–90% yield through a combination of **He** and the intermediate under K_2_CO_3_ in DMF at 85 °C. Interestingly, according to the original routine, we planned on gaining compounds **14**–**19** with the synthesis conditions of Scheme 4; however, compounds **21**–**26** were obtained unexpectedly. Based on the data, we redesigned the experimental steps as Scheme 3, and obtained compounds **14**–**19** successfully. All reactions were carried out as detailed in the experimental section, which also describes the spectra for all compounds (spectra data can be found in Supplementary Materials) [20,32,33,34].

### 2.2. Biological Screening

#### 2.2.1. Cytotoxicity Assay

The cytotoxicity of hederagenin derivatives in vitro was evaluated on three tumor cell lines (A549: human non-small-cell lung cancer; MCF-7: human mammary cancer; HepG2: human hepatocellular carcinoma) using the 3-(4,5-dimethylthiazol-2-yl)-2,5-diphenyltetrazolium bromide (MTT) assay. In addition, their toxicity evaluations were tested on H9c2 (murine heart myoblast) and MDCK (Madin–Darby canine kidney) cells. The IC_50_ values of these compounds are summarized in Table 1.

**Figure 2:**
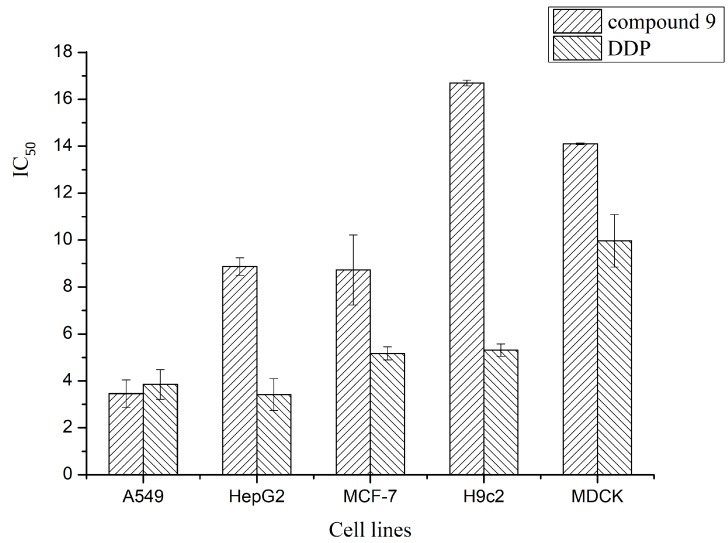
Comparison of cytotoxicity between compound **9** and cisplatin (**DDP**).

Many of the derivatives displayed more significant growth inhibition effects than **He**. Remarkably, as shown in Figure 2, the most promising was compound **9**, which exhibited similar antitumor activities against A549 as the positive drug cisplatin (**DDP**), while it showed lower cytotoxicity than **DDP** on MDCK and H9c2 cell lines.

#### 2.2.2. Cluster Analysis

##### Principal Component Analysis (PCA)

A PCA study was designed to verify the adaptability of experimental data to the model. For the initial overview of the dataset and the detection of trends and outliers, PCA was carried out. Analysis showed no samples being outside the Hotelling T^2^ 95% confidence ellipse that could influence the analyses, and high values of explained variation and predictive ability were obtained (Table 2). All data were analyzed using SIMACA 13.0.

##### Partial Least Squares Discriminant Analysis (PLS-DA)

**Figure 3:**
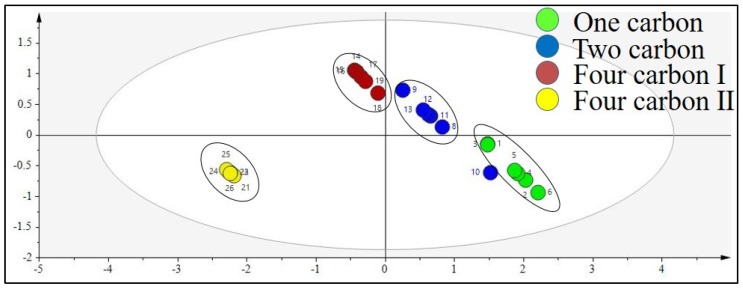
Partial least squares discriminant analysis (PLS-DA) of carbon chain length on HepG2 (human hepatocellular carcinoma). Four carbon I—a carbon chain of four carbon atoms and pyrazine were combined at the C-28 position; Four carbon II—a carbon chain of four carbon atoms was added at the C-28 position, but pyrazine was added at the C-23 position.

**Figure 4:**
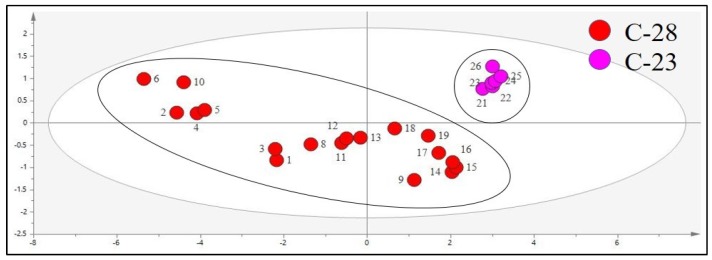
PLS-DA of combining position on HepG2. C-28—pyrazine was added at the C-28 position; C-23—pyrazine was added at the C-23 position.

To further explore the structure–activity relationship, PLS-DA was performed, comparing all designed pairs of groups of **He** derivatives. Analyses revealed an antitumor activity discrimination between the different types of **He** derivatives compared in pairs. As Figure 3 and Figure 4 depict, **He** derivatives combined with the same pyrazine exhibited similar in vitro antitumor activities, and the length of the carbon chain at the attachment site caused a difference in activity. In different tumor cell models, **He** derivatives linked with one carbon, two carbons, and four carbons were divided into three groups according to the difference in in vitro antitumor activity (Figure 3). As for the effect of the structure modification site of **He**, **He** derivatives modified at C-28 and C-23 were divided into two groups according to the difference in in vitro antitumor activity (Figure 4). It is worth noticing that this put forward a reference point for the selection of structural modification sites for **He**. Through data analysis, we found that the number of methyl groups on the pyrazines also showed a certain degree of regularity in their effect on activity. There was no law between the position of methyl groups on pyrazines and antitumor activity. Pyrazine derivatives with different numbers of methyl groups were divided into six groups according to the difference in in vitro antitumor activity. Incredibly, the analysis results of cluster analysis were consistent with the results of direct observation. For example, compounds **21**–**26** (similar activities on HepG2, IC_50_ ranging from 6.42 to 7.99 μM) could be separated from the others (IC_50_ > 8 μM on HepG2) by direct observation. Similarly, the results of cluster analysis of PLS-DA supported this result (Figure 4). The cluster analysis of PLS-DA might provide us with further directions for further analysis of **He** derivatives.

##### Structure–Activity Relationship Analysis

Combining the data analysis from Section 2.2.1 and Section Partial Least Squares Discriminant Analysis (PLS-DA), we could easily find that the structural modification site of **He**, the length of the carbon chain, and the type of pyrazine had an effect on the in vitro antitumor activity of the **He** derivatives. In general, as observed for compounds **14**–**19** and **21**–**26**, structural modification at positions C-28 and C-23 could improve antitumor biological activity in vitro, while structural transformation of C-23 might have more potential to enhance cytotoxicity on the same series of tumor cells; it was confirmed that the IC_50_ values of compounds **21**–**26** were usually lower than those of compounds **14**–**19**. In general, compounds with chains containing two and four carbons were superior to those with one carbon for improving the antitumor activity of the compounds. For example, compounds **14**–**19** with a chain containing four carbons generally exhibited superior antitumor activity to others, especially on HepG2. **He** derivatives **2**, **9** and **15** with 2,6-dimethylpiperazine showed better antitumor activity in vitro than others. In addition, comparing compounds **7** and **20** with others, we knew that the introduction of the pyrazine structure was essential for improving the in vitro antitumor activity of **He**. Notably, compound **9** (**He** combined with 2,6-dimethylpiperazine via a chain containing two carbon atoms), the drug with the most potential, was also in line with this law. It is worth mentioning that compounds **14**–**19** and **21**–**26** exhibited good antitumor activity and some cytotoxic selectivity toward HepG2 cells in vitro (the selective inhibition (SI = IC_50_^MCF-7^/IC_50_^HepG2^) value of compound **14** was 4.43). What these compounds had in common was that they were modified by four-carbon chains. This meant that changes in the length of the carbon chain at C-28 and C-23 might alter the selectivity of compounds for different cell lines; we speculated that this result might be caused by the combination of the compound and the tumor cell target.

#### 2.2.3. Analyses of Apoptosis

##### Morphological Detection of Apoptosis Using Giemsa Staining

**Figure 5:**
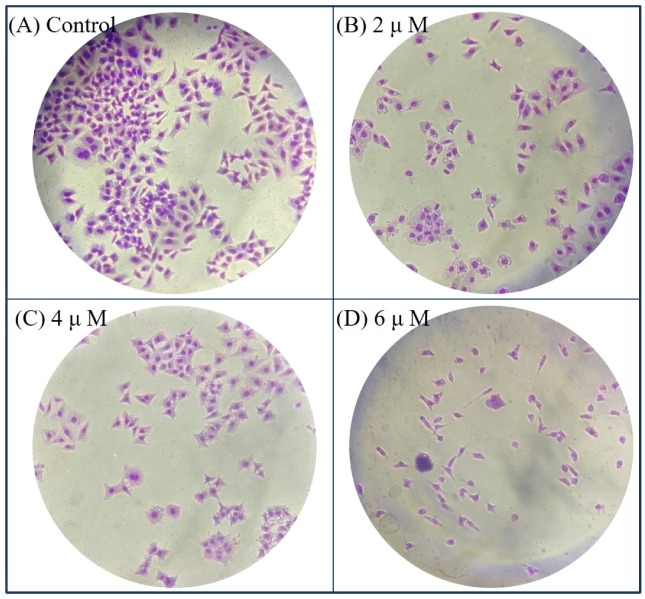
Morphological detection of apoptosis using Giemsa staining (200×) on A549 (human non-small-cell lung cancer) cells treated with compound **9**: (**A**) control group; (**B**) 2 μM; (**C**) 4 μM; (**D**) 6 μM.

To characterize the morphological detection of apoptosis induced by compound **9** on A549, the nuclear and cytoplasmic morphological changes in compound-**9**-treated A549 were observed with Giemsa staining. As shown in Figure 5, the number of A549 cells in the control group was higher than other groups, and the cells appeared polygonal with a normal cell shape. With an increase in dose, the phenomenon of cell shrinkage and cell disruption became more obvious. At the dose of 2 μM, the cells had a lot of puffing and fragmentation. When the concentration was 6 μM, there was almost no normal cell morphology.

##### 4′,6-Diamidino-2-phenylindole (DAPI) Staining

**Figure 6:**
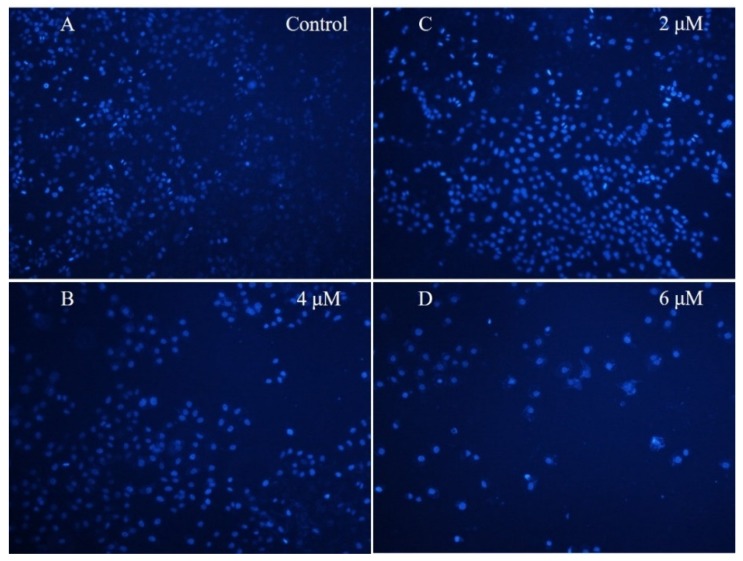
Morphological detection of apoptosis using 4′,6-diamidino-2-phenylindole (DAPI) staining (100×) on A549 cells treated with compound **9**: (**A**) control group; (**B**) 2 μM; (**C**) 4 μM; (**D**) 6 μM.

To characterize the effects of apoptosis inducted by compound **9** on A549, the nuclear morphological changes in compound-**9**-treated A549 were observed with DAPI staining. After treating with compound **9** for 72 h, A549 cells showed nuclear morphological changes typical of apoptosis in a dose-dependent manner. As pictured in Figure 6, in the control group, the nucleus was intact and evenly colored. The light-blue fluorescence was diffuse and the cells did not show the characteristics of apoptosis. When the concentration of the drug increased, nuclear condensation, nuclear fragmentation, and the formation of apoptotic bodies appeared. When treated at 6 μM, the number of cells decreased drastically, the shape of the cells became irregular, and nuclear fragmentation occurred.

##### Detection of Apoptosis Using Annexin V Fluorescein Isothiocyanate (FITC)/Propidium Iodide (PI) Staining

**Figure 7:**
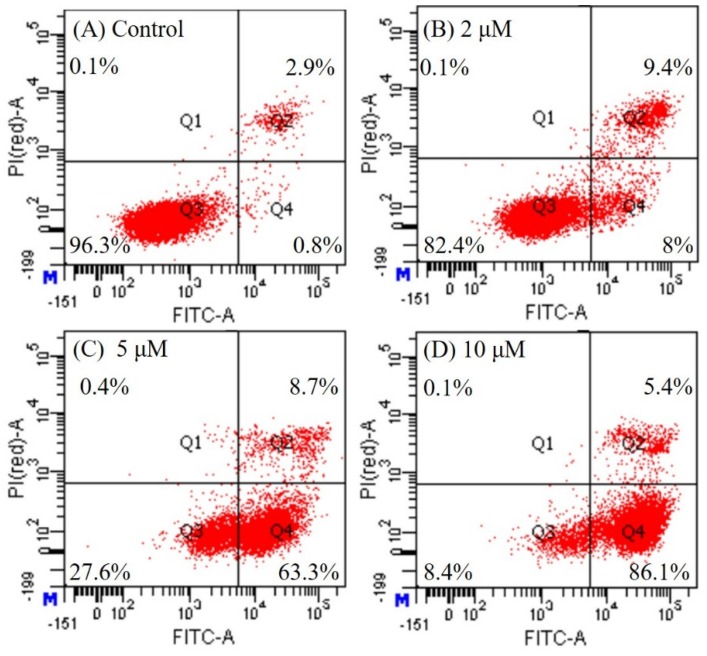
Detection of apoptosis using Annexin V fluorescein isothiocyanate (FITC)/propidium iodide (PI) staining on A549 cells treated with compound **9**: (**A**) control group; (**B**) 2 μM; (**C**) 5 μM; (**D**) 10 μM.

To further depict the apoptosis induced by compound **9**, apoptotic rates were analyzed by flow cytometry using Annexin V-FITC/PI staining. As depicted in Figure 7, with different concentrations (0, 2, 5, and 10 μM) of compound **9** treatment, the percentage of apoptotic cells (including early and late apoptosis ratios) increased from 3.7% of the control to 17.4%, 72.0%, and 91.5%, respectively. The result indicates that compound **9** had the potential to induce the apoptosis of A549 cells.

#### 2.2.4. Detection of the Effect of Compound **9** on Cell-Cycle Progression

**Figure 8:**
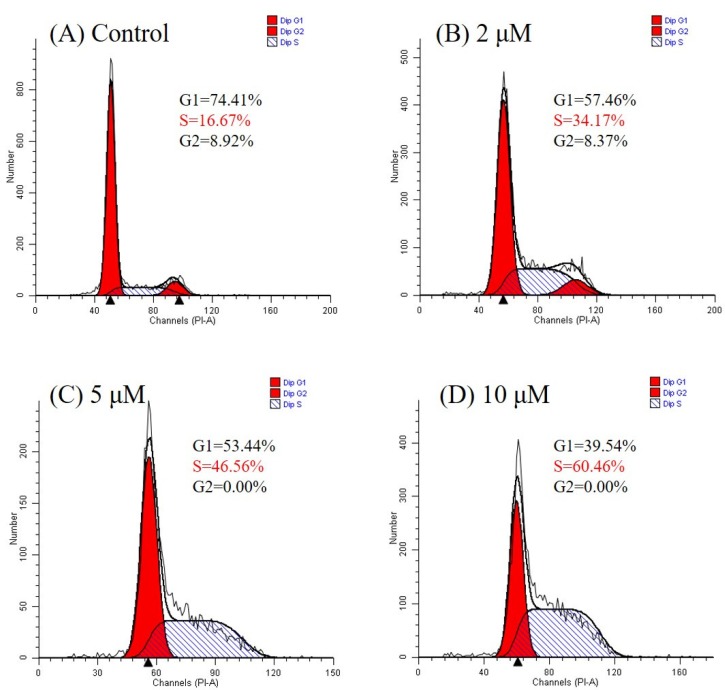
Cell-cycle distribution of A549 cell lines after treatment with compound **9** at different concentrations: (**A**) control group; (**B**) 2 μM; (**C**) 5 μM; (**D**) 10 μM.

The remarkable activity of compound **9** against the A549 cell line prompted us to investigate its effect on the cell cycle of A549 by flow cytometry. As shown in Figure 8, when the concentration increased (0, 2, 5, and 10 μM), the percentage of A549 cells in the synthesis (S) phase overtly increased (from 16.67% to 60.46%) which clearly indicated that compound **9** arrested the cells in the S phase in a concentration-dependent manner.

## 3. Discussion

A series of different esters of **He** were designed and synthesized. All these compounds were screened for cytotoxic activity employing a panel of five cell lines, including HepG2, A549, MCF-7, H9c2, and MDCK cells, using the MTT assay. From these data analyzed with PLS-DA, it was evident that almost all derivatives exhibited higher cytotoxicity for all tested cancer cell lines compared to **He**. Compound **9** was found to be the most likely drug candidate, showing IC_50_ values ranging from 3.22–4.48 µM. As shown by Giemsa, DAPI, and Annexin V-FITC/PI staining, it was found that compound **9** mainly acted by inducing early apoptosis. Moreover, the present research found that compound **9** could induce cell-cycle arrest at the S phase. After exploring the structure–activity relationship, we found that the introduction of a 2,6-dimethyl pyrazine structure with a linker containing 2–4 carbon atoms at position 23 of **He** might be more effective in enhancing the antitumor effect of the compound in vitro. Further intensive modifications at C-23 and studies are currently being performed in our research group, and the results will be reported in due course.

## 4. Materials and Methods

### 4.1. General Aspects

Chemical shifts (δ) are given in ppm and coupling constants (*J*) in Hz. Reagents were bought from commercial suppliers without any further purification. NMR spectra were recorded on a Bruker-500 or -400 spectrometer (Bruker, Dresden, Germany) with tetramethylsilane (TMS; TCI, Tokyo, Japan) as an internal standard; high-resolution mass spectra (HRMS) were acquired using a Thermo Sientific TMLTQ Orbitrap XL hybrid FTMS instrument (Thermo Technologies, New York, NY, USA), and an X-5 micro melting point apparatus (Beijing, China). 

#### 4.1.1. Isolation of Hederagenin (**He**)

Dried *Dipsacus asper* Wall.ex Henry was extracted with 70% ethanol; three extractions at refluxed temperature were carried out, and the solvent was kept in contact with the sample for 3–9 h at a concentration ratio of 10 to 20 times the amount of ethanol than medicinal herbs. The combined ethanol extracts were concentrated under reduced pressure in a rotary evaporator. Then, 7% hydrochloric acid solution in water (20 times the residue) was added to this residue, and the mixture was heated under reflux temperature for 4–8 h. After this hydrolysis, the residue was filtered off, and the precipitate was obtained, washed with water to neutrality, and dried at 65 °C to obtain a crude product of **He**. The crude product was purified by column chromatography on a silica gel with solvent (DCM:MeOH = 70:1), and the mixture was concentrated and evaporated to dryness. After standing overnight under 25–50 mL of anhydrous ethanol, the residue was filtered off, and **He** was obtained. Hederagenin (**He**): yield <10%; white powder, melting point (m.p.) >300 °C; HRMS (electrospray ionization (ESI)) *m/z*: [M + Cl]^−^ 507.3274, calculated for C_30_H_48_ClO_4_ 507.3247. All spectroscopic data (NMR and MS) were in agreement with the literature.

#### 4.1.2. Preparation of Chloromethylpyrazine

Firstly, 2-methylpyrazine (10.63 mmol), NCS (1.42 g, 10.63 mmol), and BPO (0.26 g, 1.06 mmol) were added to a solution of CCl_4_ (40 mL), and the mixture was stirred for 2 h at room temperature under light conditions with nitrogen protection. The mixture was heated at reflux temperature for 8 h, cooled down, and then stirred in an ice bath for 1 h, before being filtered by suction, washed with 10 mL of carbon tetrachloride, and distilled under reduced pressure to give an irritating yellow oily liquid. The product was left untreated. Other methyl pyrazines included 2,3-dimethylpyrazine, 2,5-dimethylpyrazine, 2,6-dimethylpyrazine, 2,3,5-trimethylpyrazine, and 2,3,5,6-tetramethyl pyrazine, carried out as 2-methylpyrazine [24,25].

#### 4.1.3. Preparation of Pyrazinic Acid

Powdered potassium permanganate (3.3 g, 20.90 mmol) was dissolved in water (50 mL), then dropped into the aqueous solution of methyl pyrazine within 20 min. The mixture was stirred for 2 h at 75 °C. The reaction was monitored with thin-layer chromatography (TLC) until the end of the reaction. The mixture was cooled down, filtered, and washed with 50 mL of water; then, the filtrate was adjusted to pH 1.5 with nitric acid, slowly warmed to 50 °C, and maintained for 10 min, before being cooled down, extracted with 3 × 20 mL of ethyl acetate, and dried over anhydrous sodium sulfate. The product was left untreated. Other methyl pyrazines included 2,3-dimethylpyrazine, 2,6-dimethylpyrazine, 2,3,5-trimethylpyrazine and 2,3,5,6-tetramethyl pyrazine, carried out as 2,6-dimethylpyrazine [35,36].

#### 4.1.4. Preparation of Compound **7**

**He** (1.246 g, 2.64 mmol) and K_2_CO_3_ (728 mg, 5.28 mmol) were added to a solution of 2-bromoethanol (1.649 g, 13.20 mmol) in DMF (20 mL), and the mixture was stirred for 4 h at 85 °C. The reaction mixture was then diluted with ethyl acetate (30 mL), washed with water and brine successively, dried over anhydrous sodium sulfate, and purified by column chromatography to yield the white powder.

2-Hydroxyethyl (4aS,6aS,6bR,9R,10S,12aR)-10-hydroxy-9-(hydroxymethyl)-2,2,6a,6b,9,12a-hexamethyl-1,3,4,5,6,6a,6b,7,8,8a,9,10,11,12,12a,12b,13,14b-octadecahydropicene-4a(2H)-carboxylate (compound **7**): yield 95% (after chromatograph with DCM/MeOH 1–2%) as a white powder; m.p. 246.6 °C. ^1^H NMR (400 MHz, CDCl_3_) δ 5.28 (s, 1H, H-12), 4.05 (t, *J* = 5.9 Hz, 2H, H-31), 3.72 (d, *J* = 10.2 Hz, 1H, H-23a), 3.63 (t, *J* = 7.7 Hz, 1H, H-3), 3.56 (t, *J* = 6.3 Hz, 2H, H-32), 3.43 (d, *J* = 9.9 Hz, 1H, H-23a), 2.90–2.82 (m, 1H, H-18), 1.12 (s, 3H, –CH_3_), 0.96–0.88 (m, 12H, 4 × –CH_3_), 0.73 (s, 3H, –CH_3_). ^13^C NMR (100 MHz, CDCl_3_) δ 178.2, 144.1, 122.3, 76.8, 72.1, 66.1, 61.4, 49.8, 47.6, 46.9, 45.8, 41.8, 41.8, 41.5, 39.3, 38.1, 36.9, 33.8, 33.1, 32.5, 32.5, 30.7, 27.6, 26.7, 25.9, 23.6, 23.4, 23.0, 18.5, 17.1, 15.7, 11.4. HRMS (ESI) *m/z*: [M−H_2_O]^+^ 499.3790, calculated for C_32_H_50_O_4_ 499.3782.

#### 4.1.5. General Procedure for the Synthesis of Compounds **1**–**6**

**He** (282.2 mg, 0.6 mmol), chloromethyl pyrazine (153.6 mg, about 0.6 mmol, prepared as detailed in Section 4.1.2), and K_2_CO_3_ (248.4 mg, 1.8 mmol) were added to DMF (20 mL), and the mixture was stirred for 4 h at 85 °C. The reaction mixture was then diluted with ethyl acetate (20 mL), washed with water and brine, dried over anhydrous sodium sulfate, and purified by column chromatography to yield the white powders.

##### Synthesis of Compound **1**

Pyrazin-2-ylmethyl(4aS,6aS,6bR,9R,10S,12aR)-10-hydroxy-9-(hydroxymethyl)-2,2,6a,6b,9,12a-hexamethyl-1,3,4,5,6,6a,6b,7,8,8a,9,10,11,12,12a,12b,13,14b-octadecahydropicene-4a(2H)-carboxylate (compound **1**): yield 40% (after chromatograph with DCM/MeOH, 1–1.5%) as a white powder; m.p. 146.8 °C. ^1^H NMR (400 MHz, CDCl_3_) δ 8.68 (s, 1H, –N=C–H), 8.53 (d, *J* = 9.0 Hz, 2H, 2 × –N=C–H), 5.31 (s, 1H, H-12), 5.22 (q, *J* = 13.9 Hz, 2H, H-31), 3.71 (d, *J* = 10.3 Hz, 1H, H-23a), 3.65–3.59 (m, 1H, H-3), 3.41 (d, *J* = 10.3 Hz, 1H, H-23b), 2.91 (m, 1H, H-18), 1.12 (s, 3H, –CH_3_), 0.93–0.89 (m, 9H, 3 × –CH_3_), 0.87 (s, 3H, –CH_3_), 0.59 (s, 3H, –CH_3_). ^13^C NMR (100 MHz, CDCl_3_) δ 177.3, 152.2, 144.2, 144.0, 143.9, 143.6, 122.8, 77.0, 72.2, 64.8, 49.9, 47.7, 47.1, 45.9, 41.9, 41.9, 41.5, 39.4, 38.2, 37.0, 33.9, 33.2, 32.6, 32.6, 30.8, 27.8, 26.9, 26.0, 23.8, 23.5, 23.2, 18.6, 17.0, 15.8, 11.5. HRMS (ESI) *m/z*: [M + H]^+^ 565.4003, calculated for C_35_H_53_N_2_O_4_ 565.4000.

##### Synthesis of Compound **2**

(6-Methylpyrazin-2-yl)methyl(4aS,6aS,6bR,9R,10S,12aR)-10-hydroxy-9-(hydroxymethyl)-2,2,6a,6b,9,12a-hexamethyl-1,3,4,5,6,6a,6b,7,8,8a,9,10,11,12,12a,12b,13,14b-octadecahydropicene-4a(2H)-carboxylate (compound **2**): yield 35% (after chromatograph with DCM/MeOH, 1–1.5%) as a white powder; m.p. 107.0 °C. ^1^H NMR (400 MHz, CDCl_3_) δ 8.47 (s, 1H, –N=C–H), 8.39 (s, 1H, –N=C–H), 5.30 (s, 1H, H-12), 5.18 (q, *J* = 13.8 Hz, 2H, H-31), 3.71 (d, *J* = 10.3 Hz, 1H, H-23a), 3.62 (t, *J* = 7.9 Hz, 1H, H-3), 3.41 (d, *J* = 10.3 Hz, 1H, H-23b), 2.91 (m, 1H, H-18), 2.56 (s, 3H, –CH_3_), 1.12 (s, 3H, –CH_3_), 0.93–0.90 (m, 9H, 3 × –CH_3_), 0.88 (s, 3H, –CH_3_), 0.58 (s, 3H, –CH_3_). ^13^C NMR (100 MHz, CDCl_3_) δ 177.3, 153.4, 150.9, 143.7, 143.6, 140.4, 122.7, 77.0, 72.3, 64.9, 49.9, 47.7, 47.0, 46.0, 41.9, 41.8, 41.5, 39.4, 38.2, 37.0, 33.9, 33.2, 32.6, 32.6, 30.8, 27.8, 27.1, 26.9, 26.1, 23.8, 23.5, 23.2, 21.6, 18.6, 17.0, 15.8, 11.5. HRMS (ESI) *m/z*: [M + H]^+^ 579.4142, calculated for C_36_H_55_N_2_O_4_ 579.4156.

##### Synthesis of Compound **3**

(5-Methylpyrazin-2-yl)methyl(4aS,6aS,6bR,9R,10S,12aR)-10-hydroxy-9-(hydroxymethyl)-2,2,6a,6b,9,12a-hexamethyl-1,3,4,5,6,6a,6b,7,8,8a,9,10,11,12,12a,12b,13,14b-octadecahydropicene-4a(2H)-carboxylate (compound **3**): yield 60% (after chromatograph with DCM/MeOH, 1–1.5%) as a white powder; m.p. 170.3 °C. ^1^H NMR (400 MHz, CDCl_3_) δ 8.45 (m, 2H, 2 × –N=C–H), 5.30 (d, *J* = 2.7 Hz, 1H, H-12), 3.71 (d, *J* = 10.3 Hz, 1H, H-23a), 3.41 (d, *J* = 10.3 Hz, 1H, H-23b), 2.90 (m, 1H, H-18), 2.57 (d, *J* = 6.7 Hz, 3H, –CH_3_), 1.12 (s, 3H, –CH_3_), 0.91 (d, *J* = 6.6 Hz, 9H, 3 × –CH_3_), 0.88 (s, 3H, –CH_3_), 0.57 (s, 3H, –CH_3_). ^13^C NMR (100 MHz, CDCl_3_) δ 177.4, 153.1, 148.8, 143.9, 143.7, 142.7, 122.7, 77.0, 72.3, 64.7, 49.9, 47.7, 47.0, 45.9, 41.9, 41.8, 41.5, 39.4, 38.2, 37.0, 33.9, 33.2, 32.6, 32.6, 30.8, 27.8, 26.9, 26.1, 23.8, 23.5, 23.2, 21.4, 18.6, 17.0, 15.8, 11.5. HRMS (ESI) *m/z*: [M + H]^+^ 579.4142, calculated for C_36_H_55_N_2_O_4_ 579.4156.

##### Synthesis of Compound **4**

(3-Methylpyrazin-2-yl)methyl(4aS,6aS,6bR,9R,10S,12aR)-10-hydroxy-9-(hydroxymethyl)-2,2,6a,6b,9,12a-hexamethyl-1,3,4,5,6,6a,6b,7,8,8a,9,10,11,12,12a,12b,13,14b-octadecahydropicene-4a(2H)-carboxylate (compound **4**): yield 36% (after chromatograph with DCM/MeOH, 1–1.5%) as a white powder; m.p. 170.4 °C. ^1^H NMR (400 MHz, CDCl_3_) δ 8.41 (s, 1H, –N=C–H), 8.38 (s, 1H, –N=C–H), 5.22 (m, 3H, H-12, H-31a, H-32b), 3.71 (d, *J* = 10.3 Hz, 1H, H-23a), 3.64–3.59 (m, 1H, H-3), 3.41 (d, *J* = 10.3 Hz, 1H, H-23b), 2.85 (m, 1H, H-18), 2.63 (s, 3H, –CH_3_), 1.10 (s, 3H, –CH_3_), 0.93–0.88 (m, 12H, 4 × –CH_3_), 0.57 (s, 3H, –CH_3_). ^13^C NMR (100 MHz, CDCl_3_) δ 177.4, 153.1, 148.9, 143.9, 143.7, 142.7, 122.7, 77.0, 72.3, 64.7, 49.9, 47.7, 47.0, 45.93, 41.9, 41.8, 41.5, 39.4, 38.2, 37.0, 33.9, 33.2, 32.6, 32.6, 30.8, 27.8, 26.9, 26.1, 23.8, 23.5, 23.2, 21.4, 18.6, 17.0, 15.8, 11.5. HRMS (ESI) *m/z*: [M + H]^+^ 579.4145, calculated for C_36_H_55_N_2_O_4_ 579.4156.

##### Synthesis of Compound **5**

(5,6-Dimethylpyrazin-2-yl)methyl(4aS,6aS,6bR,9R,10S,12aR)-10-hydroxy-9-(hydroxymethyl)-2,2,6a,6b,9,12a-hexamethyl-1,3,4,5,6,6a,6b,7,8,8a,9,10,11,12,12a,12b,13,14b-octadecahydropicene-4a(2H)-carboxylate (compound **5**): yield approximately 40% (after chromatograph with DCM/MeOH, 1–1.5%) as a white solid; m.p. > 200 °C. ^1^H NMR (400 MHz, CDCl_3_) δ 8.24 (d, *J* = 13.0 Hz, 1H, –N=C–H), 5.22–5.09 (m, 3H, H-12, H-31), 3.67 (d, *J* = 10.1 Hz, 1H, H-23a), 3.62–3.57 (m, 1H, H-3), 3.37 (d, *J* = 10.1 Hz, 1H, H-23b), 2.83 (m, 1H, H-18), 2.59–2.49 (m, 6H, 2 × –CH_3_), 1.07 (s, 3H, –CH_3_), 0.90–0.85 (m, 12H, 4 × –CH3), 0.52 (d, *J* = 5.4 Hz, 3H, –CH3). ^13^C NMR (100 MHz, CDCl_3_) δ 177.3, 152.4, 149.9, 146.3, 143.5, 141.2, 122.6, 76.8, 72.0, 65.0, 49.9, 47.6, 47.0, 45.9, 41.8, 41.7, 41.4, 39.3, 38.2, 37.0, 33.9, 33.1, 32.6, 32.5, 30.8, 27.7, 26.6, 26.0, 23.7, 23.4, 23.1, 21.4, 21.2, 18.5, 16.9, 15.7, 11.6. HRMS (ESI) *m/z*: [M + H]^+^ 593.4295, calculated for C_37_H_57_N_2_O_4_ 593.4313.

##### Synthesis of Compound **6**

(3,5,6-Trimethylpyrazin-2-yl)methyl(4aS,6aS,6bR,9R,10S,12aR)-10-hydroxy-9-(hydroxymethyl)-2,2,6a,6b,9,12a-hexamethyl-1,3,4,5,6,6a,6b,7,8,8a,9,10,11,12,12a,12b,13,14b-octadecahydropicene-4a(2H)-carboxylate (compound **6**): yield 90% (after chromatograph with DCM/MeOH, 1–1.5%) as a white powder; m.p. 127.8 °C. ^1^H NMR (400 MHz, CDCl_3_) δ 5.24–5.08 (m, 3H, H-12, H-31a, H-31b), 3.72 (d, *J* = 10.2 Hz, 1H, H-23a), 3.62 (t, *J* = 7.8 Hz, 1H, H-3), 3.41 (d, *J* = 10.3 Hz, 1H, H-23b), 2.85 (m, 1H, H-18), 2.55–2.48 (m, 9H, 3 × –CH_3_), 1.09 (s, 3H, –CH_3_), 0.93–0.88 (m, 12H, 4 × –CH_3_), 0.53 (s, 3H, –CH_3_). ^13^C NMR (100 MHz, CDCl_3_) δ 177.3, 151.1, 149.3, 148.9, 145.5, 143.7, 122.5, 77.0, 72.3, 65.0, 50.0, 47.7, 47.0, 46.0, 41.9, 41.8, 41.4, 39.4, 38.2, 37.0, 34.0, 33.2, 32.6, 32.5, 30.8, 27.7, 26.9, 26.0, 23.8, 23.5, 23.2, 21.8, 21.5, 20.7, 18.6, 17.0, 15.8, 11.6. HRMS (ESI) *m/z*: [M + H]^+^ 607.4459, calculated for C_38_H_59_N_2_O_4_ 607.4469.

#### 4.1.6. General Procedure for the Synthesis of Compounds **8**–**13**

Pyrazinic acid (0.27 mmol, prepared as detailed in Section 4.1.3), EDCI (0.35 mmol), and DMAP (0.027 mmol) were added to dried dichloromethane (20 mL). Then, compound **7** (140 mg, 0.27 mmol) dissolved in dichloromethane (20 mL) was dropped into the aqueous solution of methyl pyrazine within 20 min, and the mixture was stirred for 12 h at room temperature. The reaction mixture was then diluted with dichloromethane (20 mL), washed with water and brine, dried over anhydrous sodium sulfate, concentrated, and purified by column chromatography to yield the white powders.

##### Synthesis of Compound **8**

2-(((4aS,6aS,6bR,9R,10S,12aR)-10-Hydroxy-9-(hydroxymethyl)-2,2,6a,6b,9,12a-hexamethyl-1,2,3,4,4a,5,6,6a,6b,7,8,8a,9,10,11,12,12a,12b,13,14b-icosahydropicene-4a-carbonyl)oxy)ethylpyrazine-2-carboxylate (compound **8**): yield 20% (after chromatograph with DCM/MeOH, 1–2%) as a white powder; m.p. 154.7 °C. ^1^H NMR (400 MHz, CDCl_3_) δ 9.29 (d, *J* = 9.6 Hz, 1H, –N=C–H), 8.80–8.70 (m, 2H, 2 × –N=C–H), 5.30 (s, 1H, H-12), 3.62 (t, *J* = 7.5 Hz, 1H, H-3), 2.88 (m, 1H, H-18), 0.98 (s, 3H, –CH_3_), 0.91 (d, *J* = 10.4 Hz, 9H, 3 × –CH_3_), 0.77 (s, 3H, –CH_3_). ^13^C NMR (100 MHz, CDCl_3_) δ 178.3, 163.9, 148.0, 146.2, 144.7, 144.3, 143.4, 122.4, 77.4, 71.9, 66.2, 61.6, 49.6, 48.0, 47.1, 45.9, 42.3, 41.9, 41.7, 39.5, 38.3, 37.0, 34.0, 33.2, 32.6, 30.8, 27.7, 26.4, 26.1, 25.8, 23.7, 23.5, 23.1, 18.8, 17.2, 15.7, 12.3. HRMS (ESI) m/z: [M + H]^+^ 623.4045, calculated for C_37_H_55_N_2_O_6_ 623.4055.

##### Synthesis of Compound **9**

2-(((4aS,6aS,6bR,9R,10S,12aR)-10-Hydroxy-9-(hydroxymethyl)-2,2,6a,6b,9,12a-hexamethyl-1,2,3,4,4a,5,6,6a,6b,7,8,8a,9,10,11,12,12a,12b,13,14b-icosahydropicene-4a-carbonyl)oxy)ethyl-6-methylpyrazine-2-carboxylate (compound **9**): yield 35% (after chromatograph with DCM/MeOH, 1–2%) as a white powder; m.p. 122.9 °C. ^1^H NMR (400 MHz, CDCl_3_) δ 9.06 (d, *J* = 21.2 Hz, 1H, –N=C–H), 8.63 (d, *J* = 5.4 Hz, 1H, –N=C–H), 5.31 (s, 1H, H-12), 4.25–3.99 (m, 2H), 2.87 (m, 1H, H-18), 2.67 (d, *J* = 9.0 Hz, 3H, –CH_3_), 1.11 (s, 3H, –CH_3_), 0.92 (m, 12H, 4 × –CH_3_), 0.77 (s, 3H, –CH_3_). ^13^C NMR (100 MHz, CDCl_3_) δ 178.3, 164.2, 154.4, 148.1 144.2, 143.0, 142.2, 122.4, 74.1, 72.0, 66.2, 61.6, 49.7, 48.0, 47.1, 45.9, 42.3, 41.9, 41.7, 39.4, 38.3, 37.0, 33.9, 33.2, 32.6, 32.7, 30.8, 27.7, 26.3, 26.1, 25.9, 23.5, 23.1, 21.9, 18.8, 17.2, 15.7, 12.3. HRMS (ESI) *m/z*: [M +H]^+^ 637.4219, calculated for C_38_H_57_N_2_O_6_ 637.4211.

##### Synthesis of Compound **10**

2-(((4aS,6aS,6bR,9R,10S,12aR)-10-Hydroxy-9-(hydroxymethyl)-2,2,6a,6b,9,12a-hexamethyl-1,2,3,4,4a,5,6,6a,6b,7,8,8a,9,10,11,12,12a,12b,13,14b-icosahydropicene-4a-carbonyl)oxy)ethyl-5-methylpyrazine-2-carboxylate (compound **10**): yield 55% (after chromatograph with DCM/MeOH, 1–2%) as a white powder; m.p. 192.6 °C. ^1^H NMR (400 MHz, CDCl_3_) δ 9.17 (s, 1H, –N=C–H), 8.59 (s, 1H, –N=C–H), 5.22 (s, 1H, H-12), 4.68–4.59 (m, 2H, H-31), 4.46–4.31 (m, 2H, H-32), 3.70 (d, *J* = 10.3 Hz, 1H, H-23a), 3.61 (t, *J* = 7.8 Hz, 1H, H-3), 3.40 (d, *J* = 10.3 Hz, 1H, H-23b), 2.84 (m, 1H, H-18), 2.67 (s, 3H, –CH_3_), 1.08 (s, 3H), 0.87 (s, 12H, 4 × –CH_3_), 0.65 (s, 3H, –CH_3_). ^13^C NMR (100 MHz, CDCl_3_) δ 177.6, 163.9, 158.1, 145.5, 144.6, 143.6, 140.5, 122.6, 77.0, 72.2, 63.6, 62.0, 49.9, 47.7, 46.9, 45.9, 41.9, 41.8, 41.3, 39.4, 38.2, 37.0, 33.9, 33.2, 32.5, 32.5, 30.8, 27.7, 26.9, 26.0, 23.7, 23.4, 23.0, 22.1, 18.6, 17.0, 15.7, 11.5. HRMS (ESI) *m/z*: [M + H]^+^ 637.4208, calculated for C_38_H_57_N_2_O_6_ 637.4211.

##### Synthesis of Compound **11**

2-(((4aS,6aS,6bR,9R,10S,12aR)-10-Hydroxy-9-(hydroxymethyl)-2,2,6a,6b,9,12a-hexamethyl-1,2,3,4,4a,5,6,6a,6b,7,8,8a,9,10,11,12,12a,12b,13,14b-icosahydropicene-4a-carbonyl)oxy)ethyl-3-methylpyrazine-2-carboxylate (compound **11**): yield 35% (after chromatograph with DCM/MeOH, 1–2%) as a white powder; m.p. 146.8 °C. ^1^H NMR (400 MHz, CDCl_3_) δ 8.62 (s, 1H, –N=C–H), 8.48 (s, 1H, –N=C–H), 5.31 (s, 1H, H-12), 4.59 (d, *J* = 10.8 Hz, 1H, H-23a), 4.04 (d, *J* = 10.8 Hz, 1H, H-23b), 3.67–3.58 (m, 1H, H-3), 2.87 (s, 4H, H-18, –CH_3_), 1.13 (s, 3H, –CH_3_), 0.94 (m, 12H, 4 × –CH_3_), 0.77 (s, 3H, –CH_3_). ^13^C NMR (100 MHz, CDCl_3_) δ 178.3, 165.0, 155.7, 146.5, 144.2, 142.5, 141.7, 122.4, 74.8, 73.7, 66.2, 61.6, 50.0, 47.9, 47.1, 45.9, 42.1, 42.0, 41.7, 39.4, 38.2, 37.0, 34.0, 33.2, 32.6, 32.6, 30.8, 27.8, 26.3, 25.9, 23.7, 23.5, 23.3, 23.2, 19.0, 17.2, 15.6, 12.36. HRMS (ESI) *m/z*: [M + H]^+^ 637.4222, calculated for C_38_H_57_N_2_O_6_ 637.4211.

##### Synthesis of Compound **12**

2-(((4aS,6aS,6bR,9R,10S,12aR)-10-Hydroxy-9-(hydroxymethyl)-2,2,6a,6b,9,12a-hexamethyl-1,2,3,4,4a,5,6,6a,6b,7,8,8a,9,10,11,12,12a,12b,13,14b-icosahydropicene-4a-carbonyl)oxy)ethyl-5,6-dimethylpyrazine-2-carboxylate (compound **12**): yield 25% (after chromatograph with DCM/MeOH, 1–2%) as a white powder; m.p. 146.2 °C. ^1^H NMR (400 MHz, CDCl_3_) δ 8.41 (d, *J* = 60.1 Hz, 1H, –N=C–H), 5.31 (s, 1H, H-12), 4.59 (d, *J* = 10.5 Hz, 1H, H-23a), 4.00 (d, *J* = 10.7 Hz, 1H, H-23b), 2.83 (m, 4H, H-18, –CH_3_), 2.58 (d, *J* = 6.8 Hz, 3H, –CH_3_), 1.13 (s, 3H, –CH_3_), 0.97–0.90 (m, 12H, 4 × –CH_3_), 0.76 (s, 3H, –CH_3_). ^13^C NMR (100 MHz, CDCl_3_) δ 178.3, 165.2, 154.8, 151.0, 146.5, 144.2, 141.5, 122.4, 75.0, 73.8, 66.2, 61.6, 50.2, 47.8, 47.1, 45.9, 42.1, 42.0, 41.6, 39.4, 38.2, 37.0, 33.9, 33.2, 32.6, 30.8,27.8, 26.2, 25.9, 23.7, 23.5, 23.2, 22.7, 22.0, 21.2, 19.0, 17.2, 15.6, 12.4. HRMS (ESI) *m/z*: [M + H]^+^ 651.4375, calculated for C_39_H_59_N_2_O_6_ 651.4368.

##### Synthesis of Compound **13**

2-(((4aS,6aS,6bR,9R,10S,12aR)-10-Hydroxy-9-(hydroxymethyl)-2,2,6a,6b,9,12a-hexamethyl-1,2,3,4,4a,5,6,6a,6b,7,8,8a,9,10,11,12,12a,12b,13,14b-icosahydropicene-4a-carbonyl)oxy)ethyl-3,5,6-trimethylpyrazine-2-carboxylate (compound **13**): yield 90% (after chromatograph with DCM/MeOH, 1–2%) as a white powder; m.p. 150.6 °C. ^1^H NMR (400 MHz, CDCl_3_) δ 5.31 (s, 1H, H-12), 4.60 (d, *J* = 10.7 Hz, 1H, H-23a), 3.94 (d, *J* = 10.7 Hz, 1H, H-23b), 3.66–3.59 (m, 1H, H-3), 2.87 (m, 1H, H-18), 2.79 (s, 3H, –CH_3_), 2.57 (m, 9H, 3 × –CH_3_), 1.13 (s, 3H, –CH_3_), 0.99–0.89 (m, 15H, 5 × –CH_3_), 0.77 (s, 3H, –CH_3_). ^13^C NMR (100 MHz, CDCl_3_) δ 178.3, 165.3, 155.4, 152.2, 149.6, 144.2, 138.1, 122.4, 75.4, 74.6, 66.2, 61.6, 50.7, 47.8, 47.1, 45.9, 42.0, 42.0, 41.6, 39.4, 38.2, 36.98, 34.0, 33.20, 32.60, 30.8, 29.8, 27.8, 26.2, 26.0, 23.8, 23.5, 23.2, 22.7, 22.4, 22.3, 21.8, 19.1, 17.2, 15.5, 12.5. HRMS (ESI) *m/z*: [M + H]^+^ 665.4531, calculated for C_40_H_61_N_2_O_6_ 665.4524.

#### 4.1.7. General Procedure for the Synthesis of Compounds **14**–**19**

Pyrazinic acid (248 mg, about 2 mmol, prepared as detailed in Section 4.1.3), 4-chloro-1-butanol (2.4 mmol), EDCI (2.4 mmol), and DMAP (0.2 mmol) were added into dried dichloromethane (20 mL). The mixture was stirred for 12 h at room temperature, then diluted with dichloromethane (20 mL), washed with water and brine, dried over anhydrous sodium sulfate, concentrated and purified by column chromatography to yield the oily liquid. Next, **He** (236 mg, 0.5 mmol), K_2_CO_3_ (207 mg, 1.5 mmol), and the oily liquid were added to DMF (20 mL), and the mixture was stirred for 4 h at 85 °C. The reaction mixture was then diluted with ethyl acetate (20 mL), washed with water and brine, dried over anhydrous sodium sulfate, concentrated and purified by column chromatography to yield the white powders.

##### Synthesis of Compound **14**

4-(((4aS,6aS,6bR,9R,10S,12aR)-10-Gydroxy-9-(hydroxymethyl)-2,2,6a,6b,9,12a-hexamethyl-1,2,3,4,4a,5,6,6a,6b,7,8,8a,9,10,11,12,12a,12b,13,14b-icosahydropicene-4a-carbonyl)oxy)butyl-pyrazine-2-carboxylate (compound **14**): yield 30% (after chromatograph with DCM/MeOH, 1–2%) as a white powder; m.p. 127 °C. ^1^H NMR (400 MHz, CDCl_3_) δ 9.31 (s, 1H, –N=C–H), 8.77 (s, 1H, –N=C–H), 8.74 (s, 1H, –N=C–H), 5.27 (s, 1H, H-12), 4.48 (t, *J* = 6.3 Hz, 2H, H-34), 4.08 (t, *J* = 5.7 Hz, 2H, H-31), 3.72 (d, *J* = 10.3 Hz, 1H, H-23a), 3.62 (t, *J* = 7.5 Hz, 1H, H-3), 3.42 (d, *J* = 10.1 Hz, 1H, H-23b), 2.86 (m, 1H, H-18), 1.12 (s, 3H, –CH_3_), 0.93–0.88 (m, 12H, 4 × –CH_3_), 0.71 (s, 3H, –CH_3_). ^13^C NMR (100 MHz, CDCl3) δ 177.8, 164.0, 147.8, 146.4, 144.6, 143.9, 143.6, 122.5, 77.0, 72.3, 66.0, 63.7, 49.9, 47.7, 46.9, 46.0, 41.9, 41.9, 41.5, 39.5, 38.2, 37.0, 34.0, 33.2, 32.7, 32.6, 30.8, 27.8, 26.9, 26.0, 25.7, 25.4, 23.8, 23, 23.1, 18.6, 17.2, 15.8, 11.5. HRMS (ESI) *m/z*: [M + H]^+^ 651.4375, calculated for C_39_H_59_N_2_O_6_ 651.4368.

##### Synthesis of Compound **15**

4-(((4aS,6aS,6bR,9R,10S,12aR)-10-Hydroxy-9-(hydroxymethyl)-2,2,6a,6b,9,12a-hexamethyl-1,2,3,4,4a,5,6,6a,6b,7,8,8a,9,10,11,12,12a,12b,13,14b-icosahydropicene-4a-carbonyl)oxy)butyl-6-methylpyrazine-2-carboxylate (compound **15**): yield 35% (after chromatograph with DCM/MeOH, 1–2%) as a white powder; m.p. 128 °C. ^1^H NMR (400 MHz, CDCl_3_) δ 9.09 (s, 1H, –N=C–H), 8.63 (s, 1H, –N=C–H), 5.27 (s, 1H, H-12), 4.47 (t, *J* = 6.3 Hz, 2H, H-34), 4.08 (t, *J* = 5.8 Hz, 2H, H-31), 3.71 (d, *J* = 10.2 Hz, 1H, H-23a), 3.62 (t, *J* = 7.5 Hz, 1H, H-3), 3.42 (d, *J* = 10.2 Hz, 1H, H-23b), 2.86 (m, 1H, H-18), 2.68 (s, 3H, –CH_3_), 1.11 (s, 3H, –CH_3_), 0.92–0.87 (m, 12H, 4 × –CH_3_), 0.71 (s, 3H, –CH_3_). ^13^C NMR (100 MHz, CDCl_3_) δ 177.8, 164.3, 154.4, 147.8, 143.9, 143.2, 142.6, 122.5, 77.0, 72.3, 65.8, 63.7, 49.9, 47.7, 46.9, 46.0, 41.9, 41.9, 41.5, 39.5, 38.2, 37.0, 34.0, 33.2, 32.7, 32.6, 30.8, 27.8, 26.9, 26.0, 25.7, 25.4, 23.8, 23.5, 23.1, 21.9, 18.6, 17.2, 15.8, 11.5. HRMS (ESI) *m/z*: [M + H]^+^ 665.4527, calculated for C_40_H_61_N_2_O_6_ 665.4524.

##### Synthesis of Compound **16**

4-(((4aS,6aS,6bR,9R,10S,12aR)-10-Hydroxy-9-(hydroxymethyl)-2,2,6a,6b,9,12a-hexamethyl-1,2,3,4,4a,5,6,6a,6b,7,8,8a,9,10,11,12,12a,12b,13,14b-icosahydropicene-4a-carbonyl)oxy)butyl-5-methylpyrazine-2-carboxylate (compound **16**): yield 26% (after chromatograph with DCM/MeOH, 1–2%) as a white powder; m.p. 166.1 °C. ^1^H NMR (400 MHz, CDCl_3_) δ 9.17 (s, 1H, –N=C–H), 8.58 (s, 1H, –N=C–H), 5.27 (s, 1H, H-12), 4.46 (t, *J* = 6.4 Hz, 2H, H-34), 4.08 (t, *J* = 5.9 Hz, 2H, H-31), 3.71 (d, *J* = 10.2 Hz, 1H, H-23a), 3.62 (t, *J* = 7.5 Hz, 1H, H-3), 3.42 (d, *J* = 10.2 Hz, 1H, H-23b), 2.85 (m, 1H, H-18), 2.67 (s, 3H, –CH_3_), 1.11 (s, 3H, –CH_3_), 0.92–0.88 (m, 12H, 4 × –CH_3_), 0.71 (s, 3H, –CH_3_). ^13^C NMR (100 MHz, CDCl_3_) δ 177.8, 164.3, 158.0, 145.5, 144.5, 143.9, 140.8, 122.5, 77.0, 72.3, 65.7, 63.7, 49.9, 47.7, 46.9, 46.0, 41.9, 41.9, 41.5, 39.5, 38.2, 37.0, 34.0, 33.2, 32.7, 32.6, 30.8, 27.8, 26.9, 26.0, 25.7, 25.4, 23.8, 23.5, 23.1, 22.1, 18.6, 17.2, 15.8, 11.5. HRMS (ESI) *m/z*: [M + H]^+^ 665.4538, calculated for C_40_H_61_N_2_O_6_ 665.4524.

##### Synthesis of Compound **17**

4-(((4aS,6aS,6bR,9R,10S,12aR)-10-Hydroxy-9-(hydroxymethyl)-2,2,6a,6b,9,12a-hexamethyl-1,2,3,4,4a,5,6,6a,6b,7,8,8a,9,10,11,12,12a,12b,13,14b-icosahydropicene-4a-carbonyl)oxy)butyl-3-methylpyrazine-2-carboxylate (compound **17**): yield 31% (after chromatograph with DCM/MeOH, 1–2%) as a white powder; m.p. 145.2 °C. ^1^H NMR (400 MHz, CDCl_3_) δ 8.61 (s, 1H, –N=C–H), 8.52 (s, 1H, –N=C–H), 5.29 (s, 1H, H-12), 4.44 (t, *J* = 6.5 Hz, 2H, H-34), 4.07 (t, *J* = 6.0 Hz, 2H, H-31), 3.71 (d, *J* = 10.1 Hz, 1H, H-23a), 3.62 (t, *J* = 7.6 Hz, 1H, H-3), 3.42 (d, *J* = 10.2 Hz, 1H, H-23b), 2.84 (s, 3H, –CH_3_), 1.11 (s, 3H, –CH_3_), 0.92–0.87 (m, 12H, 4 × –CH_3_), 0.71 (s, 3H, –CH_3_). ^13^C NMR (100 MHz, CDCl3) δ 177.8, 165.4, 155.3, 146.0, 143.9, 143.3, 141.6, 122.5, 77.0, 72.3, 65.8, 63.7, 49.9, 47.71, 46.9, 46.0, 41.9, 41.9, 41.5, 39.5, 38.2, 37.0, 34.0, 33.2, 32.7, 32.6, 30.8, 27.8, 26.9, 26.0, 25.7, 25.4, 23.8, 23.5, 23.4, 23.1, 18.6, 17.2, 15.8, 11.5. HRMS (ESI) *m/z*: [M + H]^+^ 665.4542, calculated for C_40_H_61_N_2_O_6_ 665.4524.

##### Synthesis of Compound **18**

4-(((4aS,6aS,6bR,9R,10S,12aR)-10-Hydroxy-9-(hydroxymethyl)-2,2,6a,6b,9,12a-hexamethyl-1,2,3,4,4a,5,6,6a,6b,7,8,8a,9,10,11,12,12a,12b,13,14b-icosahydropicene-4a-carbonyl)oxy)butyl-5,6-dimethylpyrazine-2-carboxylate (compound **18**): yield 27% (after chromatograph with DCM/MeOH, 1–2%) as a white powder; m.p. 134.7 °C. ^1^H NMR (400 MHz, CDCl_3_) δ 8.43 (d, J = 32.0 Hz, 1H, –N=C–H), 5.27 (s, 1H, H-12), 4.43 (d, *J* = 6.2 Hz, 2H, H-34), 4.07 (d, *J* = 3.0 Hz, 2H, H-34), 3.72 (d, *J* = 10.1 Hz, 1H, H-23a), 3.62 (t, *J* = 7.6 Hz, 1H, H-3), 3.42 (d, *J* = 10.2 Hz, 1H, H-23b), 2.88–2.59 (m, 7H, H-18, 2 × –CH_3_), 1.11 (s, 3H, –CH_3_), 0.93–0.87 (m, 12H, 4 × –CH_3_), 0.71 (s, 3H, –CH_3_). ^13^C NMR (100 MHz, CDCl_3_) δ 177.8, 155.9, 154.5, 145.8, 143.9, 142.8, 141.4, 122.5, 77.0, 72.3, 65.6, 63.8, 49.9, 47.7, 46.9, 46.0, 41.9, 41.9, 41.5, 39.5, 38.2, 37.0, 34.0, 33.2, 32.6, 30.8, 27.8, 26.9, 26.0, 25.7, 25.4, 23.8, 23.5, 23.1, 22.6, 21.9, 21.3, 18.6, 17.2, 15.8, 11.5. HRMS (ESI) *m/z*: [M + H]^+^ 679.4682, calculated for C_41_H_63_N_2_O_6_ 679.4681.

##### Synthesis of Compound **19**

4-(((4aS,6aS,6bR,9R,10S,12aR)-10-Hydroxy-9-(hydroxymethyl)-2,2,6a,6b,9,12a-hexamethyl-1,2,3,4,4a,5,6,6a,6b,7,8,8a,9,10,11,12,12a,12b,13,14b-icosahydropicene-4a-carbonyl)oxy)butyl-3,5,6-trimethylpyrazine-2-carboxylate (compound **19**): yield 90.4% (after chromatograph with DCM/MeOH, 1–2%) as a white powder; m.p. 110.1 °C. ^1^H NMR (400 MHz, CDCl_3_) δ 5.27 (s, 1H, H-12), 4.42 (t, *J* = 6.3 Hz, 2H, H-34), 4.08 (t, *J* = 5.7 Hz, 2H, H-31), 3.72 (d, *J* = 10.3 Hz, 1H, H-23a), 3.62 (t, *J* = 7.5 Hz, 1H, H-3), 3.42 (d, *J* = 10.2 Hz, 1H, H-23b), 2.86 (m, 1H, H-18), 2.74 (s, 3H, –CH_3_), 2.57 (s, 6H, 2 × –CH_3_), 1.12 (s, 3H, –CH_3_), 0.92–0.87 (m, 12H, 4 × –CH_3_), 0.71 (s, 3H, –CH_3_). ^13^C NMR (100 MHz, CDCl_3_) δ 177.8, 166.1, 154.5, 151.1, 149.5, 143.9, 139.8, 122.5, 77.0, 72.3, 65.5, 63.8, 49.9, 47.70, 46.8, 46.0, 41.9, 41.8, 41.5, 39.4, 38.2, 37.0, 34.0, 33.2, 32.6, 32.6, 30.8, 27.8, 26.9, 26.0, 25.7, 25.5, 23.7, 23.5, 23.1, 22.7, 22.3, 21.7, 18.6, 17.2, 15.8, 11.5. HRMS (ESI) *m/z*: [M + H]^+^ 693.4849, calculated for C_42_H_65_N_2_O_6_ 693.4837.

#### 4.1.8. Procedure for the Synthesis of Compound **20**

**He** (1.236 g, 2.64 mmol) and K_2_CO_3_ (730 mg, 5.28 mmol) were added to a solution of 4-chlorobutanol (1.43 g, 13.20 mmol) in DMF (20 mL), and the mixture was stirred for 4h at 85 °C. The reaction mixture was then diluted with ethyl acetate (30 mL), washed with water and brine successively, dried over anhydrous sodium sulfate, concentrated, and purified by column chromatography to yield the white powder. Yield 54% (after chromatograph with DCM/MeOH, 1–2%) as a white powder, m.p. 170.2 °C. ^1^H NMR (400 MHz, CDCl_3_) δ 5.28 (s, 1H, H-12), 3.72 (d, *J* = 10.2 Hz, 1H, H-23a), 3.63 (t, *J* = 7.6 Hz, 1H, H-3), 3.42 (d, *J* = 10.2 Hz, 1H, H-23b), 2.89–2.80 (m, 1H, H-18), 1.12 (s, 3H, –CH_3_), 0.95 (s, 3H, –CH_3_), 0.93–0.88 (m, 9H, 3 × –CH_3_), 0.73 (d, *J* = 8.5 Hz, 3H, –CH_3_). ^13^C NMR (100 MHz, CDCl_3_) δ 177.7, 143.8, 122.4, 76.9, 72.2, 63.3, 49.8, 47.6, 46.7, 45.9, 44.5, 41.8, 41.7, 41.3, 39.3, 38.1, 36.9, 33.9, 33.1, 32.5, 32.5, 30.7, 29.4, 27.6, 26.8, 26.1, 25.9, 23.6, 23.4, 23.0, 18.5, 17.1, 15.7, 11.4. HRMS (ESI) *m/z*: [M–H_2_O + H]^+^ 545.3752, calculated for C_34_H_54_ClO_3_ 545.3756.

#### 4.1.9. General Procedure for the Synthesis of Compounds **21**–**26**

Pyrazinic acid (about 0.27 mmol, prepared as detailed in Section 4.1.3), EDCI (0.35 mmol), and DMAP (0.027 mmol) were added to dried dichloromethane (20 mL); then, compound **20** (152 mg, 0.27 mmol) dissolved in dichloromethane (20 mL) was dropped into the aqueous solution of methyl pyrazine within 20 min, and the mixture was stirred for 12 h at room temperature. The reaction mixture was then diluted with dichloromethane (20 mL), washed with water and brine, dried over anhydrous sodium sulfate, concentrated, and purified by column chromatography to yield the white powders.

##### Synthesis of Compound **21**

((3S,4R,6aR,6bS,8aS,14bR)-8a-((4-Chlorobutoxy)carbonyl)-3-hydroxy-4,6a,6b,11,11,14b-hexamethyl-1,2,3,4,4a,5,6,6a,6b,7,8,8a,9,10,11,12,12a,14,14a,14b-icosahydropicen-4-yl)methyl-pyrazine-2-carboxylate (compound **21**): yield 31% (after chromatograph with DCM/MeOH, 1–2%) as a white powder; m.p. 117.1 °C. ^1^H NMR (400 MHz, CDCl_3_) δ 9.28 (d, *J* = 9.3 Hz, 1H, –N=C–H), 8.85–8.59 (m, 2H, 2 × –N=C–H), 5.28 (s, 1H, H-12), 2.86 (m, 1H, H-18), 1.09 (s, 3H, –CH_3_), 1.02–0.83 (m, 12H, 4 × –CH_3_), 0.75 (s, 3H, –CH_3_). ^13^C NMR (100 MHz, CDCl_3_) δ 177.8, 163.9, 148.0, 146.4, 146.2, 144.7, 143.9, 122.4, 74.0, 71.9, 63.5, 49.6, 48.0, 46.9, 45.9, 44.6, 42.3, 41.8, 41.5, 39.5, 38.3, 37.0, 34.0, 33.2, 32.6, 30.8, 29.5, 27.7, 26.4, 26.2, 26.1, 25.8, 23.7, 23.5, 23.1, 18.8, 17.2, 15.7, 12.3. HRMS (ESI) *m/z*: [M + H]^+^ 669.4045, calculated for C_39_H_58_ClN_2_O_5_ 669.4029.

##### Synthesis of Compound **22**

((3S,4R,6aR,6bS,8aS,14bR)-8a-((4-Chlorobutoxy)carbonyl)-3-hydroxy-4,6a,6b,11,11,14b-hexamethyl-1,2,3,4,4a,5,6,6a,6b,7,8,8a,9,10,11,12,12a,14,14a,14b-icosahydropicen-4-yl)methyl 6-methylpyrazine-2-carboxylate (compound **22**): yield 35% (after chromatograph with DCM/MeOH, 1–2%) as a white powder; m.p. 126.9 °C. ^1^H NMR (400 MHz, CDCl_3_) δ 9.07 (s, 1H, –N=C–H), 8.63 (s, 1H, –N=C–H), 5.27 (s, 1H, H-12), 2.84 (m, 1H, H-18), 2.64 (s, 3H, –CH_3_), 1.09 (s, 3H, –CH_3_), 0.96 (s, 3H, –CH_3_), 0.89 (m, 9H, 3 × –CH_3_), 0.74 (s, 3H, –CH_3_). ^13^C NMR (100 MHz, CDCl_3_) δ 177.7, 164.6, 154.4, 148.0, 143.8, 142.9, 142.2, 122.4, 74.0, 71.8, 63.4, 49.6, 47.9, 46.8, 45.9, 44.6, 42.2, 41.8, 41.5, 39.4, 38.3, 36.9, 34.0, 33.2, 32.7, 32.6, 30.8, 29.5, 27.7, 26.3, 26.2, 25.8, 23.7, 23.5, 23.1, 21.8, 18.8, 17.2, 15.6, 12.3. HRMS (ESI) *m/z*: [M + H]^+^ 683.4185, calculated for C_40_H_60_ClN_2_O_5_ 683.4185.

##### Synthesis of Compound **23**

((3S,4R,6aR,6bS,8aS,14bR)-8a-((4-Chlorobutoxy)carbonyl)-3-hydroxy-4,6a,6b,11,11,14b-hexamethyl-1,2,3,4,4a,5,6,6a,6b,7,8,8a,9,10,11,12,12a,14,14a,14b-icosahydropicen-4-yl)methyl 5-methylpyrazine-2-carboxylate (compound **23**): yield 40% (after chromatograph with DCM/MeOH, 1–2%) as a white powder; m.p. 175.8 °C. ^1^H NMR (400 MHz, CDCl_3_) δ 9.17 (s, 1H, –N=C–H), 8.56 (s, 1H, –N=C–H), 5.29 (s, 1H, H-12), 2.87 (m, 1H, H-18), 2.66 (d, *J* = 5.3 Hz, 3H, –CH_3_), 1.10 (s, 3H, –CH_3_), 0.98 (s, 3H, –CH_3_), 0.91 (m, 9H, 3 × –CH_3_), 0.75 (s, 3H, –CH_3_). ^13^C NMR (100 MHz, CDCl_3_) δ 177.8, 164.3, 158.2, 145.2, 144.6, 143.9, 140.5, 122.5, 74.1, 71.8, 63.5, 49.7, 48.0, 46.9, 46.0, 44.6, 42.3, 41.8, 41.5, 39.5, 38.3, 37.0, 34.0, 33.3, 32.7, 32.6, 30.8, 29.5, 27.7, 27.1, 26.3, 25.9, 23.7, 23.5, 23.1, 22.1, 18.8, 17.2, 15.7, 12.3. HRMS (ESI) *m/z*: [M + H]^+^ 683.4183, calculated for C_40_H_60_ClN_2_O_5_ 683.4185.

##### Synthesis of Compound **24**

((3S,4R,6aR,6bS,8aS,14bR)-8a-((4-Chlorobutoxy)carbonyl)-3-hydroxy-4,6a,6b,11,11,14b-hexamethyl-1,2,3,4,4a,5,6,6a,6b,7,8,8a,9,10,11,12,12a,14,14a,14b-icosahydropicen-4-yl)methyl 3-methylpyrazine-2-carboxylate (compound **24**): yield 36% (after chromatograph with DCM/MeOH, 1–2%) as a white powder; m.p. 106.1 °C. ^1^H NMR (400 MHz, CDCl_3_) δ 8.62 (s, 1H, –N=C–H), 8.49 (s, 1H, –N=C–H), 5.29 (s, 1H, H-12), 3.65–3.59 (m, 1H, H-3), 2.86 (m, 4H, H-18, –CH_3_), 1.12 (s, 3H, –CH_3_), 0.93 (m, 12H, 3 × –CH_3_), 0.75 (s, 3H, –CH_3_). ^13^C NMR (100 MHz, CDCl_3_) δ 177.8, 165.0, 155.7, 146.4, 143.9, 142.5, 141.7, 122.5, 74.9, 73.7, 63.5, 50.1, 47.9, 46.9, 46.0, 44.6, 42.1, 41.9, 41.5, 39.5, 38.2, 37.0, 34.0, 33.3, 32.6, 32.6, 30.8, 29.5, 27.8, 26.3, 26.3, 25.9, 23.7, 23.5, 23.3, 23.1, 19.0, 17.2, 15.6, 12.4. HRMS (ESI) *m/z*: [M + H]^+^ 683.4175, calculated for C_40_H_60_ClN_2_O_5_ 683.4185.

##### Synthesis of Compound **25**

((3S,4R,6aR,6bS,8aS,14bR)-8a-((4-Chlorobutoxy)carbonyl)-3-hydroxy-4,6a,6b,11,11,14b-hexamethyl-1,2,3,4,4a,5,6,6a,6b,7,8,8a,9,10,11,12,12a,14,14a,14b-icosahydropicen-4-yl)methyl 5,6-dimethylpyrazine-2-carboxylate (compound **25**): yield 36% (after chromatograph with DCM/MeOH, 1–2%) as a white powder; m.p. 123.6 °C. ^1^H NMR (400 MHz, CDCl_3_) δ 8.42 (d, *J* = 59.1 Hz, 1H, –N=C–H), 5.29 (s, 1H, H-12), 2.83 (d, *J* = 8.1 Hz, 3H, –CH_3_), 2.59 (d, *J* = 6.8 Hz, 3H, –CH_3_), 1.12 (s, 3H, –CH_3_), 0.99–0.89 (m, 12H, 4 × –CH_3_), 0.75 (s, 3H, –CH_3_). ^13^C NMR (100 MHz, CDCl_3_) δ 177.8, 165.2, 156.4, 154.8, 146.4, 143.9, 141.5, 122.5, 75.0, 73.9, 63.5, 50.3, 47.9, 46.9, 46.0, 44.6, 42.1, 41.9, 41.5, 39.4, 38.2, 37.0, 34.0, 33.2, 32.6, 30.8, 29.5, 27.8, 26.3, 25.9, 23.7, 23.5, 23.3, 23.1, 22.0, 21.2, 19.0, 17.3, 15.6, 12.4, 12.4. HRMS (ESI) *m/z*: [M + H]^+^ 697.4343, calculated for C_41_H_62_ClN_2_O_5_ 697.4342.

##### Synthesis of Compound **26**

((3S,4R,6aR,6bS,8aS,14bR)-8a-((4-Chlorobutoxy)carbonyl)-3-hydroxy-4,6a,6b,11,11,14b-hexamethyl-1,2,3,4,4a,5,6,6a,6b,7,8,8a,9,10,11,12,12a,14,14a,14b-icosahydropicen-4-yl)methyl 3,5,6-trimethylpyrazine-2-carboxylate (compound **26**): yield 90% (after chromatograph with DCM/MeOH, 1–2%) as a white powder; m.p. 168.1 °C. ^1^H NMR (400 MHz, CDCl_3_) δ 5.28 (s, 1H, H-12), 2.86 (m, 1H, H-18), 2.79 (s, 3H, –CH_3_), 2.55 (d, *J* = 6.2 Hz, 6H, 2 × –CH_3_), 0.99–0.86 (m, 15H, 5 × –CH_3_), 0.75 (s, 3H, –CH_3_). ^13^C NMR (100 MHz, CDCl_3_) δ 177.8, 165.3, 155.4, 152.2, 149.6, 143.9, 138.1, 122.5, 75.3, 74.6, 63.5, 50.7, 47.8, 46.9, 46.0, 44.6, 42.0, 42.0, 41.5, 39.4, 38.2, 37.0, 34.0, 33.2, 33.0, 30.8, 29.5, 27.8, 27.0, 26.2, 26.0, 23.7, 23.5, 23.1, 22.7, 22.3, 21.8, 19.1, 17.2, 15.5, 12.5. HRMS (ESI) *m/z*: [M + H]^+^ 711.4497, calculated for C_42_H_64_ClN_2_O_5_ 711.4498.

### 4.2. Biology Evaluation

MCF-7, A549, HepG2, H9c2, and MDCK cells were obtained from the Chinese Academy of Medical Sciences and Peking Union Medical College. All of the cell lines were maintained in Dulbecco’s modified Eagle medium (DMEM) supplemented with 1% (*v/v*) penicillin/streptomycin and 10% (*v/v*) fetal bovine serum (FBS; Thermo Technologies, New York, NY, USA) under a humidified atmosphere containing 5% CO_2_ at 37 °C. The stock solutions of **He** derivatives were dissolved in dimethyl sulfoxide (DMSO; Sigma, St. Louis, MO, USA) and added at various concentrations to the cell culture. Cellular morphologies were observed using an inverted fluorescence microscope (Olympus IX71, Tokyo, Japan), a plate reader (BIORAD 550 spectrophotometer, Bio-rad Life Science Development Ltd., Beijing, China), and a Canton 2 flow cytometer (BD, New York, NY, USA).

#### 4.2.1. Antitumor Activity

The antitumor activity of these compounds were evaluated on MCF-7, A549, HepG2, MDCK, and H9c2 cell lines using the MTT assay. Cells at a density of 3 × 10^3^ cells/well were plated in a 96-multiwell plate in DMEM containing 10% FBS for 24 h at 37 °C with 5% CO_2_. Then, cells were treated for 72 h with the required concentrations (3.125, 6.25, 12.5, 25, or 50 μM) of the compounds and reference drugs (**He** and **DDP**) dissolved with the vehicle DMSO. After that, the culture substrate was replaced with 200 μL of DMEM containing 20 μL of MTT solution at 5 mg/mL and incubated for 4 h. The supernatant was removed and the dissolved formazans precipitated with 120 μL of DMSO. Wells without drugs were used as blanks. A plate reader was used to determine the absorbance at 490 nm. The above steps were repeated three times. The IC_50_ values were defined as the concentration of the compound which gave 50% growth inhibition, and they were calculated using Graph Pad Prism 5. The inhibition rate was calculated using the following formula, where OD is the optical density:

% inhibition = 1 − (Sample group OD/Control group OD) × 100%. (1)

#### 4.2.2. Giemsa Staining

A549 cells in the logarithmic growth phase were seeded in 12-well plates (1.2 × 10^4^ cells/well). After incubation for 24 h at 37 °C with 5% CO_2_, various concentrations (0, 2, 4, or 6 μM) of compound **9** were added to the cultures, and the plate was incubated for further 72 h. Then, we discarded the cell culture fluid, washed twice with phosphate-buffered saline (PBS), and fixed the cells with cold methanol. The A549 cells were stained with 6% Giemsa solution for 5 min, washed with water, and dried. The cell morphological changes were observed under an inverted microscope.

#### 4.2.3. DAPI Staining

A549 cells in the logarithmic growth phase were seeded in 12-well plates (1.2 × 10^4^ cells/well). After incubation for 24 h at 37 °C with 5% CO_2_, various concentrations (0, 2, 4, or 6 μM) of compound **9** were added to the cultures, and the plate was incubated for further 72 h. Then, we discarded the cell culture fluid, washed twice with PBS, and fixed the cells with 4% paraformaldehyde for 10 min. After that, we added the appropriate amount of DAPI (1 mg/mL; Molecular Probes/Invitrogen Life Technologies, Carlsbad, CA, USA) to cover the bottom of the board, after staining for 5 min in the dark. Nuclear fragments were observed at 450 nm under an inverted fluorescence microscope (Olympus IX71, Tokyo, Japan).

#### 4.2.4. Annexin V-FITC/PI Staining

According to instructions, A549 cells were stained with an annexin-V FITC apoptosis detection kit (Beijing BioDee Biotech. Co., Ltd., Beijing, China). A549 cells in the logarithmic growth phase were seeded in six-well plates (4.8 × 10^4^ cells/well). After incubation for 24 h at 37 °C with 5% CO_2_, various concentrations (0, 2, 5, or 10 μM) of compound **9** were added to the cultures, and the plate was incubated for a further 72 h. A549 cells were collected, washed twice with cold PBS, and centrifuged at 2400 rpm for 10 min. The resulting pellet was mixed with 200 μL of binding buffer of the Annexin V-FITC kit; then, 5 μL of FITC-labeled annexin V was added and mixed gently. After incubation at 4 °C for 10 min in the dark, 5 μL of PI was added. Then, the cells were immediately analyzed with a flow cytometer at 488 nm.

#### 4.2.5. Cell Cycle

According to instructions, A549 cells were stained with the Cell Cycle and Apoptosis Analysis Kit (Beyotime Biotechnology, Shanghai, China). A549 cells in the logarithmic growth phase were seeded in six-well plates (4.8 × 10^4^ cells/well). After incubation for 24 h at 37 °C with 5% CO_2_, various concentrations (0, 2, 5, or 10 μM) of compound **9** were added to the cultures, and the plate was incubated for a further 72 h. A549 cells were collected, washed twice with cold PBS, and centrifuged at 2400 rpm for 10 min. The resulting pellet was mixed with 1 mL of 70% ethanol, and retained for 12 h at 4 °C, before being centrifuged at 2400 rpm for 10 min. The supernatant was discarded, and the resulting pellet was mixed with 1mL of cold PBS and centrifuged at 2400 rpm for 10 min. Then, 0.5 mL of PI staining solution was added and mixed gently. After incubation at 37 °C for 30 min, the cells were immediately analyzed with a flow cytometer at 488 nm.

## Figures and Tables

**Table 1:** The in vitro cytotoxicity of compounds against various cell lines. A549—human non-small-cell lung cancer; MCF-7—human mammary cancer; HepG2—human hepatocellular carcinoma; MDCK—Madin–Darby canine kidney; H9c2—murine heart myoblast; **He**—hederagenin; **DDP**—cisplatin.

Half Maximal Inhibitory Concentration (IC_50_) Values (μM)					
Compound	A549	MCF-7	HepG2	MDCK	H9c2
**1**	17.62 ± 0.37	16.05 ± 0.01	18.79 ± 0.04	18.06 ± 0.25	14.73 ± 0.51
**2**	20.55 ± 0.29	15.78 ± 0.22	27.23 ± 1.82	26.71 ± 1.64	25.09 ± 0.43
**3**	15.03 ± 0.35	16.83 ± 9.25	18.93 ± 0.35	18.42 ± 3.76	>30
**4**	17.07 ± 1.06	17.31 ± 0.48	25.53 ± 1.40	26.12 ± 1.02	20.42 ± 2.24
**5**	24.37 ± 0.37	>30	24.94 ± 0.06	23.53 ± 0.67	28.46 ± 0.29
**6**	25.52 ± 0.26	>30	>30	24.08 ± 0.76	>30
**7**	>30	>30	>30	>30	>30
**8**	23.92 ± 0.31	25.35 ± 0.09	17.41 ± 0.75	20.13 ± 0.30	21.66 ± 0.86
**9**	3.45 ± 0.59	8.73 ± 1.49	8.71 ± 0.38	14.11 ± 0.04	16.69 ± 0.12
**10**	20.93 ± 0.68	27.24 ± 2.09	28.17 ± 0.21	25.42 ± 0.28	19.60 ± 3.29
**11**	14.12 ± 0.01	27.30 ± 0.57	14.84 ± 0.03	18.64 ± 0.18	14.72 ± 0.16
**12**	10.05 ± 2.47	17.28 ± 0.58	14.42 ± 0.03	18.64 ± 0.71	13.16 ± 0.20
**13**	8.15 ± 0.17	18.03 ± 0.18	13.24 ± 0.29	23.22 ± 0.01	10.51 ± 0.61
**14**	17.60 ± 2.33	31.23 ± 0.15	7.05 ± 0.17	18.10 ± 0.12	15.90 ± 0.22
**15**	17.98 ± 0.04	19.77 ± 1.27	6.71 ± 0.20	17.12 ± 1.01	17.56 ± 1.85
**16**	24.78 ± 0.66	28.49 ± 0.28	6.99 ± 0.63	20.12 ± 0.06	14.99 ± 0.12
**17**	19.57 ± 0.22	22.88 ± 0.66	8.15 ± 0.22	20.93 ± 3.03	22.31 ± 0.30
**18**	16.17 ± 0.15	28.29 ± 0.41	11.91 ± 0.99	16.55 ± 0.22	>30
**19**	12.23 ± 0.4	27.57 ± 0.52	9.06 ± 0.32	16.43 ± 0.04	16.50 ± 0.22
**20**	>30	>30	>30	>30	>30
**21**	9.29 ± 0.99	24.98 ± 0.18	7.99 ± 0.12	14.85 ± 0.06	16.68 ± 0.10
**22**	10.40 ± 0.43	20.53 ± 3.63	7.15 ± 0.43	16.34 ± 1.57	16.26 ± 0.07
**23**	18.67 ± 0.32	21.26 ± 1.28	7.17 ± 1.09	19.49 ± 2.47	23.35 ± 0.55
**24**	21.97 ± 0.87	17.95 ± 2.80	6.89 ± 0.21	19.27 ± 1.47	27.87 ± 0.62
**25**	>30	15.63 ± 1.41	6.42 ± 0.24	>30	>30
**26**	>30	18.30 ± 1.16	7.15 ± 0.85	>30	>30
**He**	>50	>50	>50	>50	>50
**DDP**	3.85 ± 0.63	5.17 ± 0.28	3.42 ± 0.68	9.97 ± 1.12	5.31 ± 0.26

**Table 2:** The evaluation of explained variation (*R*^2^*X*) and predictive ability (*Q*^2^_(cum)_).

Cell Types	*R* ^2^ *X*	*Q* ^2^_(cum)_
A549	0.761	0.159
HepG2	0.872	0.697
MCF-7	0.748	0.148

## Supplementary Materials

Supplementary materials can be found at http://www.mdpi.com/1422-0067/19/10/2994/s1.
