# Supplementary material for: Design, Synthesis, and Cytotoxic Analysis of Novel Hederagenin–Pyrazine Derivatives Based on Partial Least Squares Discriminant Analysis
Source: Int J Mol Sci. 2018 Sep 30;19(10):2994. doi: 10.3390/ijms19102994 (PMC6213900; doi:10.3390/ijms19102994)

Journal Name: *International Journal of Molecular Sciences*

Supplementary Material for compounds :

He

^{1}H NMR spectra of He

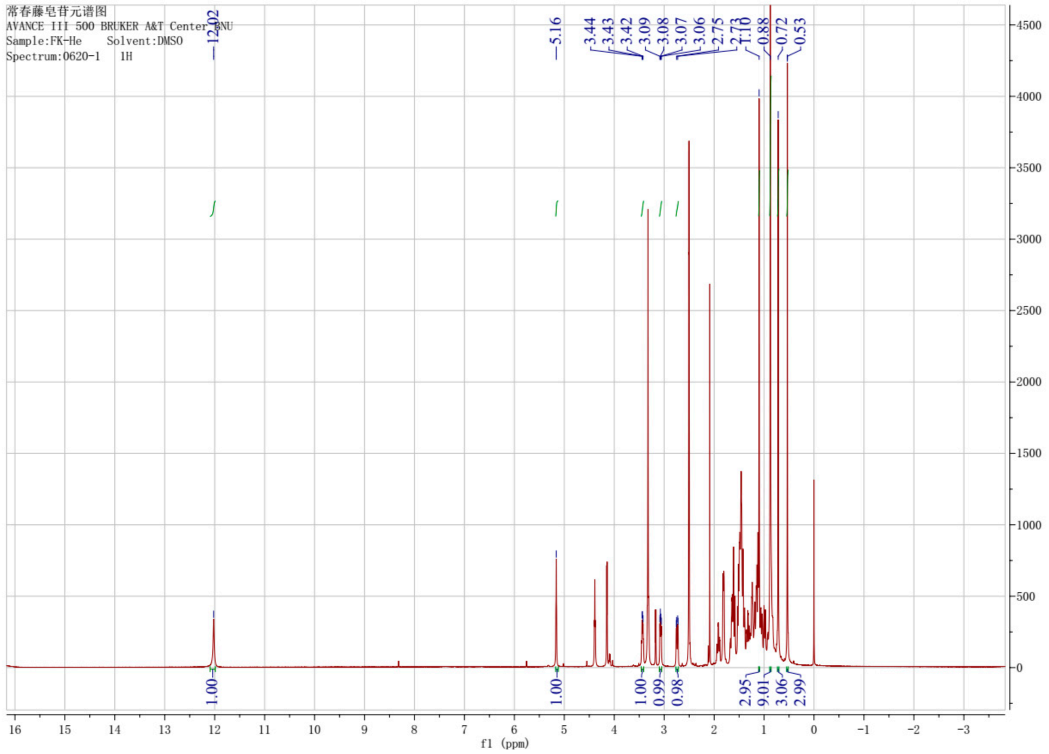

^{13}C NMR spectra of He

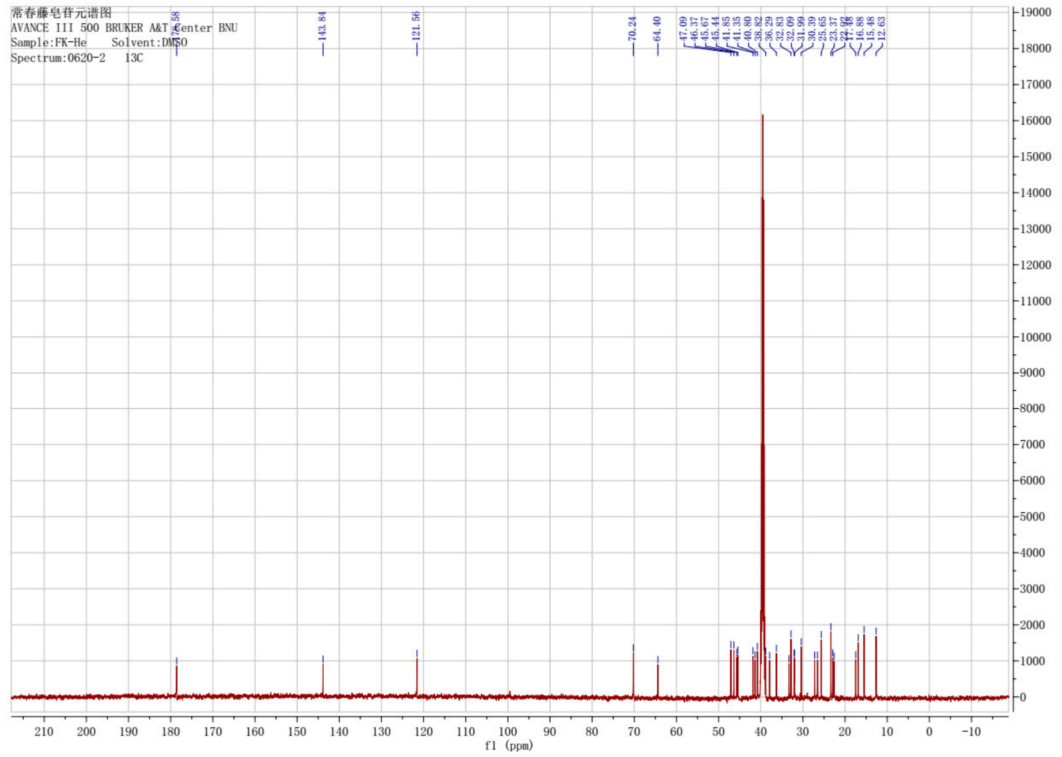

Compound **1**

^{1}H NMR spectra of Compound **1**

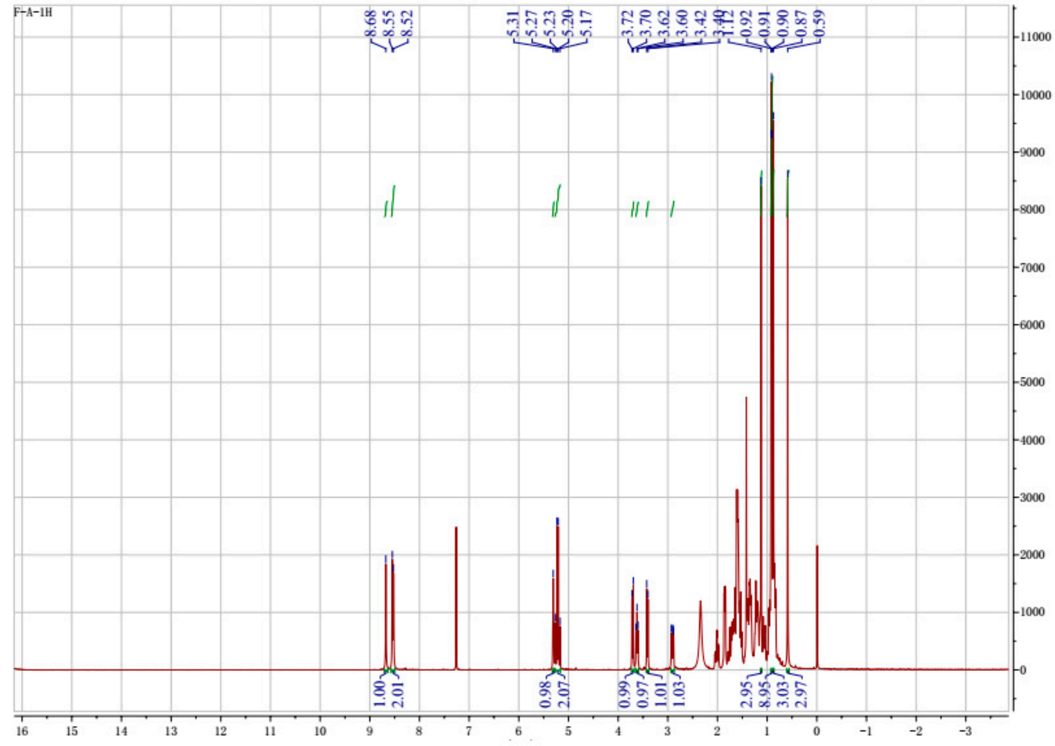

^{13}C NMR spectra of Compound **1**

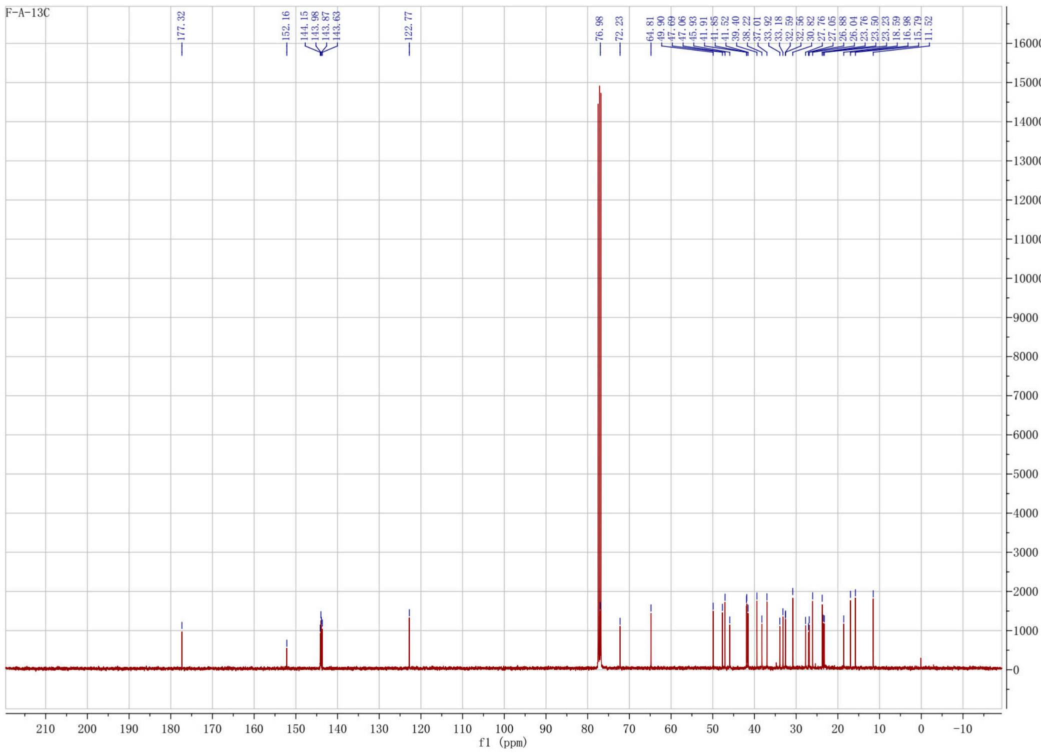

Compound **2**

^{1}H NMR spectra of Compound **2**

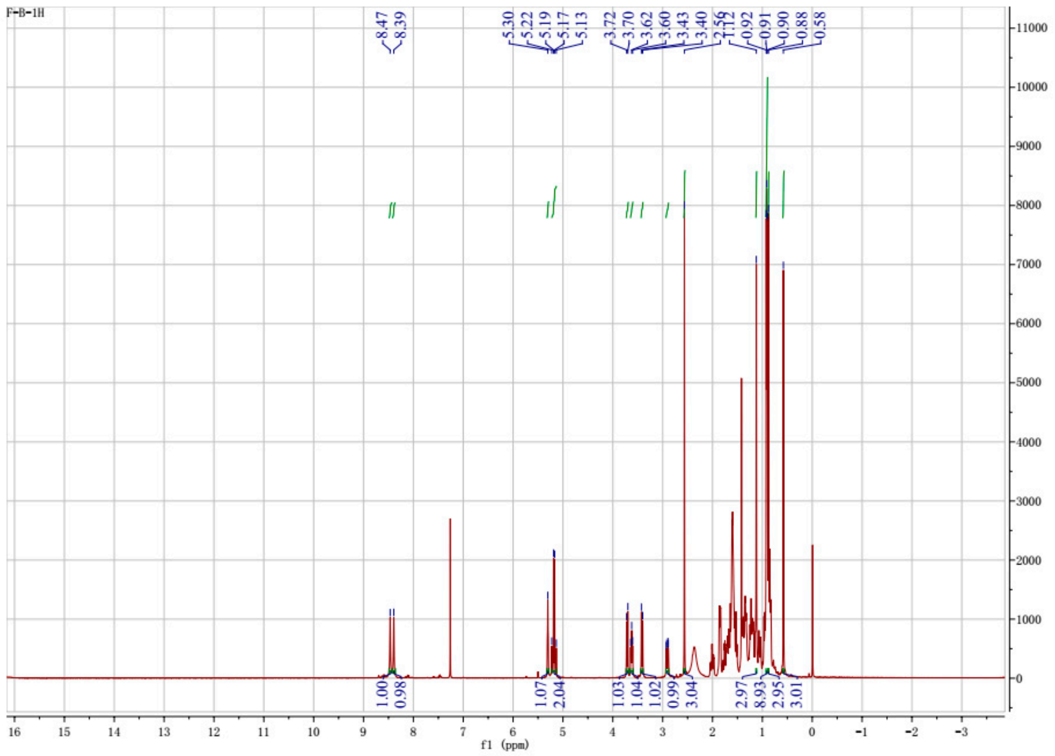

^{13}C NMR spectra of Compound **2**

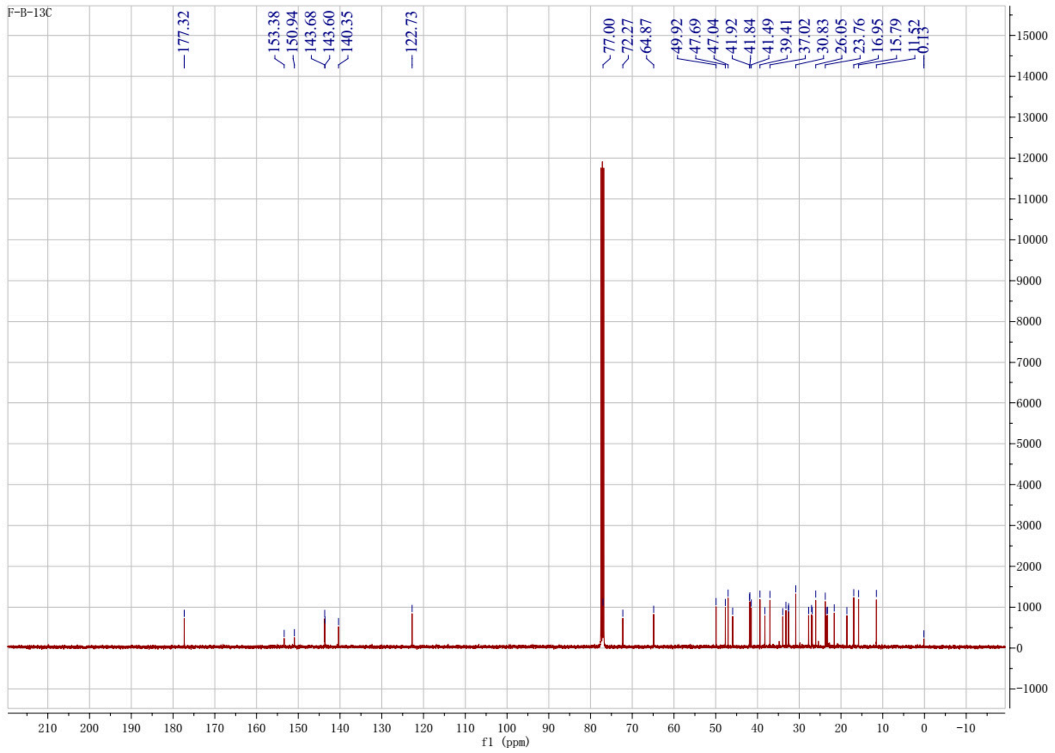

Compound **3**

^{1}H NMR spectra of Compound **3**

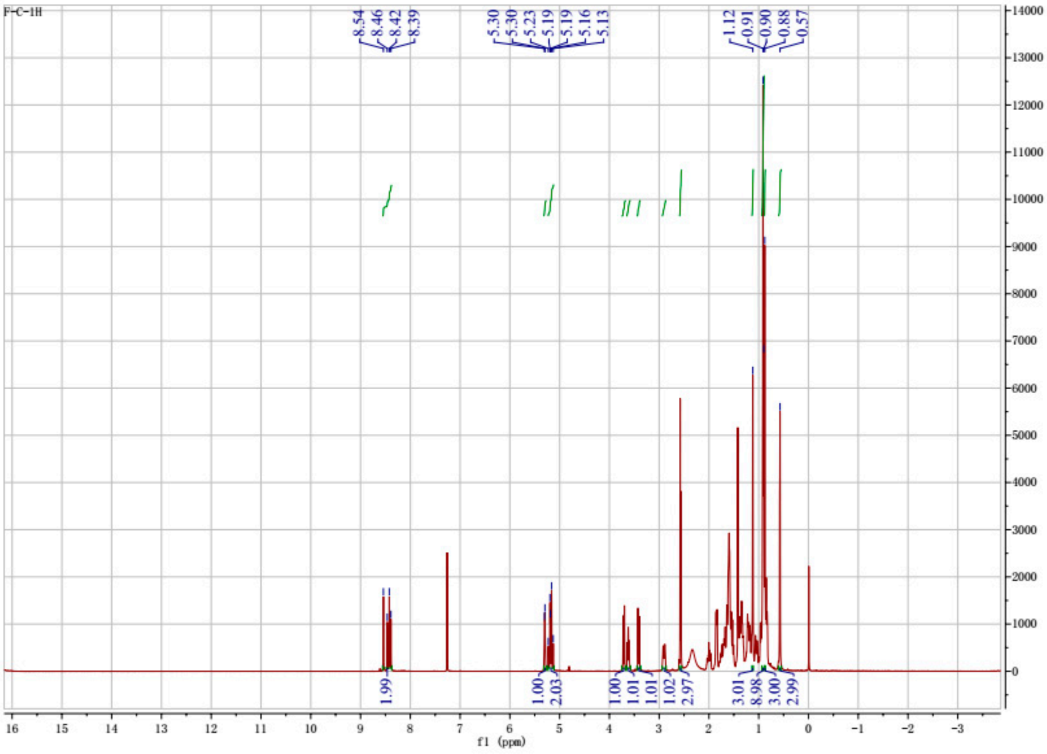

^{13}C NMR spectra of Compound **3**

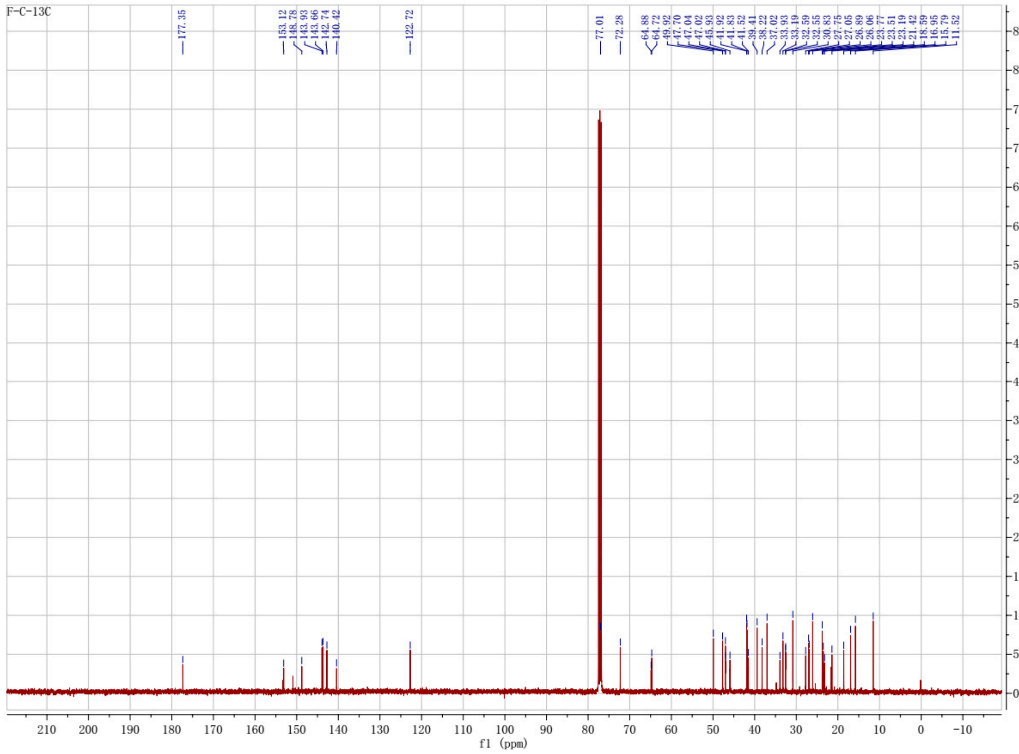

Compound **4**

^{1}H NMR spectra of Compound **4**

^{13}C NMR spectra of Compound **4**

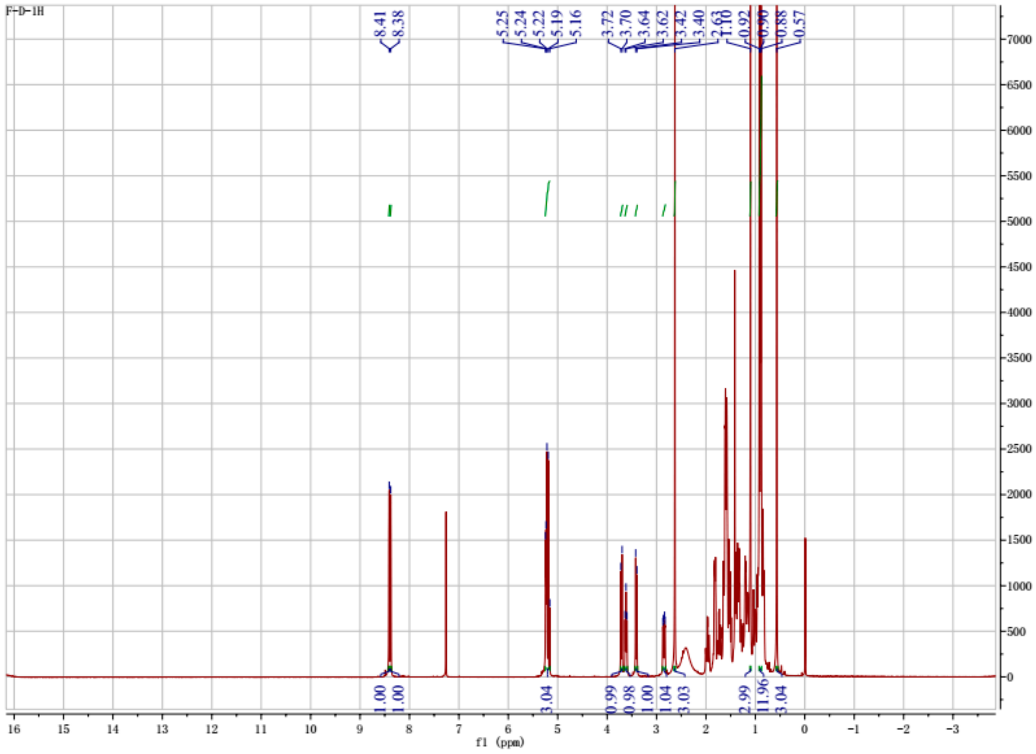

Compound **5**

^{1}H NMR spectra of Compound **5**

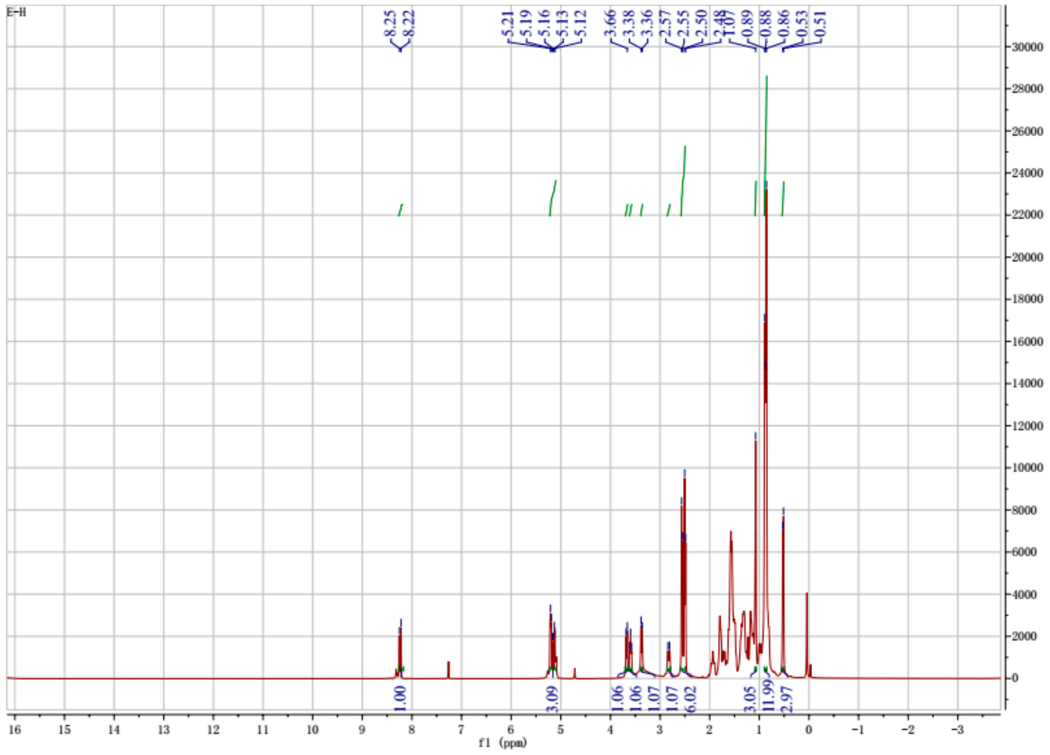

^{13}C NMR spectra of Compound **5**

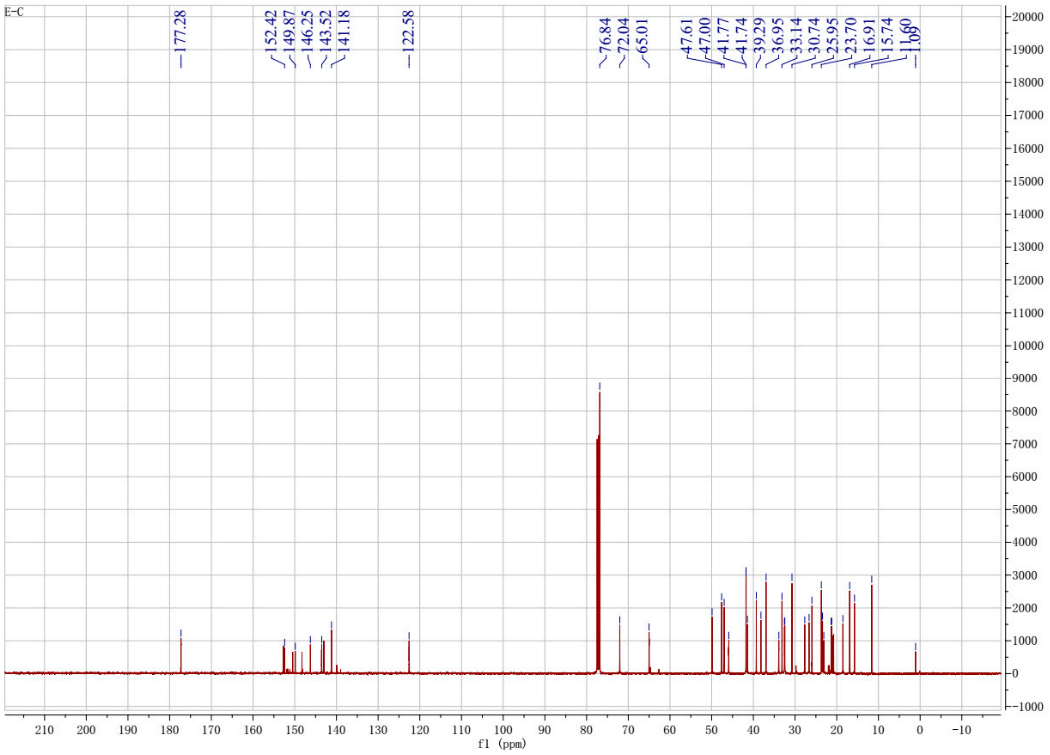

Compound **6**

^{1}H NMR spectra of Compound **6**

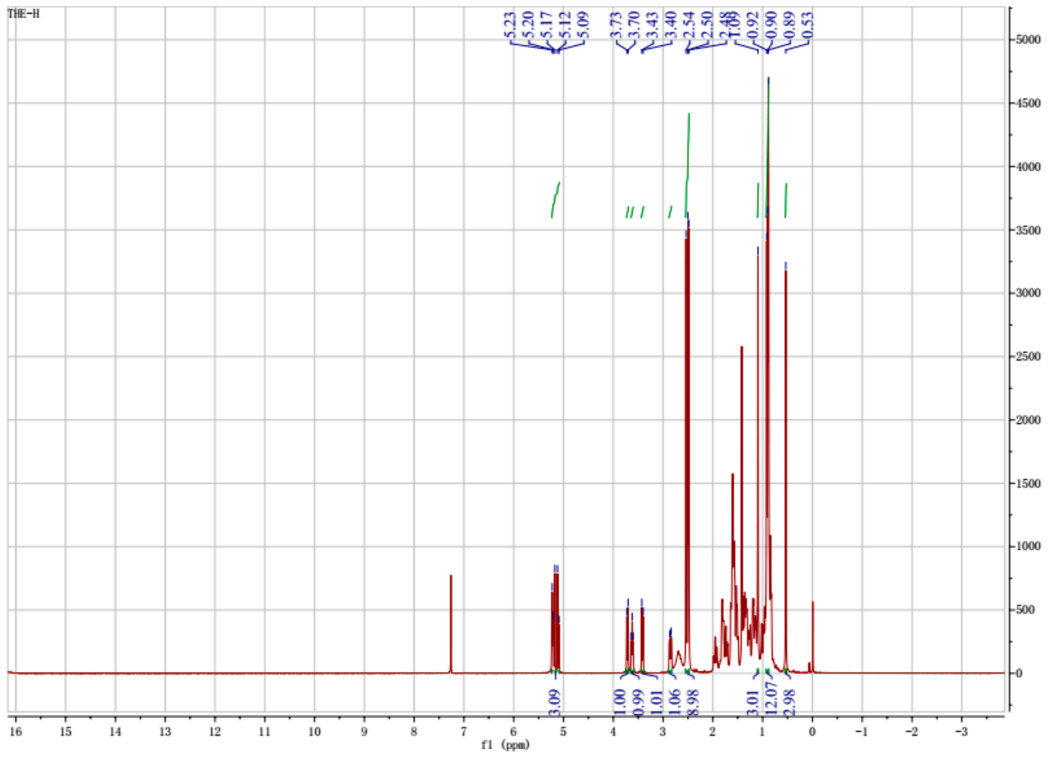

^{13}C NMR spectra of Compound **6**

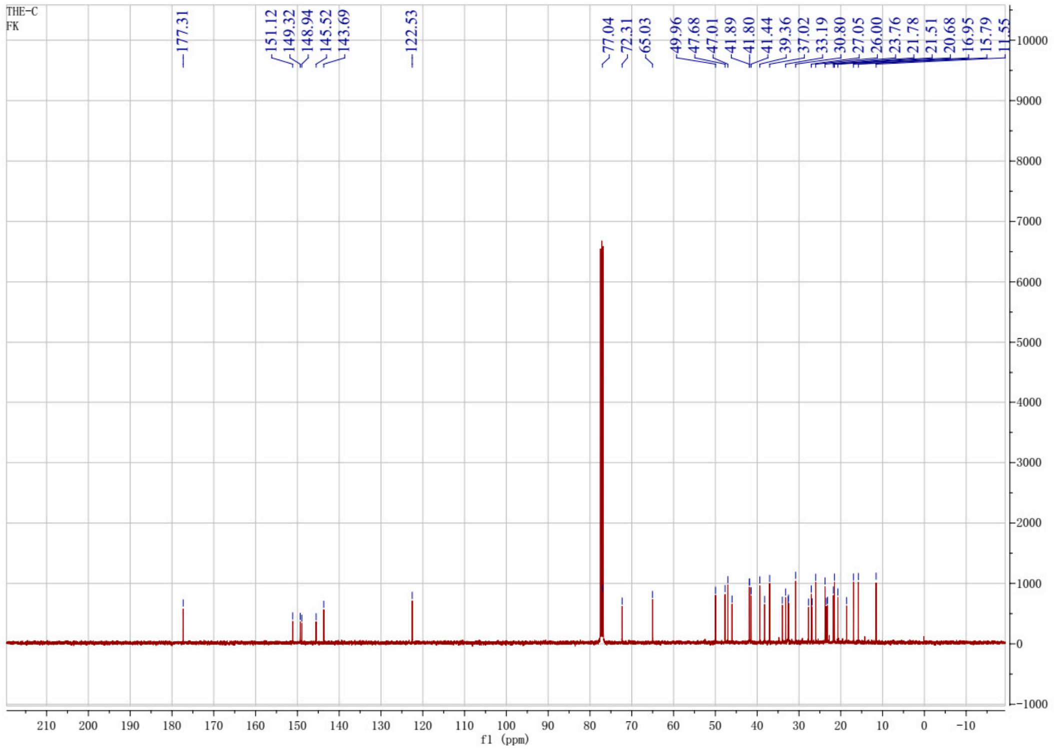

Compound **7**

^{1}H NMR spectra of Compound **7**

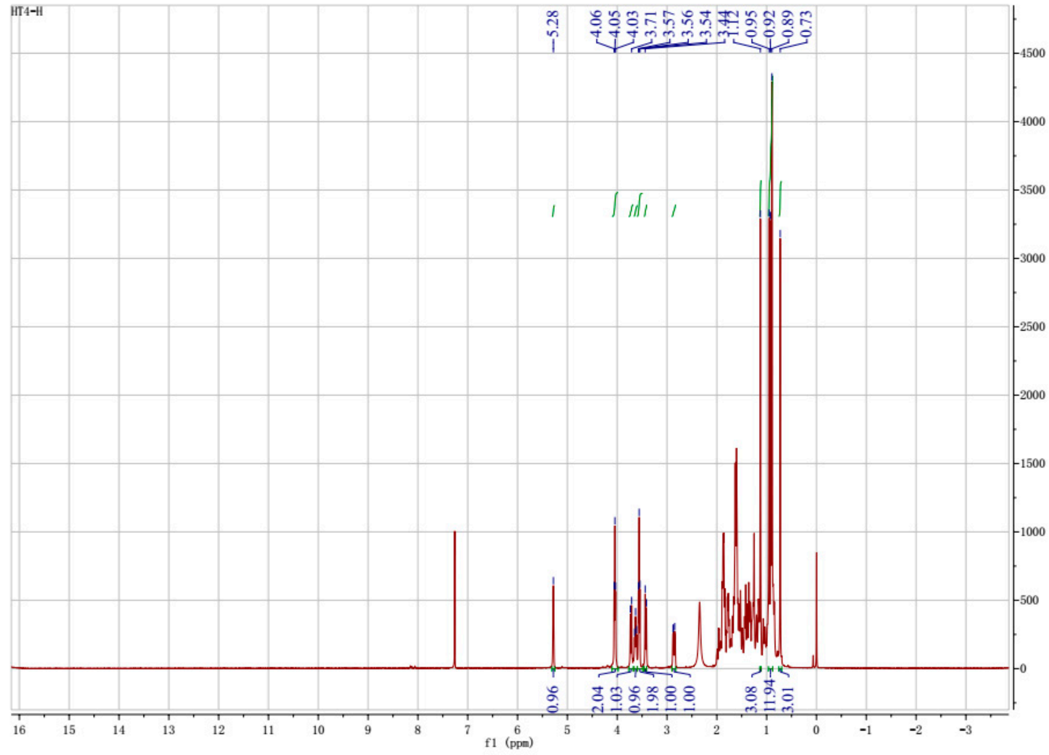

^{13}C NMR spectra of compound **7**

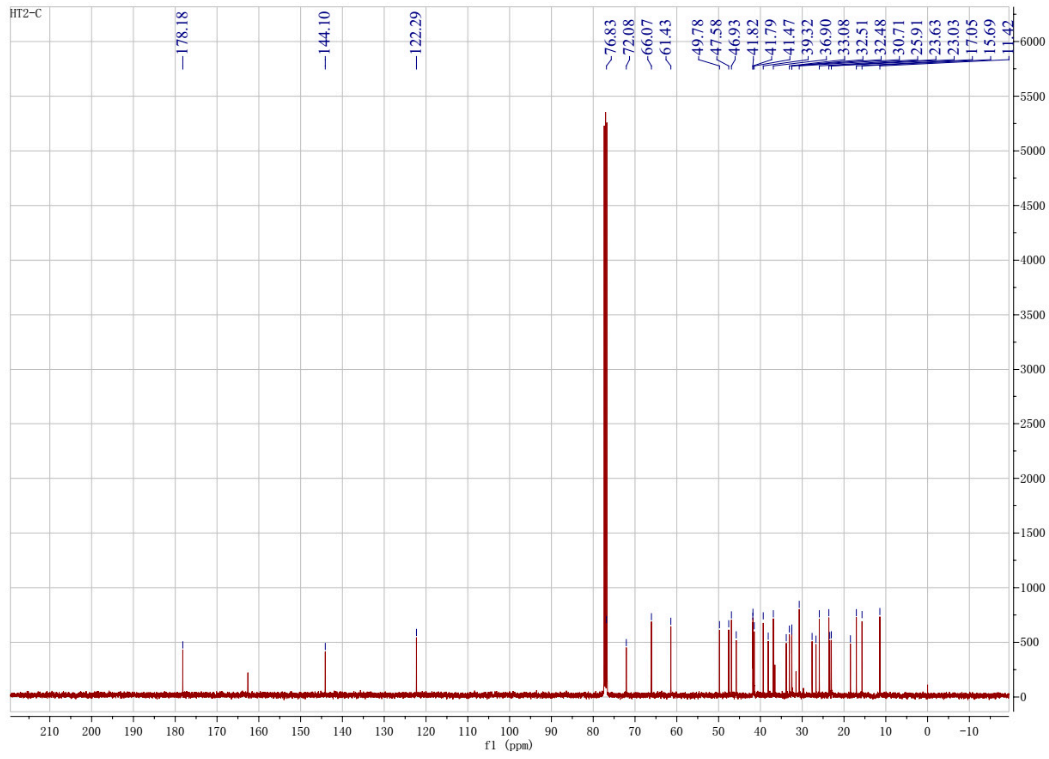

Compound 8

^{1}H NMR spectra of compound **8**

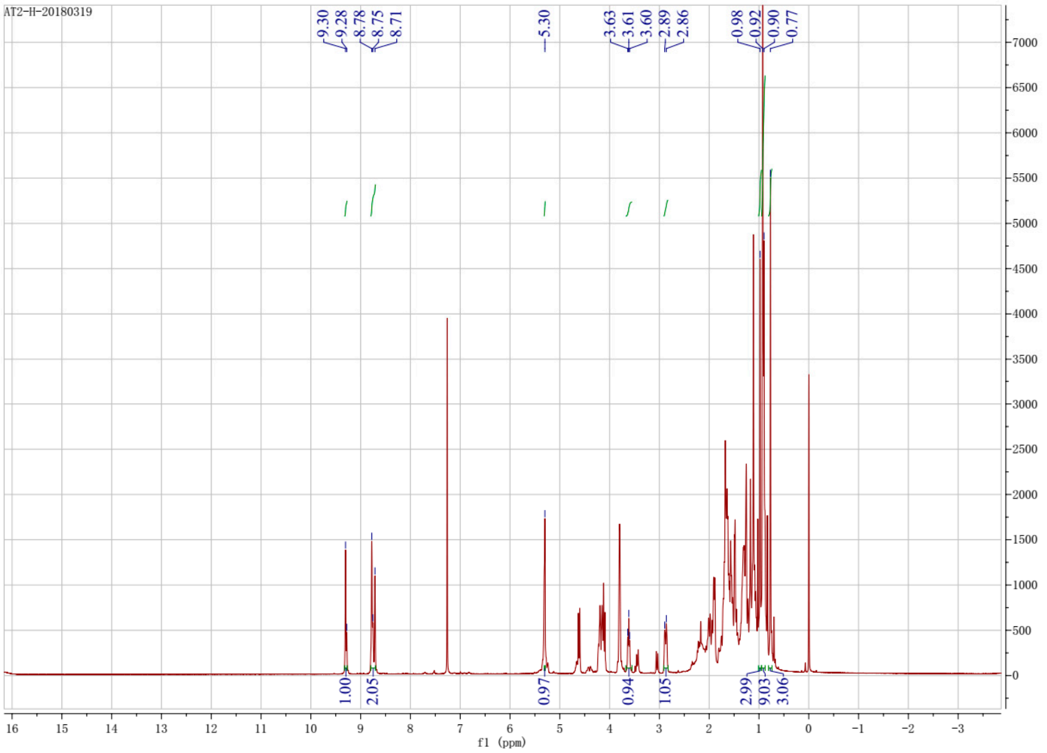

^{13}C NMR spectra of compound **8**

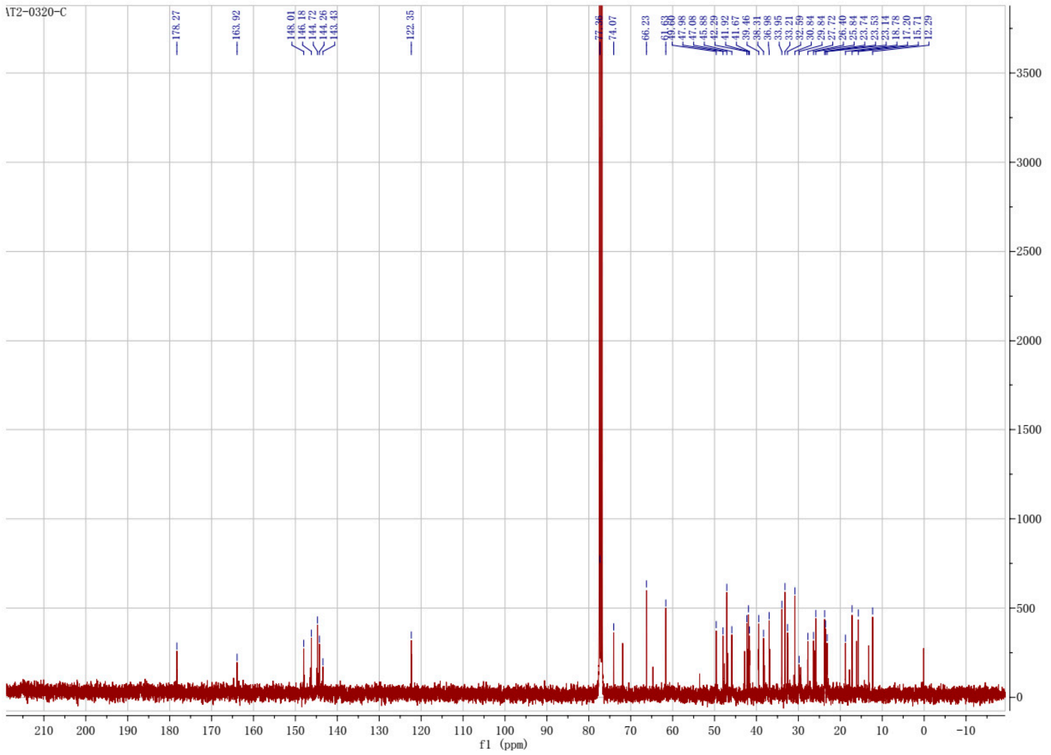

Compound **9**

^{1}H NMR spectra of compound **9**

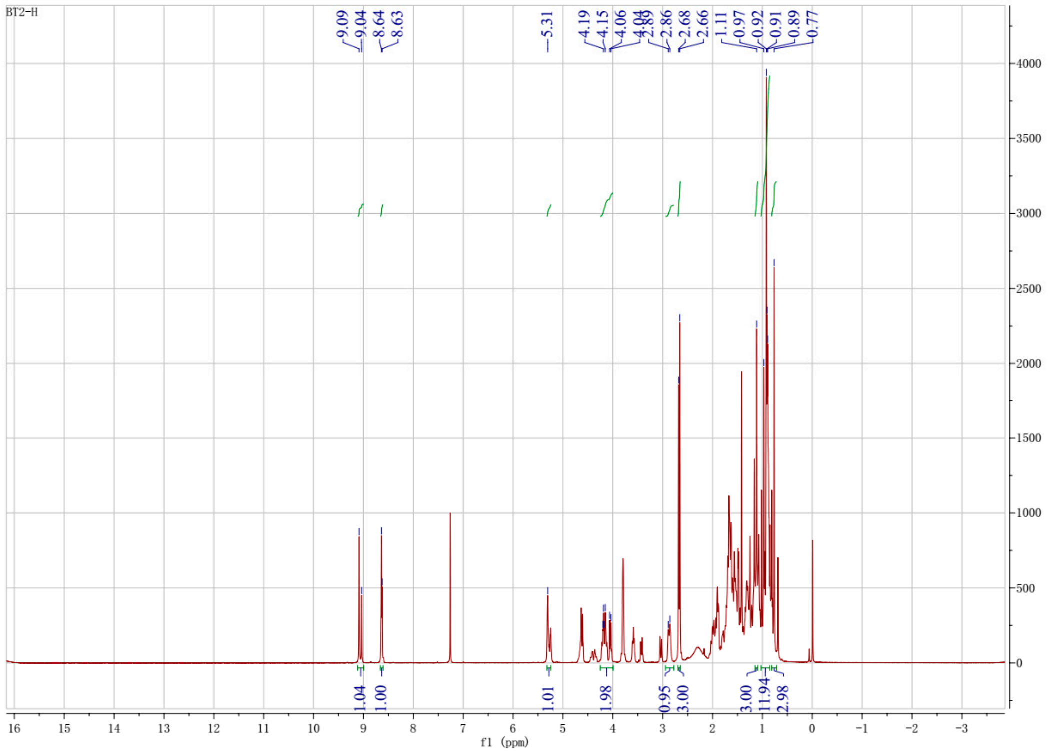

^{13}C NMR spectra of compound **9**

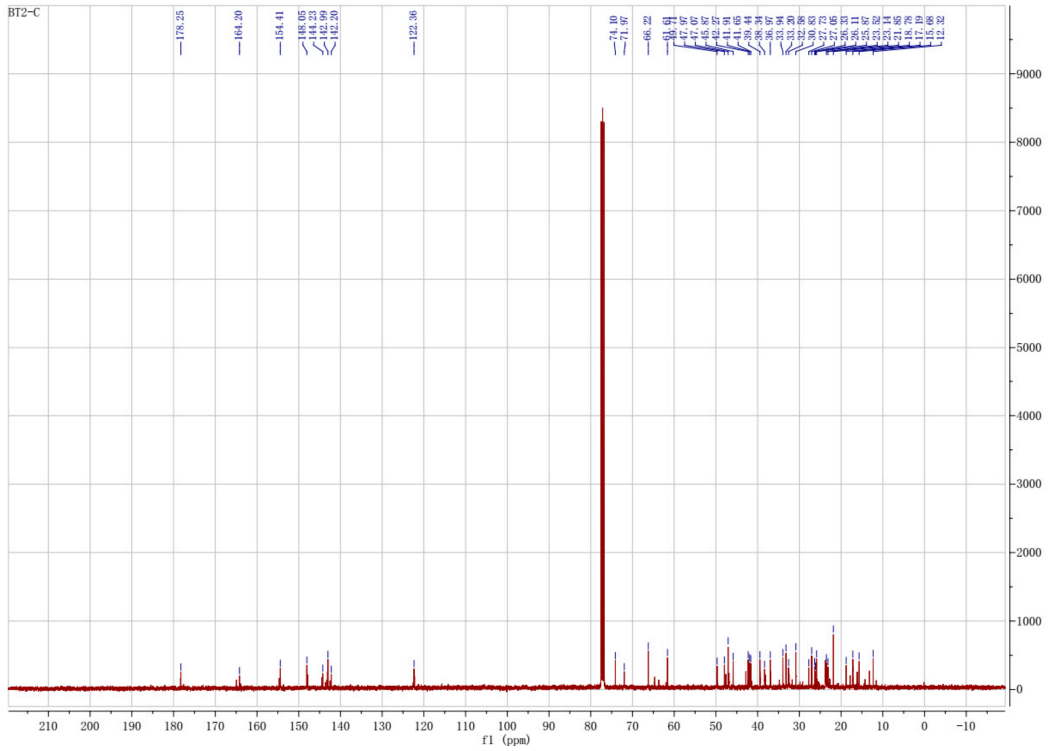

Compound **10**

^{1}H NMR spectra of compound **10**

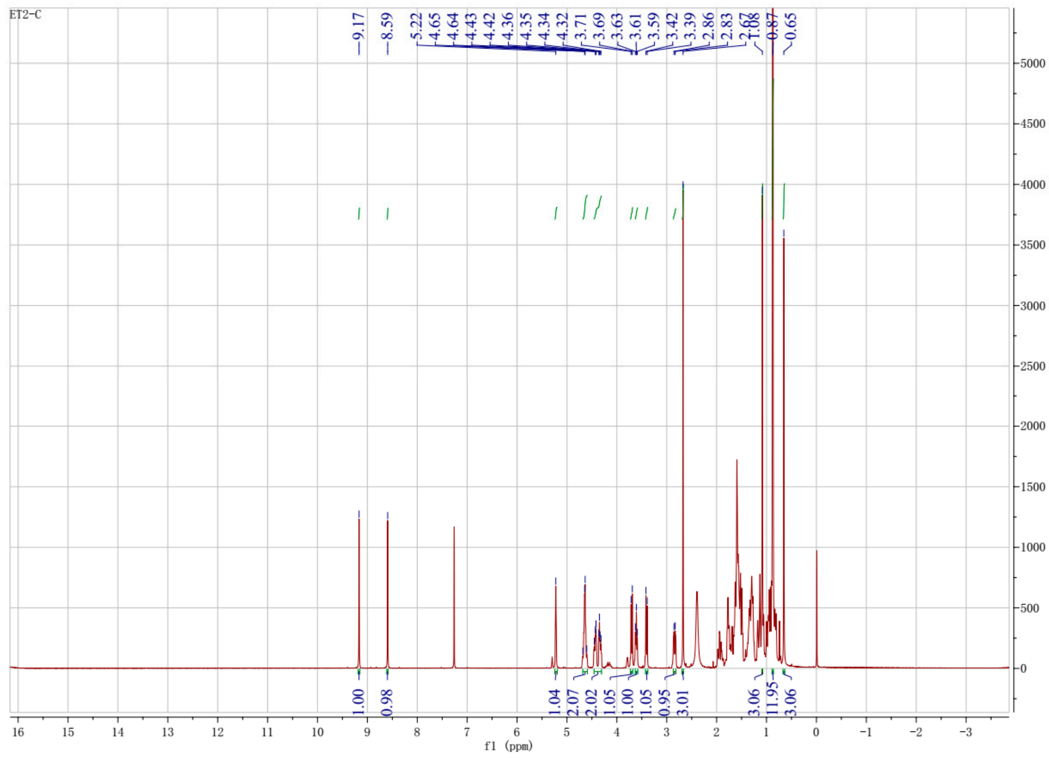

^{13}C NMR spectra of compound **10**

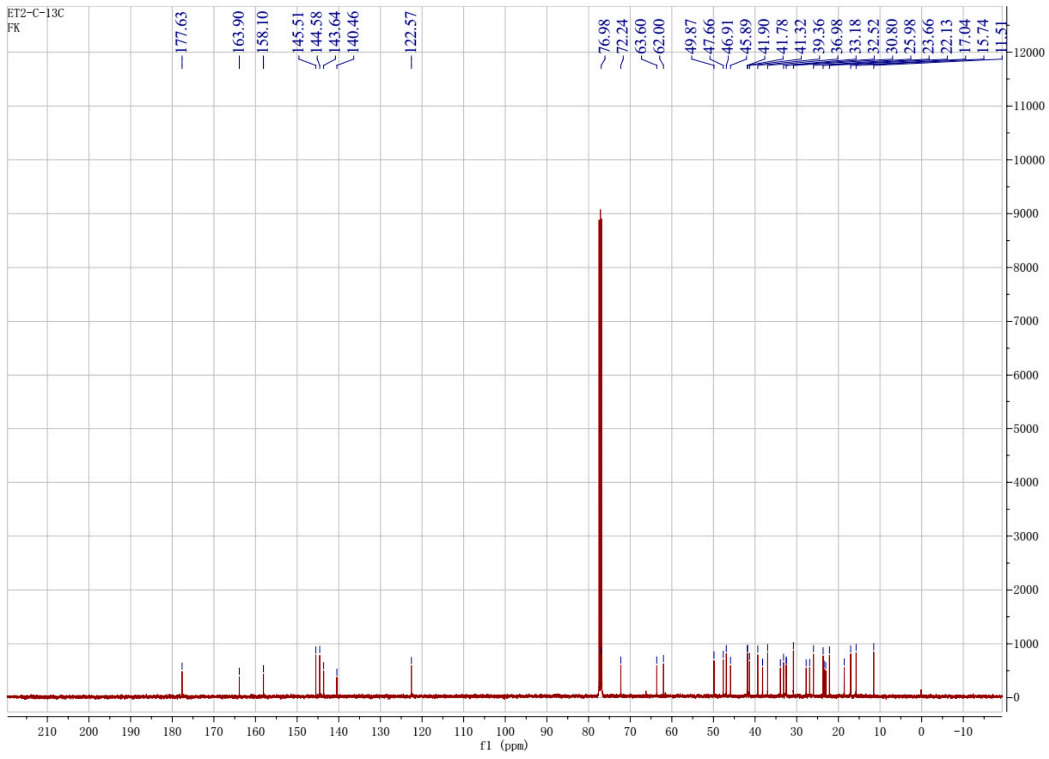

Compound **11**

^{1}H NMR spectra of compound **11**

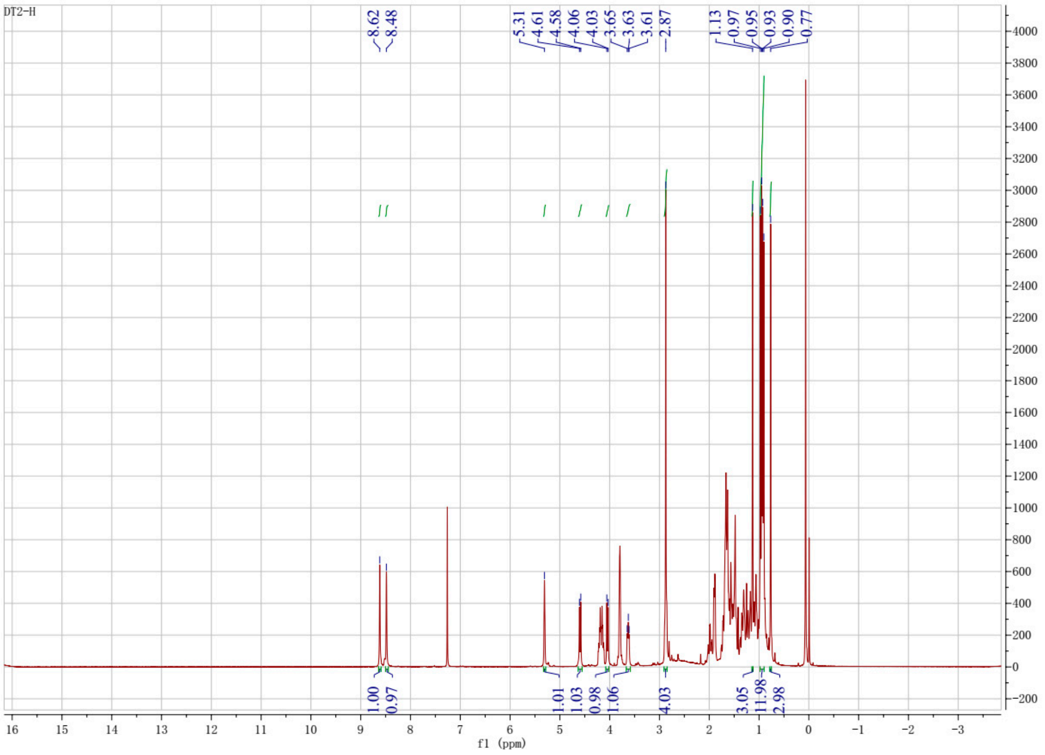

^{13}C NMR spectra of compound **11**

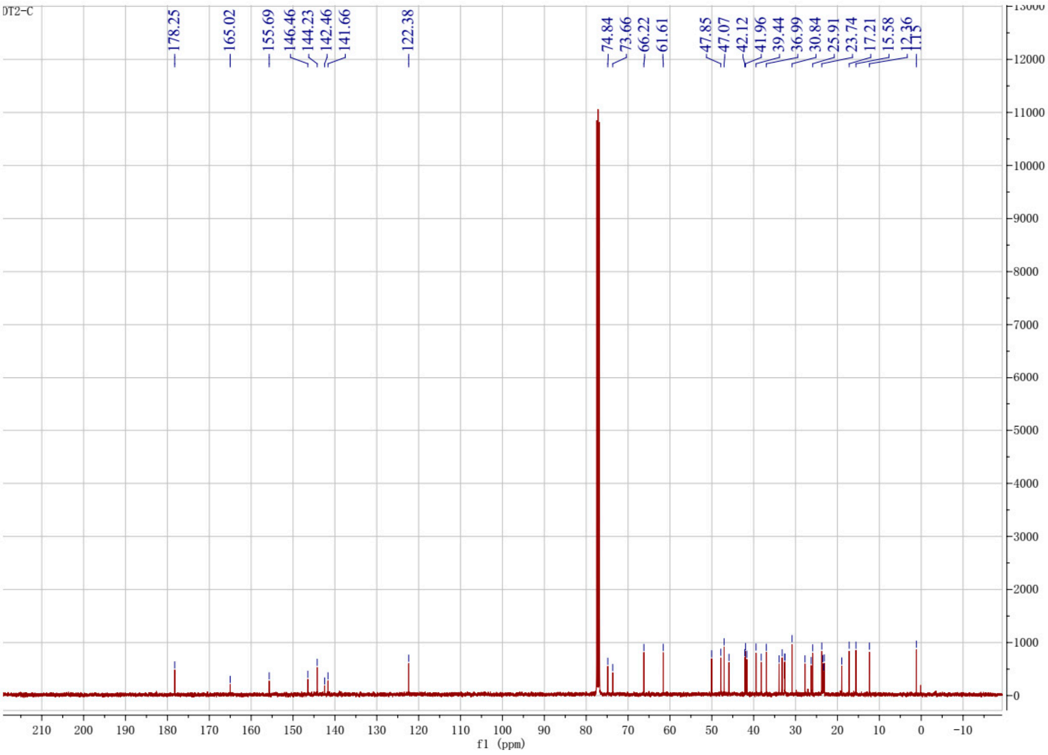

Compound **12**

^{1}H NMR spectra of compound **12**

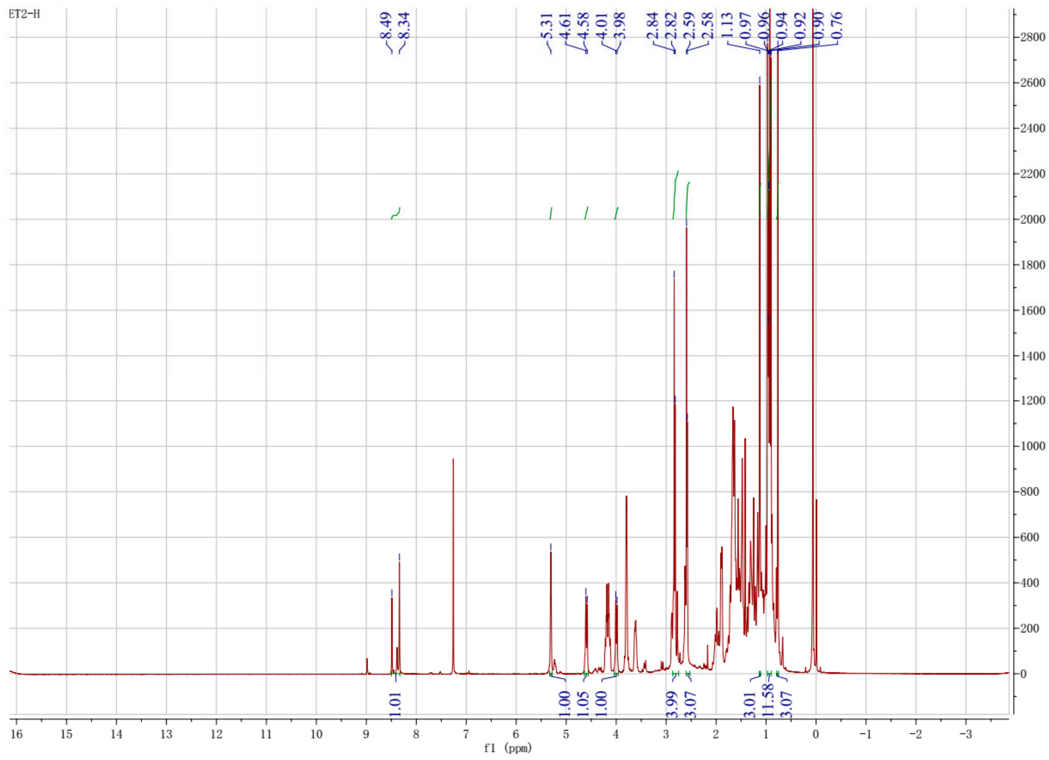

^{13}C NMR spectra of compound **12**

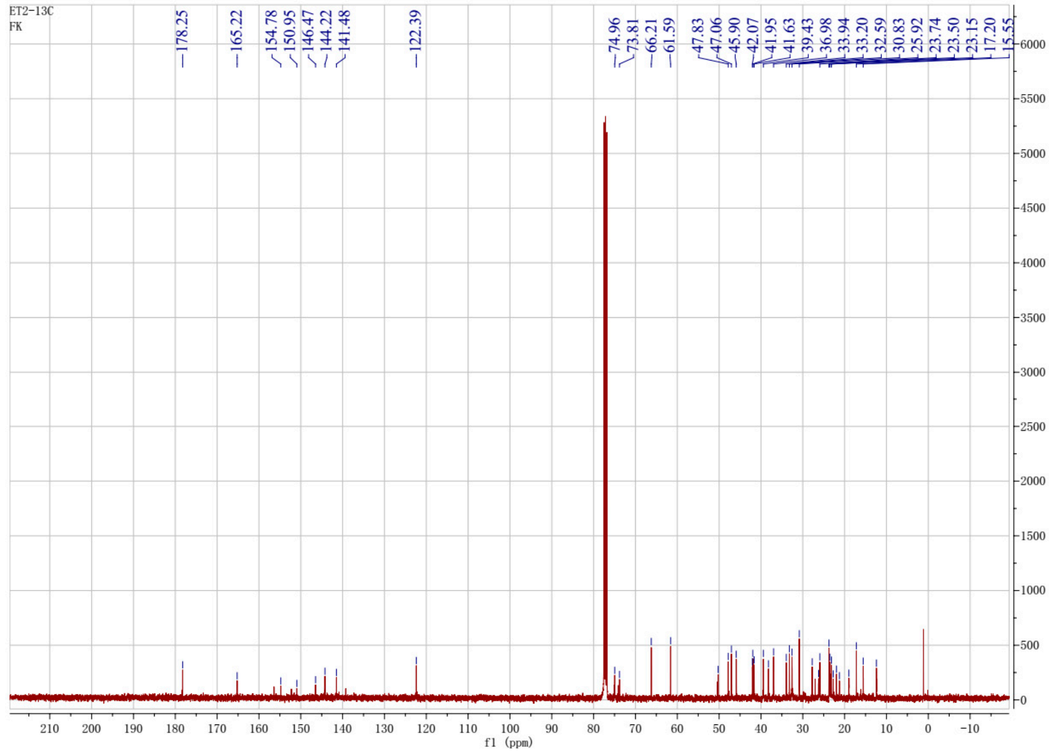

Compound **13**

^{1}H NMR spectra of compound **13**

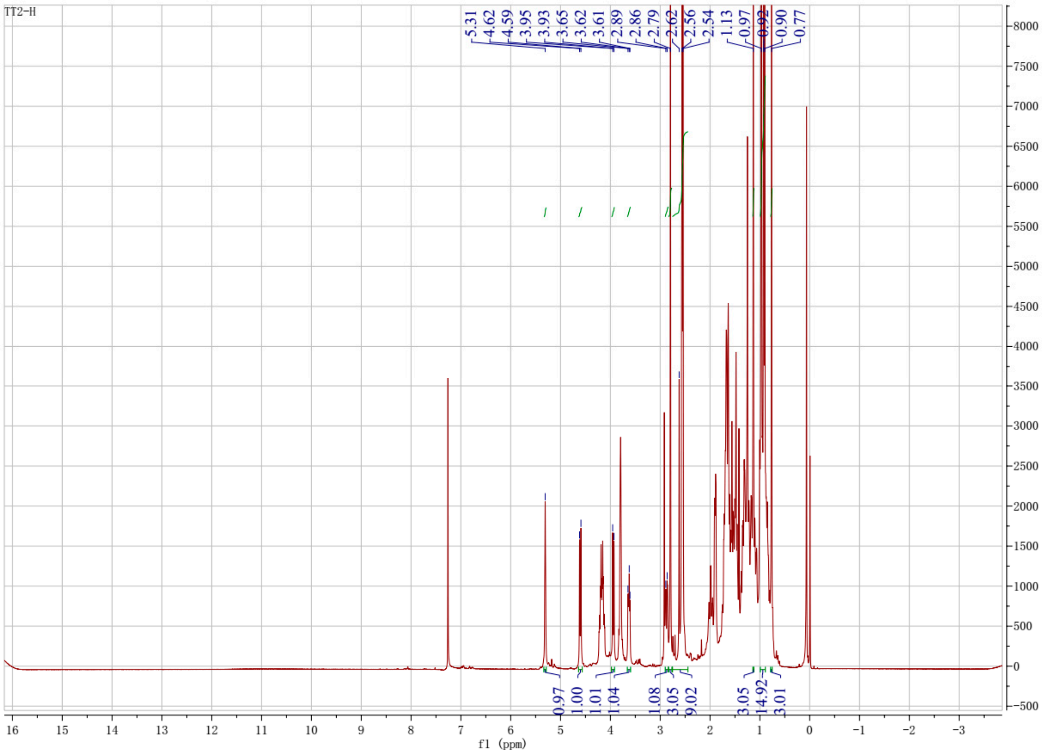

^{13}C NMR spectra of compound **13**

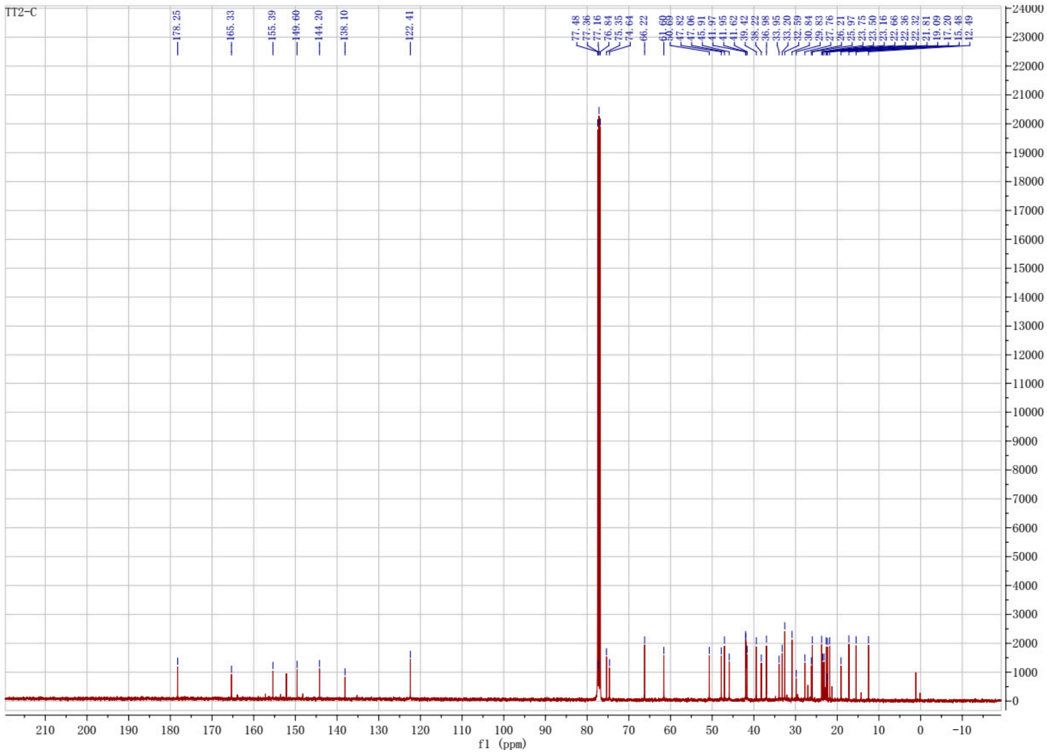

Compound **14**

^{1}H NMR spectra of compound **14**

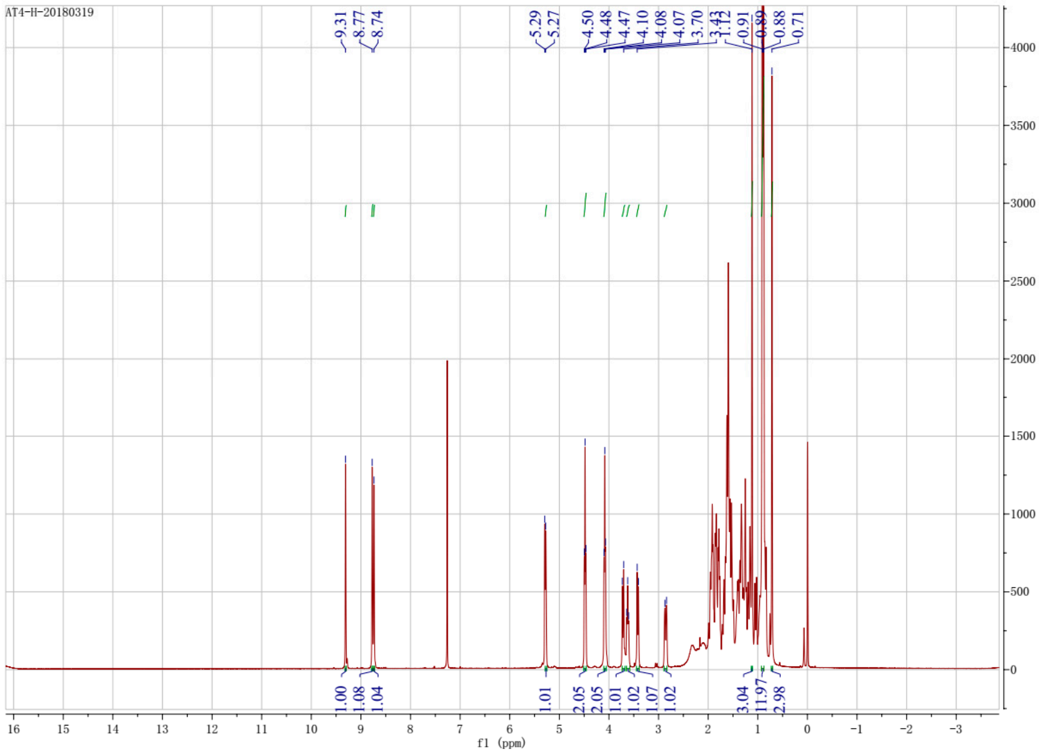

^{13}C NMR spectra of compound **14**

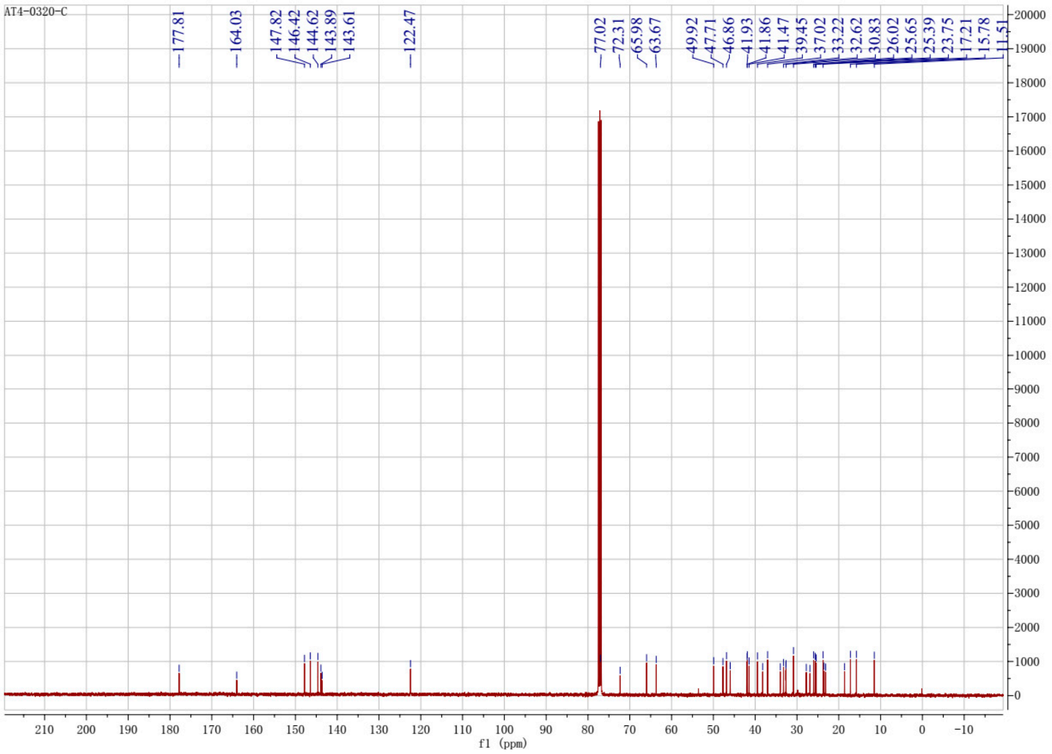

Compound **15**

^{1}H NMR spectra of compound **15**

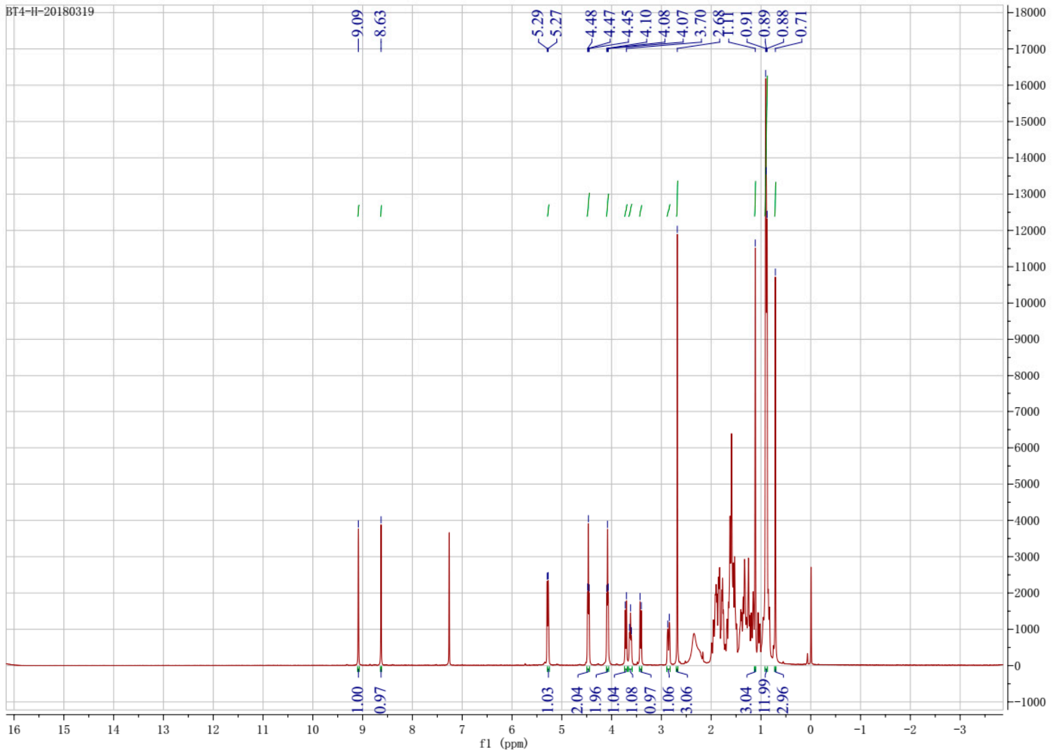

^{13}C NMR spectra of compound **15**

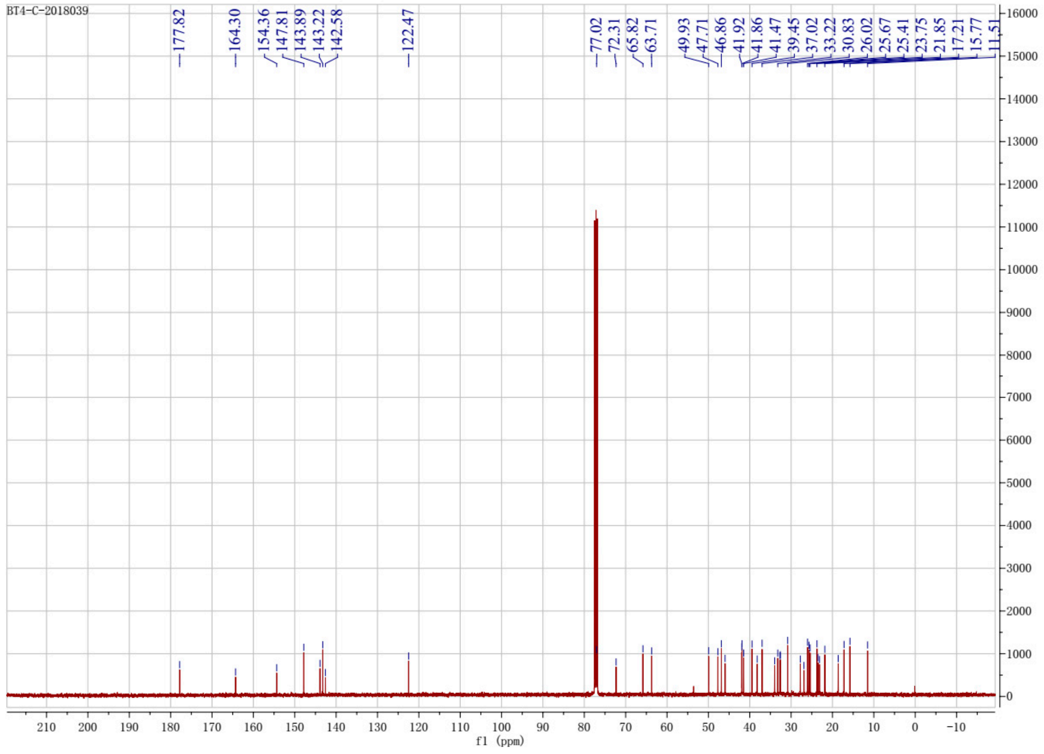

Compound **16**

^{1}H NMR spectra of compound **16**

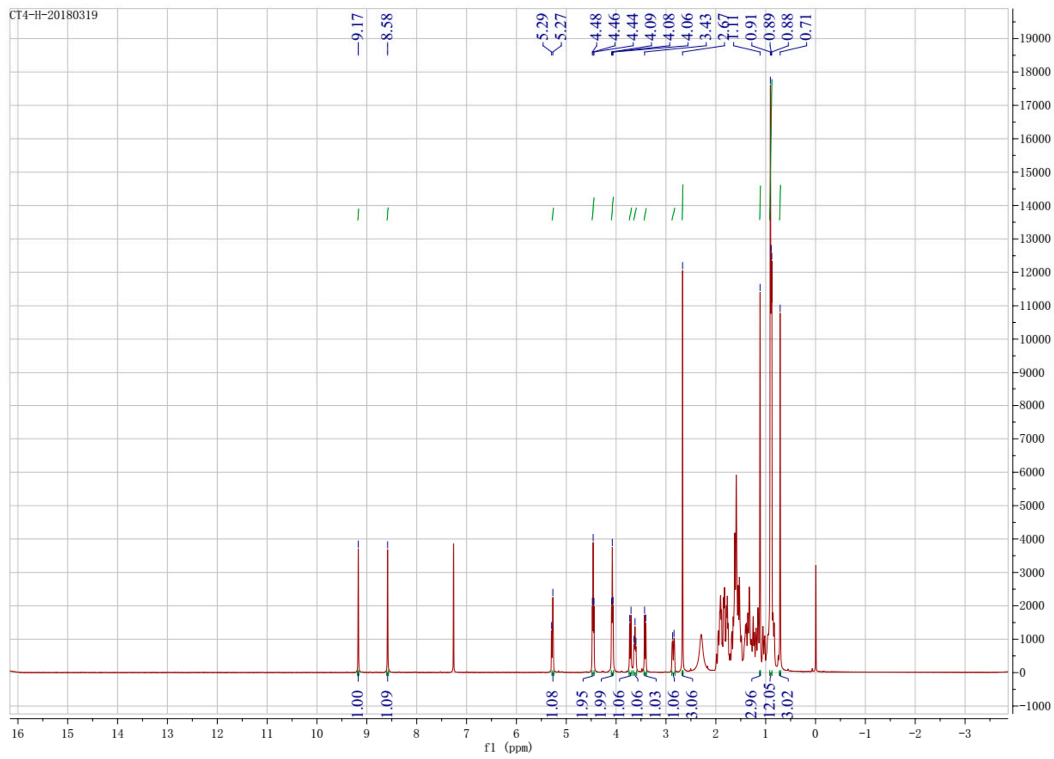

^{13}C NMR spectra of compound **16**

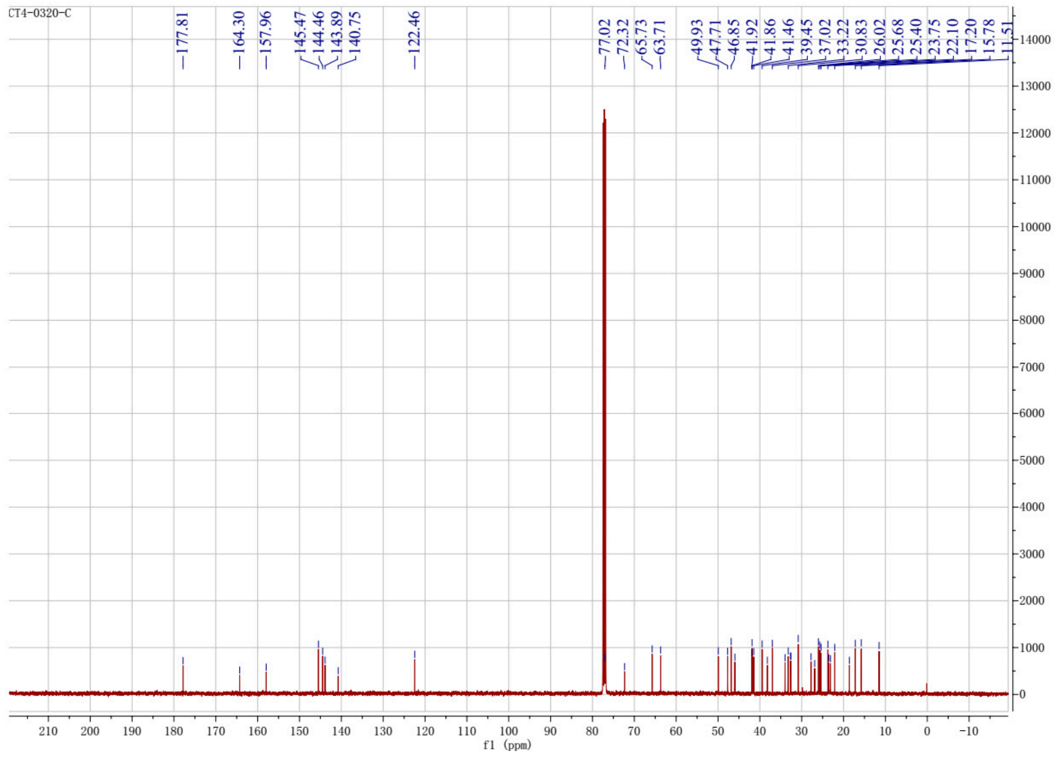

Compound **17**

^{1}H NMR spectra of compound **17**

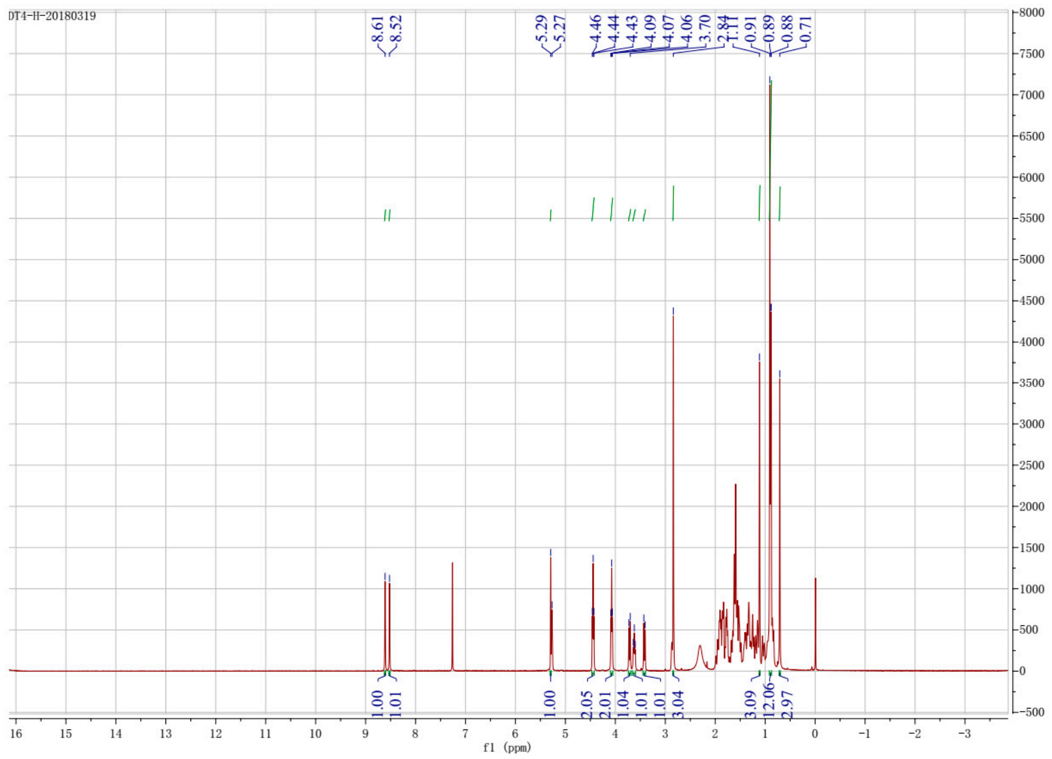

^{13}C NMR spectra of compound **17**

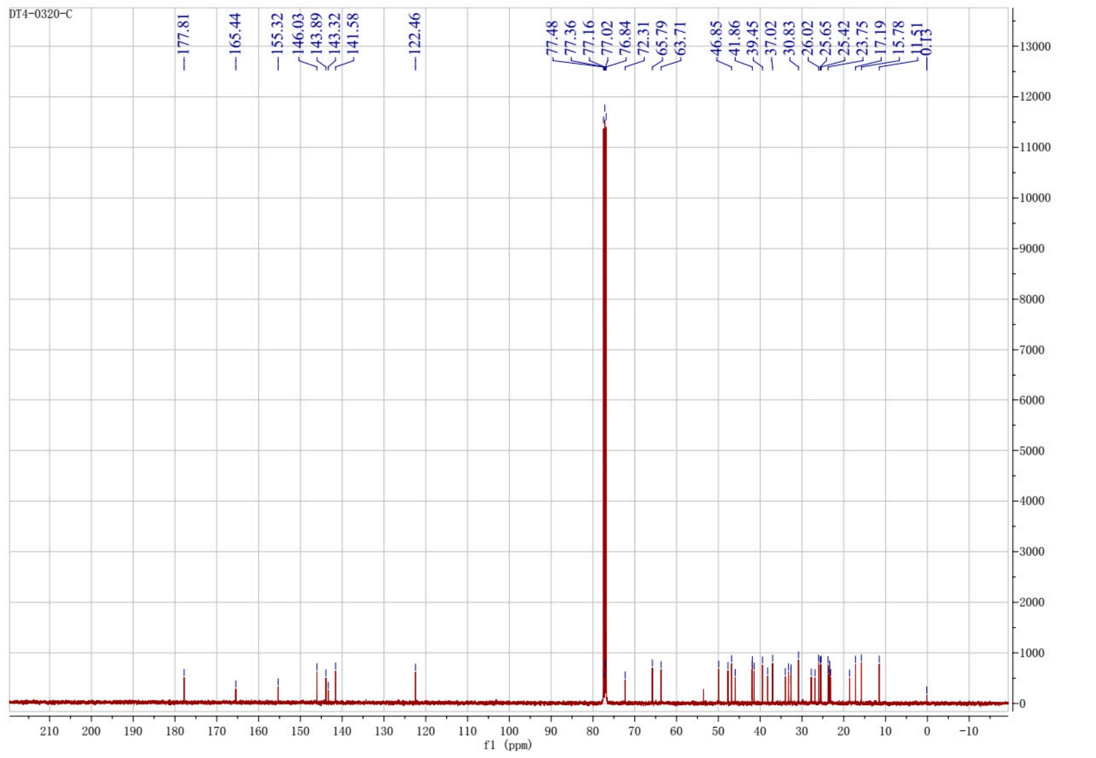

Compound **18**

^{1}H NMR spectra of compound **18**

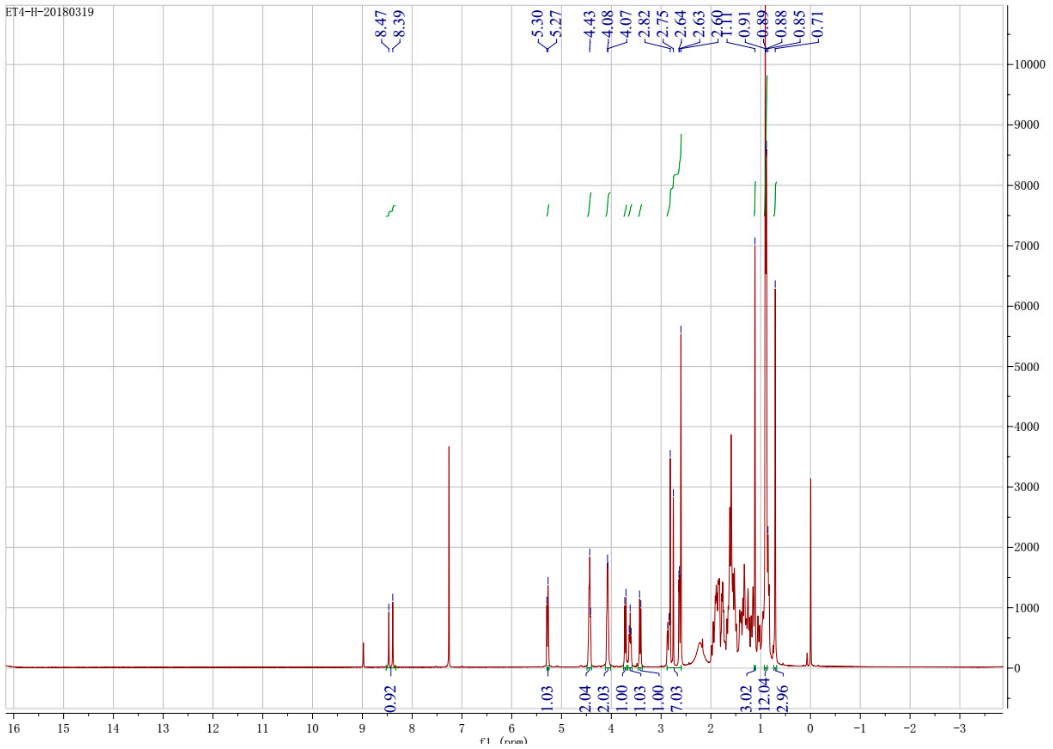

^{13}C NMR spectra of compound **18**

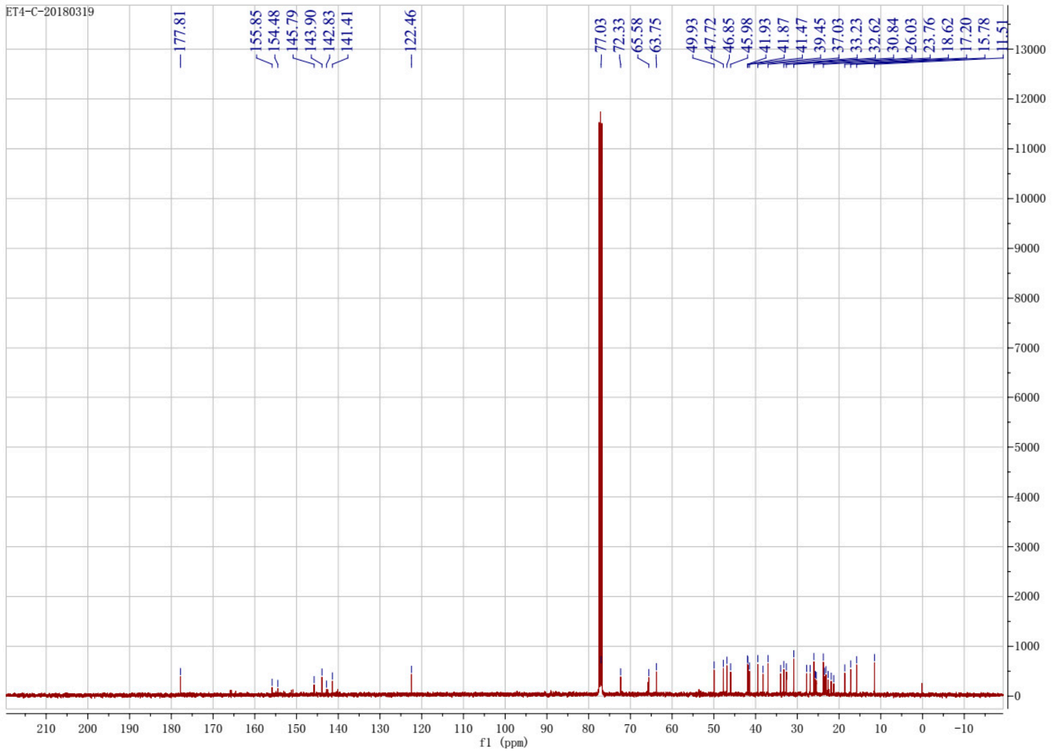

Compound **19**

^{1}H NMR spectra of compound **19**

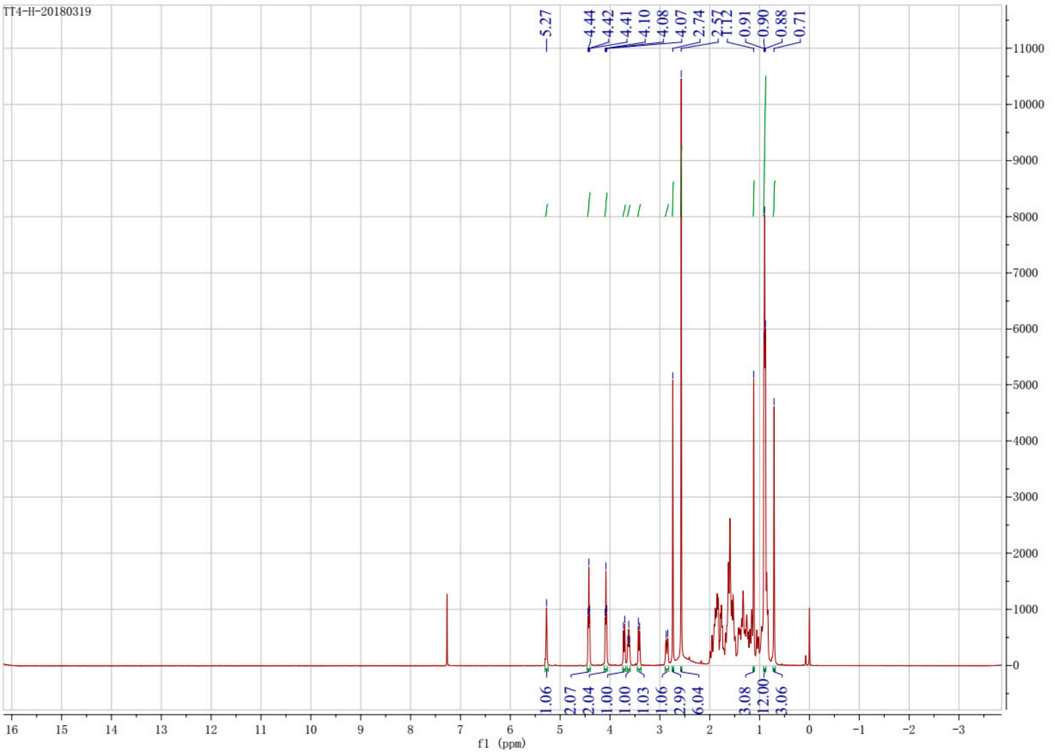

^{13}C NMR spectra of compound **19**

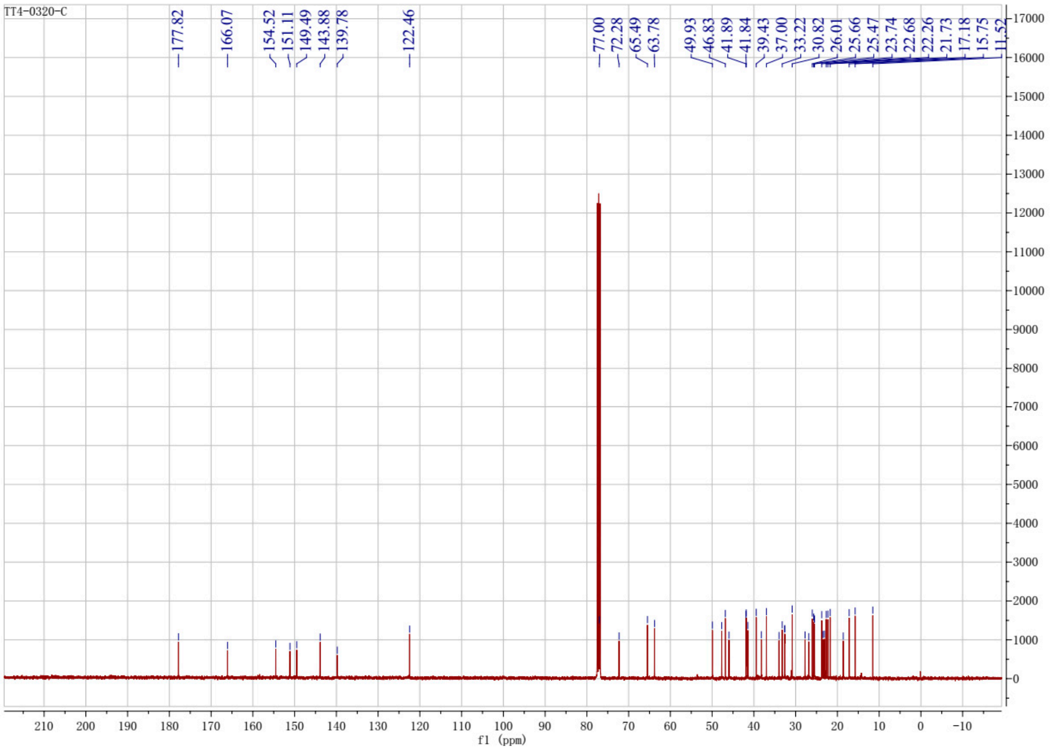

Compound **20**

^{1}H NMR spectra of compound **20**

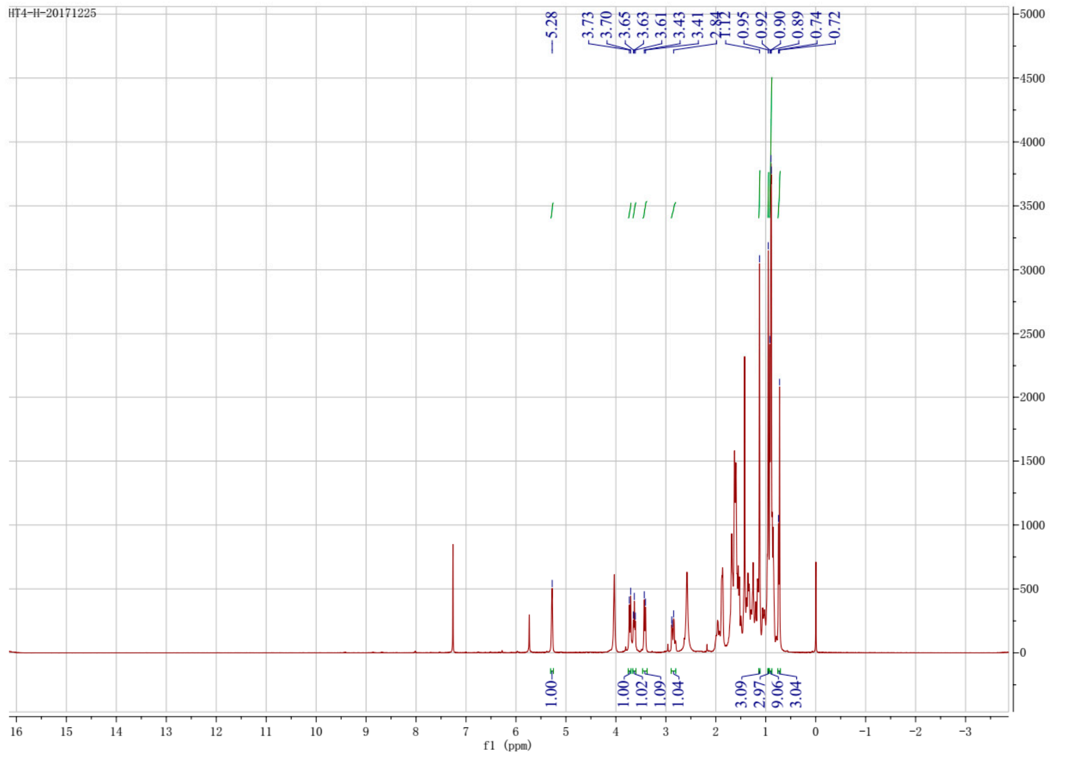

^{13}C NMR spectra of compound **20**

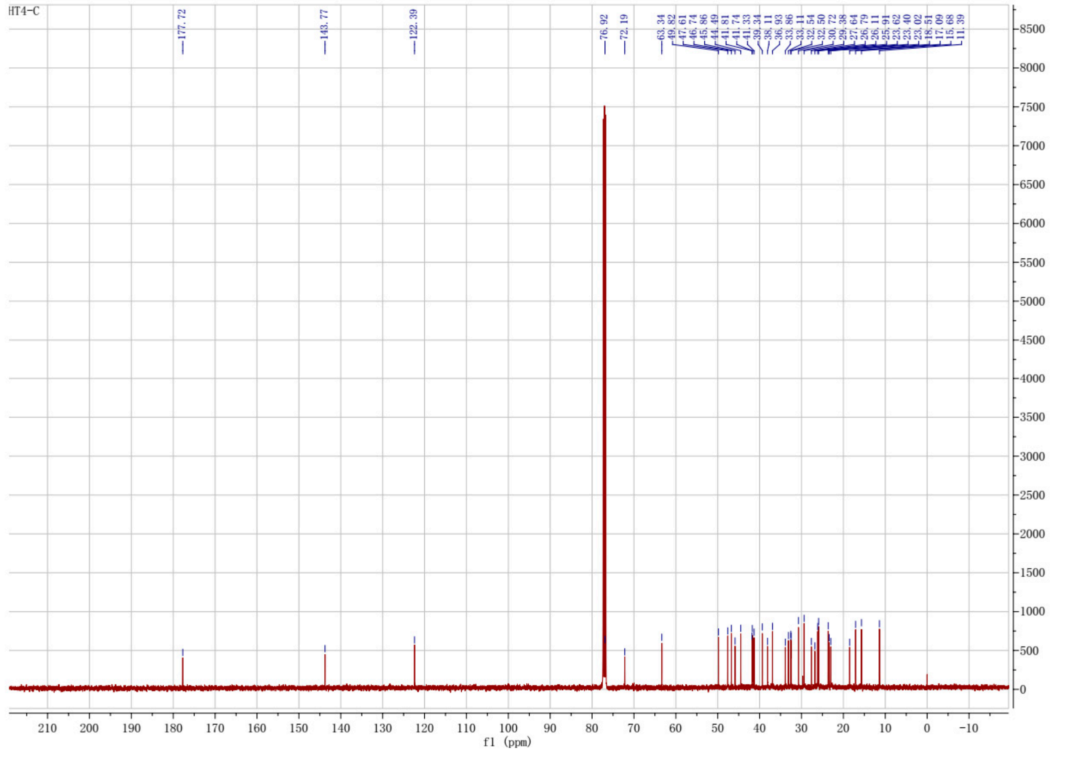

Compound **21**

^{1}H NMR spectra of compound **21**

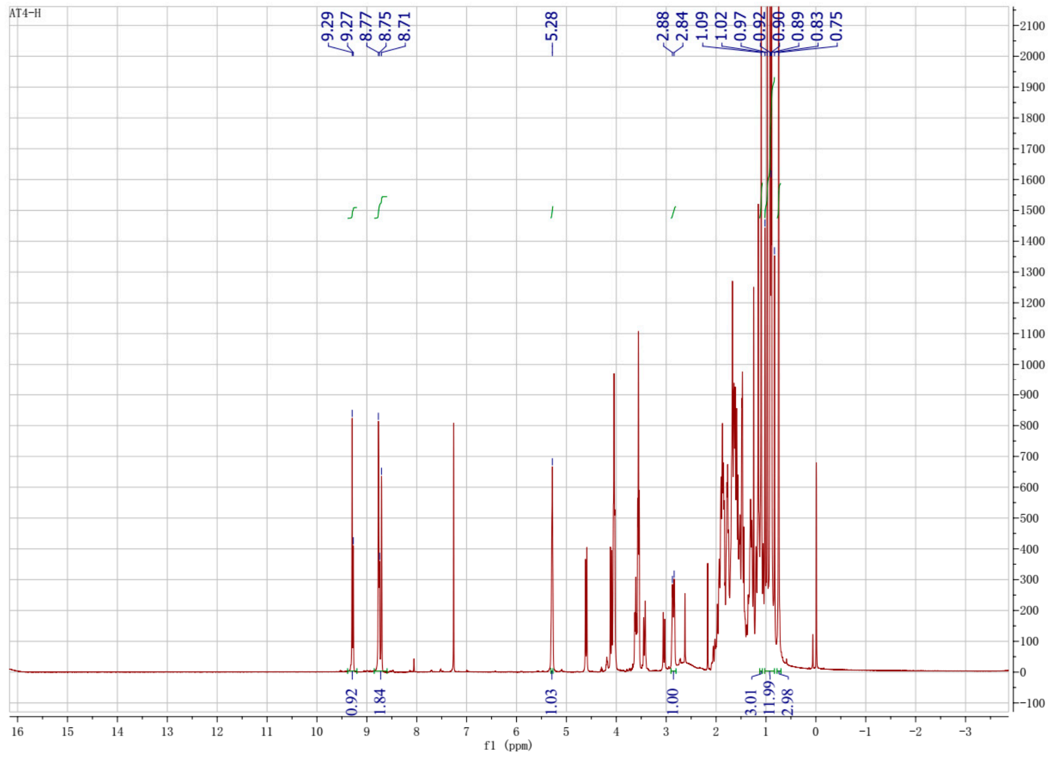

^{13}C NMR spectra of compound **21**

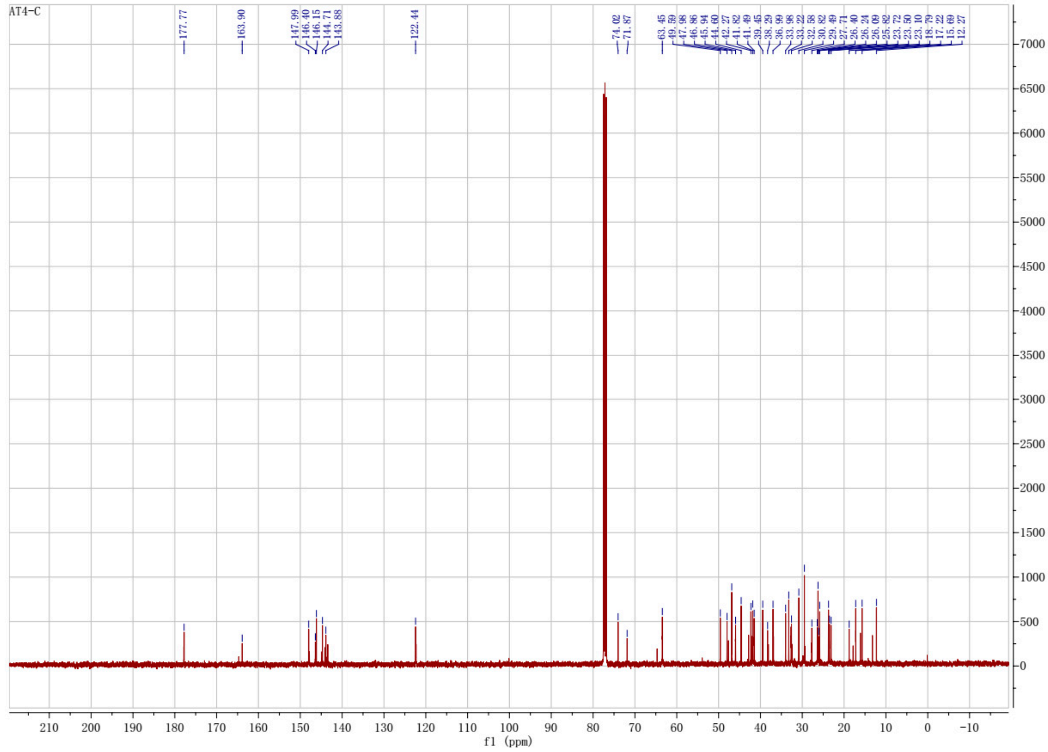

Compound **22**

^{1}H NMR spectra of compound **22**

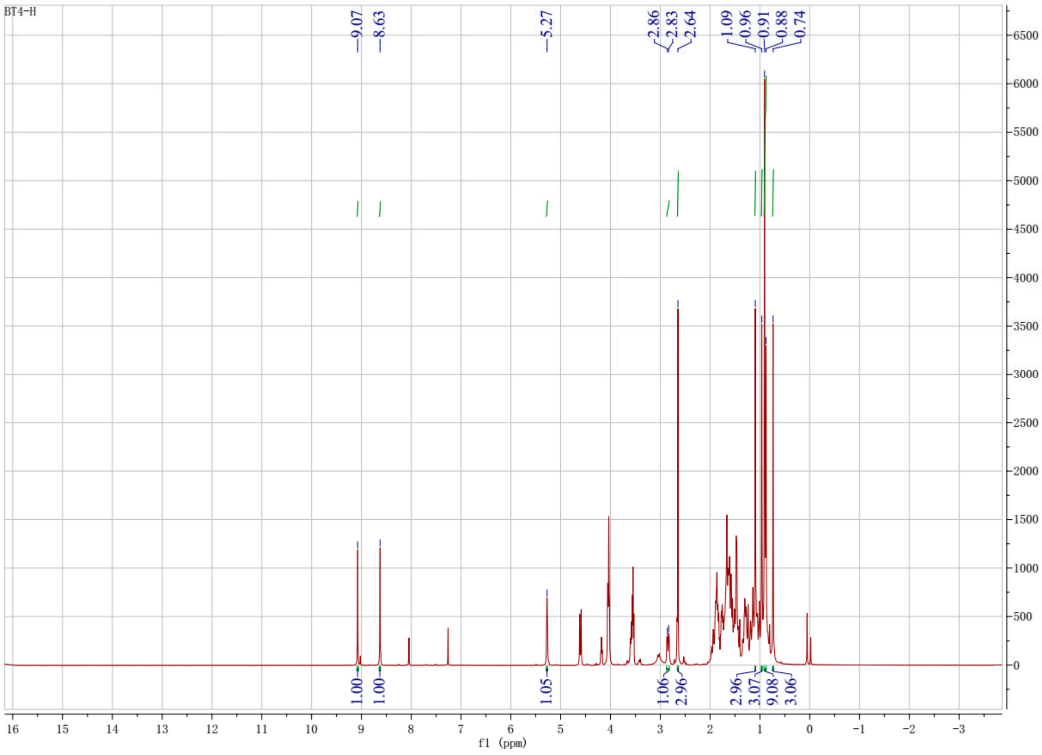

^{13}C NMR spectra of compound **22**

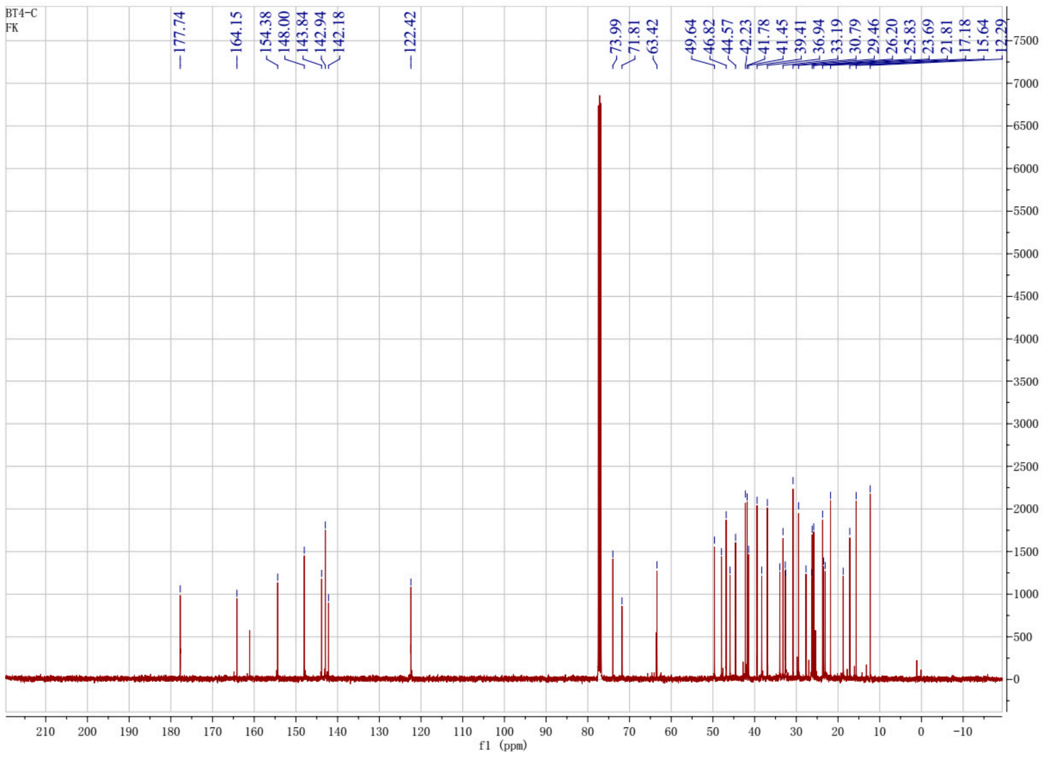

Compound **23**

^{1}H NMR spectra of compound **23**

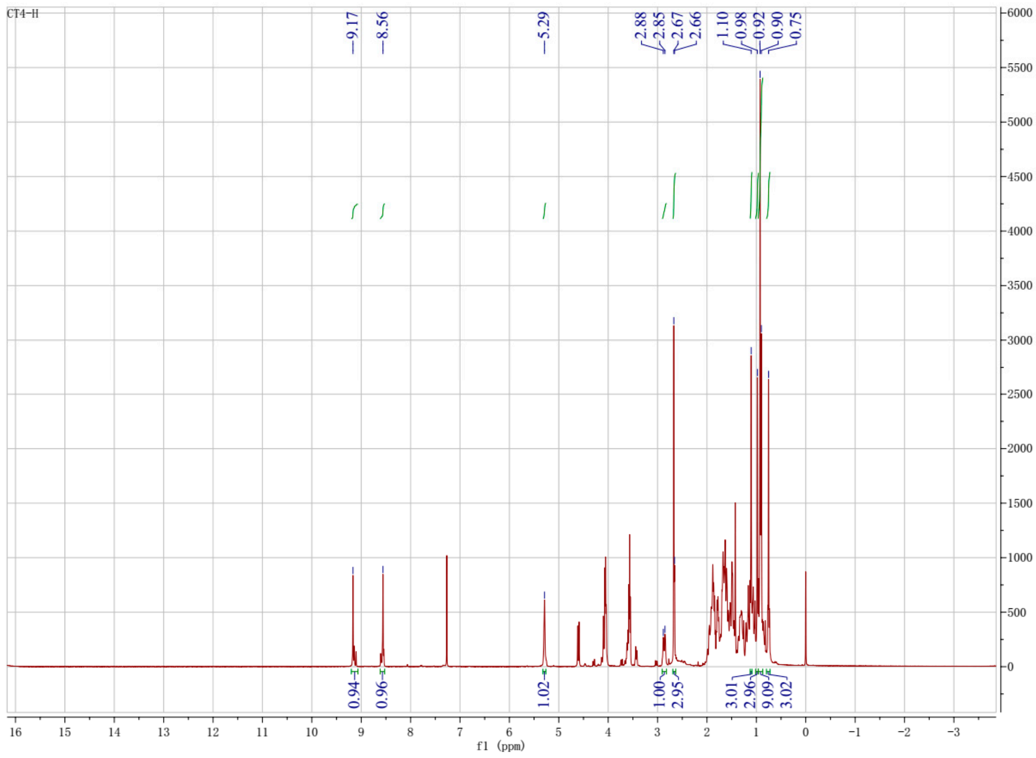

^{13}C NMR spectra of compound **23**

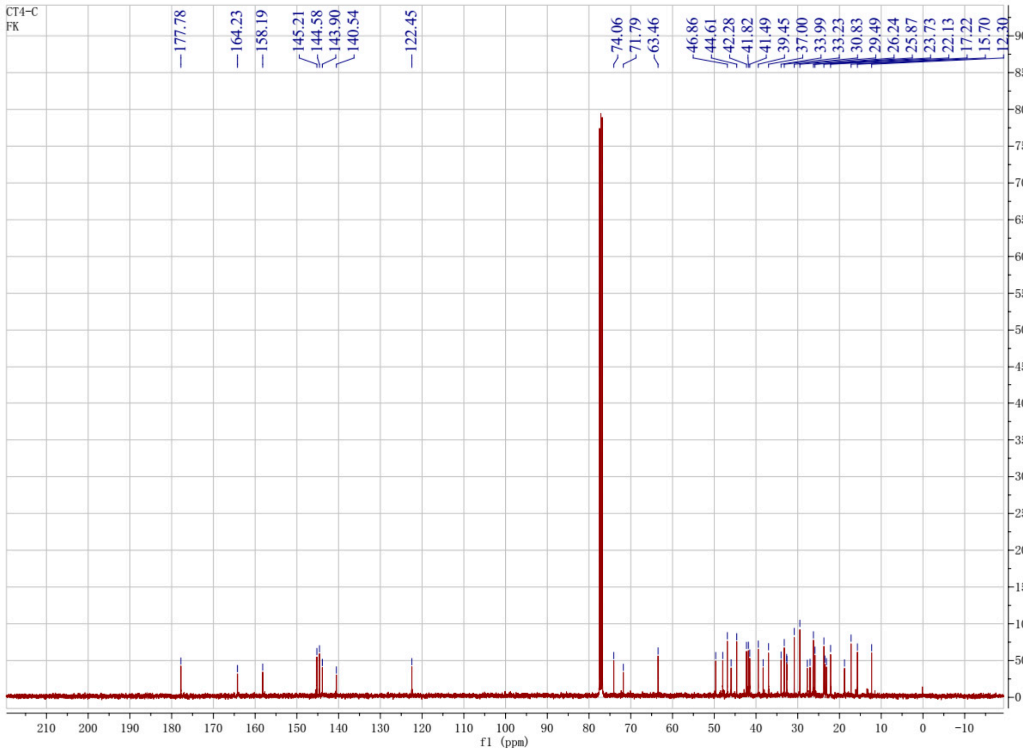

Compound **24**

^{1}H NMR spectra of compound **24**

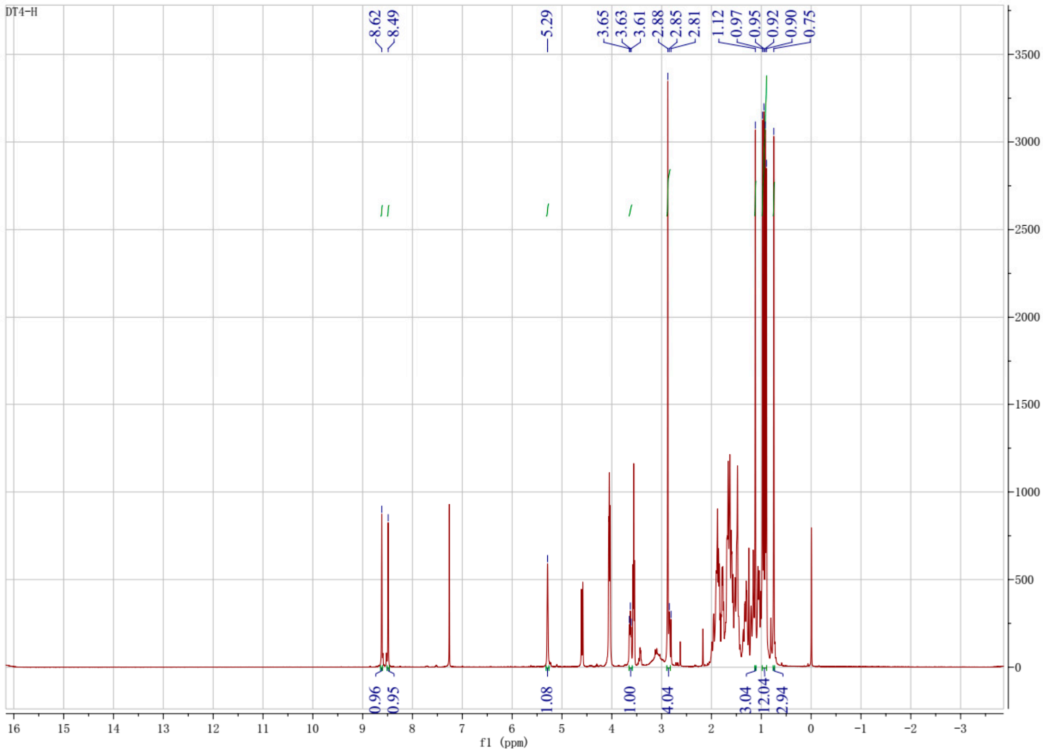

^{13}C NMR spectra of compound **24**

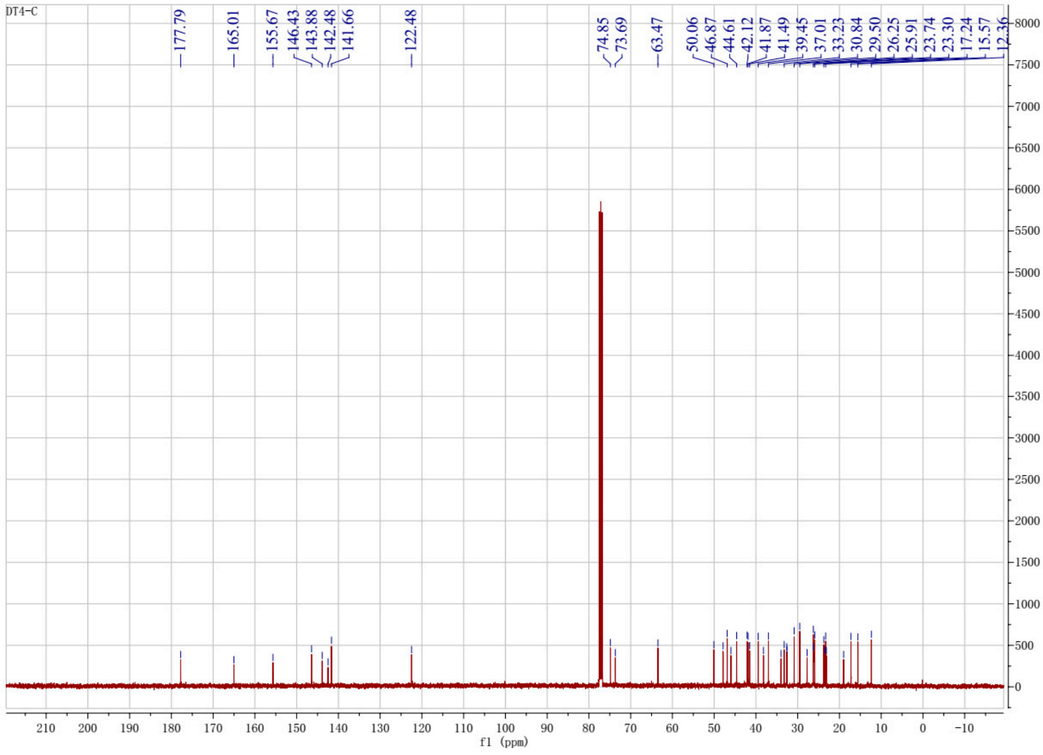

Compound **25**

^{1}H NMR spectra of compound **25**

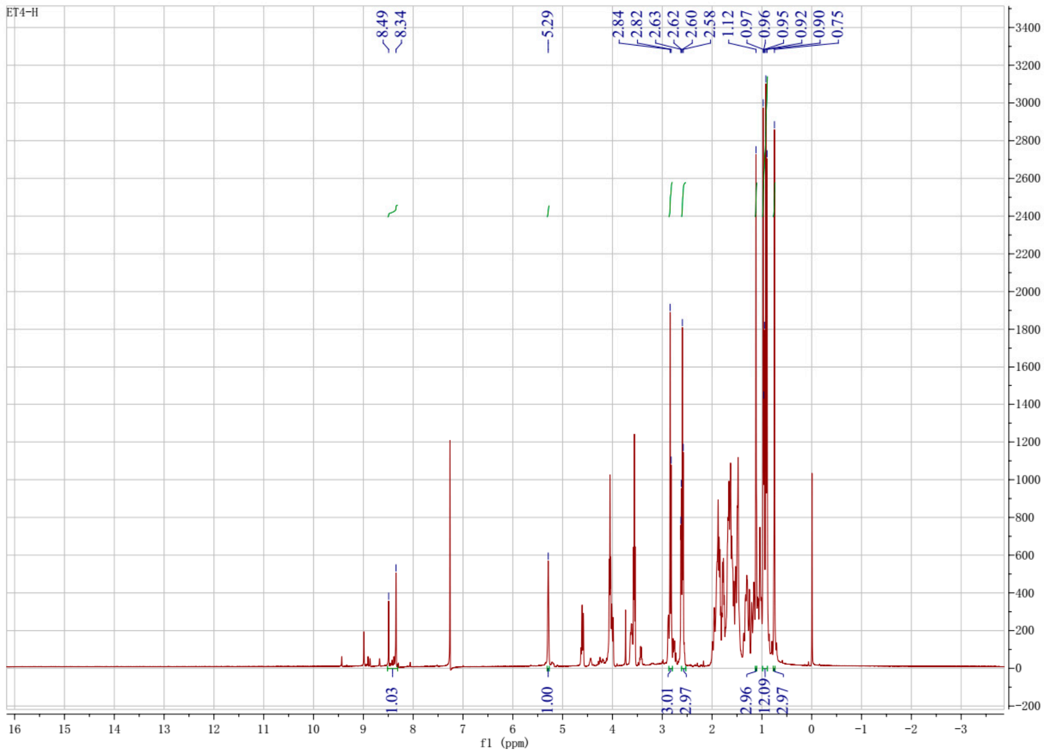

^{13}C NMR spectra of compound **25**

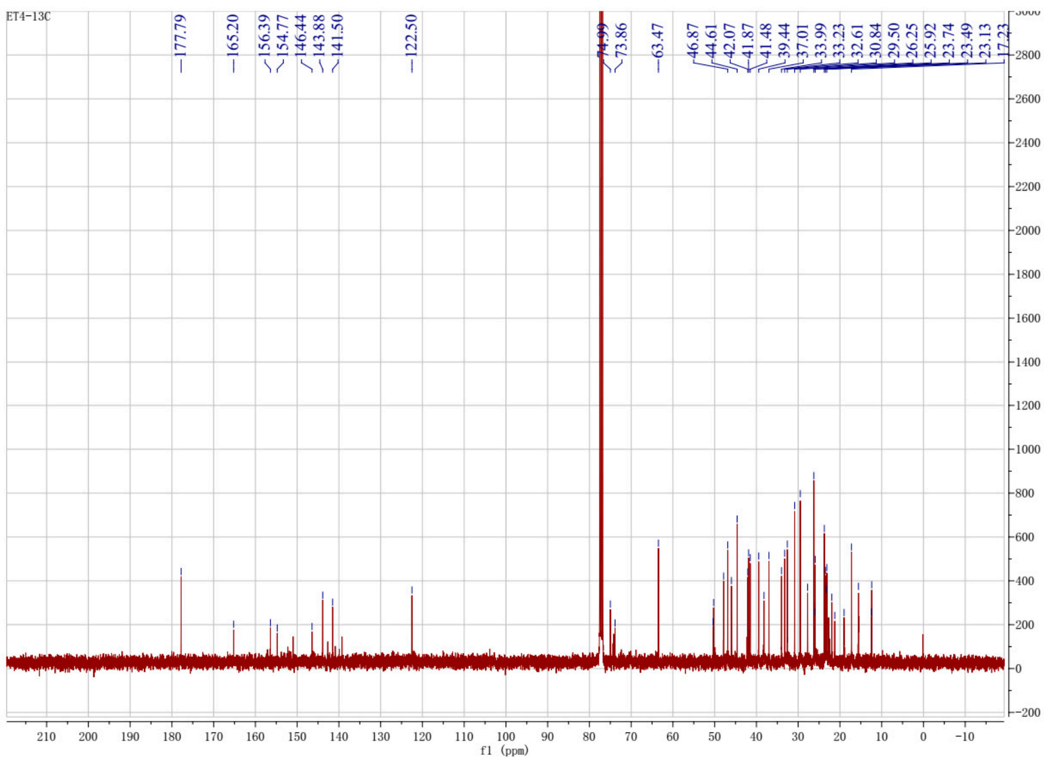

Compound **26**

^{1}H NMR spectra of compound **26**

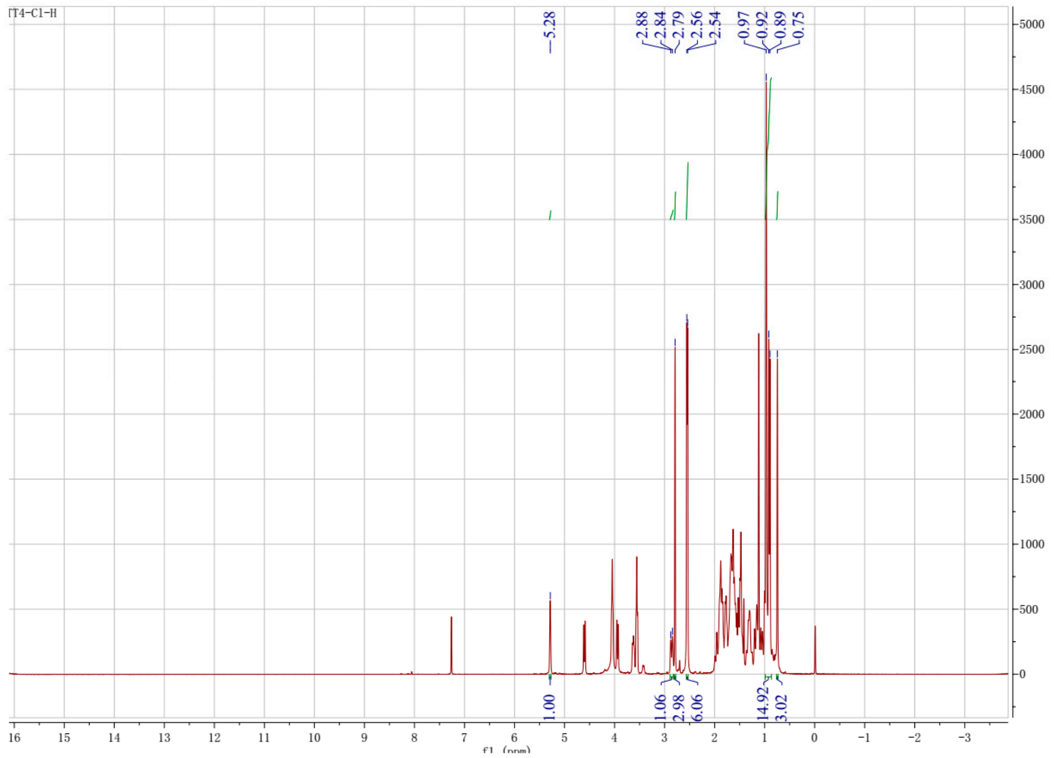

^{13}C NMR spectra of compound **26**

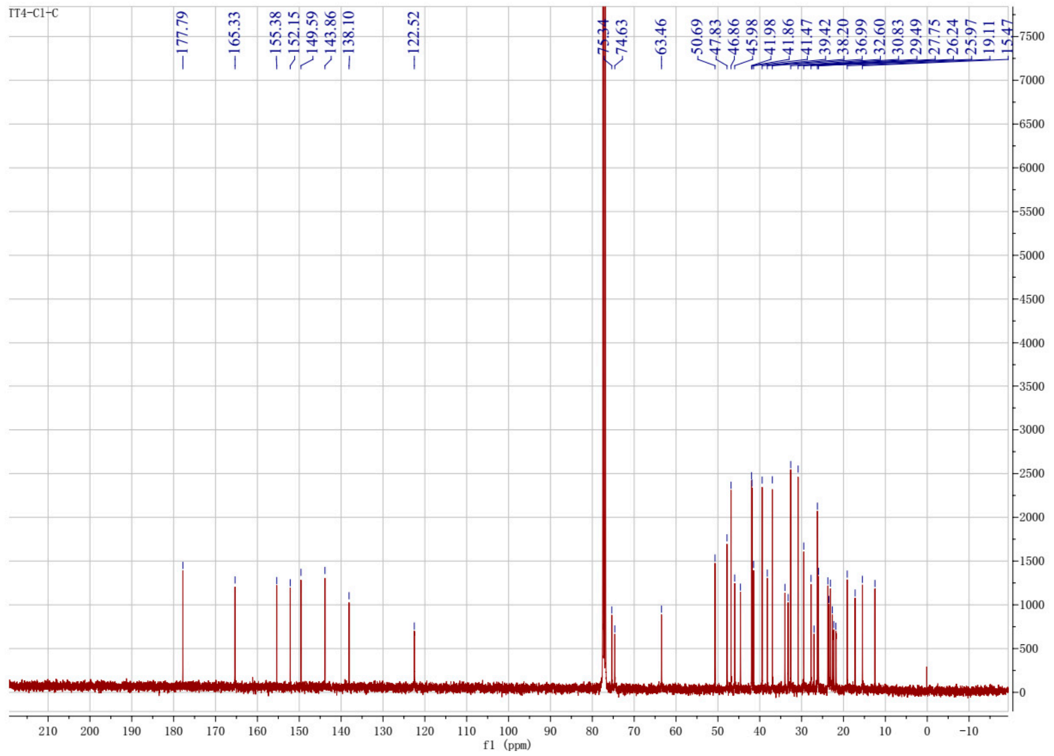

Supplement: Supplementary file 1 [file ijms-19-02994-s001.pdf]
